# Sequencing of d/l-DNA and XNA by Templated-Synthesis

**DOI:** 10.1021/jacs.5c00708

**Published:** 2025-02-11

**Authors:** Saurabh Joshi, Patrick Romanens, Nicolas Winssinger

**Affiliations:** Department of Organic Chemistry, CVU, Faculty of Sciences, University of Geneva, 1211 Geneva, Switzerland

## Abstract

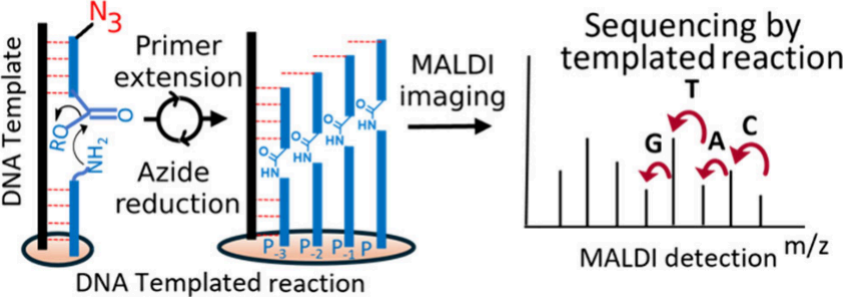

Progress in oligonucleotide sequencing has transformed modern biology and medicine. Here we report a fast and efficient enzyme-free primer extension of PNA with reversible chain termination and its application to DNA and XNA sequencing. The approach leverages activated 4-mer PNAs that react in a templated ligation reaction at μM concentrations within minutes. We demonstrate that the fidelity of this enzyme-free primer extension benefits from reactions performed with a mixture of activated PNAs where every 4-mer has its self-complementary 4-mer. The reactions can be performed using the whole repertoire of 4-mers (256 permutations) in a parallelized manner. Using a primer in combination with its −1, −2, and −3 deletion allows for sequencing by MALDI analysis, using the increment in mass for each nucleobase assignment. Given the enzyme-free nature of this sequencing and the achiral nature of PNA, we further demonstrate that the technology can be used to sequence d- or l-DNA as well as LNA and PNA (XNA).

## Introduction

**Figure 1:**
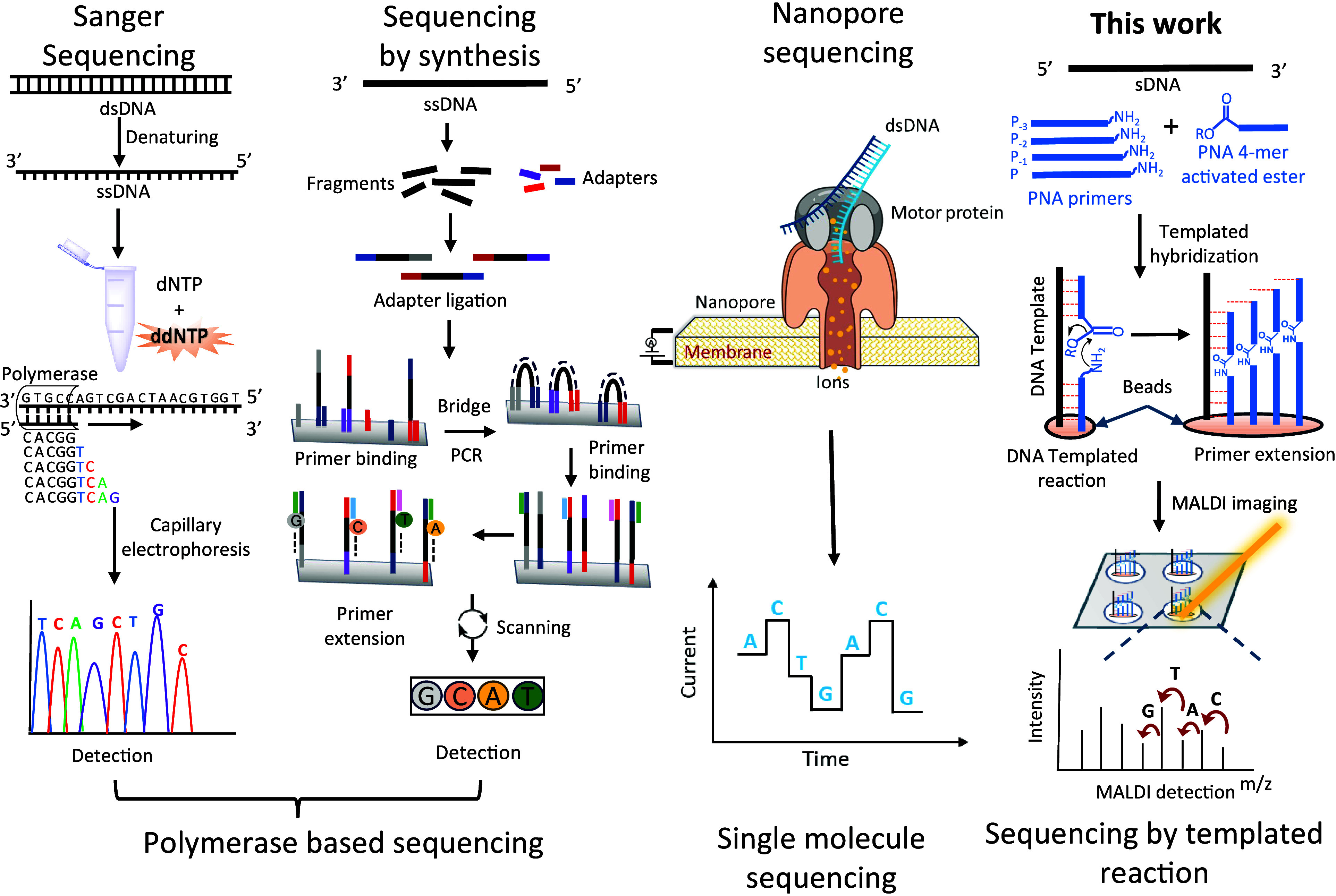
Schematic representation of different generations of DNA-sequencing technologies.

DNA sequencing is a cornerstone of modern research and medicine. The demand for faster, more scalable, and cost-effective sequencing methods has driven the development of a series of transformative innovations. Starting with Sanger sequencing,^1^ advancement such as pyrosequencing,^2^ reversible terminator chemistry^3^ for sequencing by synthesis,^4^ and nanopore sequencing^5^ have dramatically reduced the costs while increasing the speed of sequencing (Figure 1). Among these, polymerase driven primer extension with reversible terminators (sequencing-by-synthesis) is the most broadly adopted technology. In parallel, enzyme-free primer extension has attracted long-standing interest for its likely implication in prebiotic chemistry and emergence of self-replicating systems capable of transmitting genetic information.^6,7^ Initially explored by Orgel and colleagues to understand the origins of life, this method faced significant limitations, including low yield, prolonged reaction time and poor fidelity.^8^ Later the chemistry was revisited by Richert and co-workers demonstrating acceleration in the reaction when downstream binding oligonucleotides sandwich the primer extension reaction;^9^ continuous flow of activated monomers to overcome the hydrolysis during primer extension^10^ and dinucleotides to increase affinity to the template.^11^ In parallel, Szostak and co-workers found that 5′-5′imidazolium bridged dinucleotides, which are formed spontaneously in the nucleotide activation reaction, improved fidelity and reaction rates by virtue of higher template-affinity and geometrical positioning of the reactive species.^12−14^ In addition, Richert and co-workers also recognized the potential of enzyme-free primer extension for genotyping^15^ and sequencing with reversible termination.^16,17^ A limitation remains the reaction time, requiring 12–20 h for each cycle with a very large excess of nucleotides and poor reaction profiles for the weakly pairing bases, although modified derivatives were shown to alleviate this limitation.^17^

In contrast, several DNA-templated reactions with longer probes have been shown to proceed within minutes at very low concentrations, benefiting from high effective concentrations in stable hybridization complexes.^18−20^ With single nucleotides, the dissociation constant (>2 mM) requires high concentrations of activated monomers in the primer extension reactions.^21^ Conversely, templated reactions (ligation or transfer reactions) have been shown to be possible at much lower concentration, provided the hybridization yields sufficiently long-lived hybridization complex relative to the reaction rate.^22^ Aside from its application in sensing,^23,24^ such templated reactions have also been used to make functional materials and nucleic acid polymers unrelated to DNA.^25−31^

Seeking a compromise between enzyme-free primer extension reaction that provides single nucleotide sequencing information but requires long reaction times and templated ligation reactions that are faster but proceed only on predefined sequences, we reasoned that reactions with 4-mer probes might enable fast primer-extension chemistry and could provide sequencing information by mass spectroscopic analysis (MALDI) if the primer is used in conjunction with −1 to −3 nucleotide deletion such that the primer extension on the mixture of primers yields the mass increments of single nucleotide additions. We opted for PNA based on the high stability of the PNA:DNA duplex and the simplicity of its chemistry (Figure 1, this work).^32−34^

## Results and Discussion

### Validation and Optimization of Primer Extension

**Figure 2:**
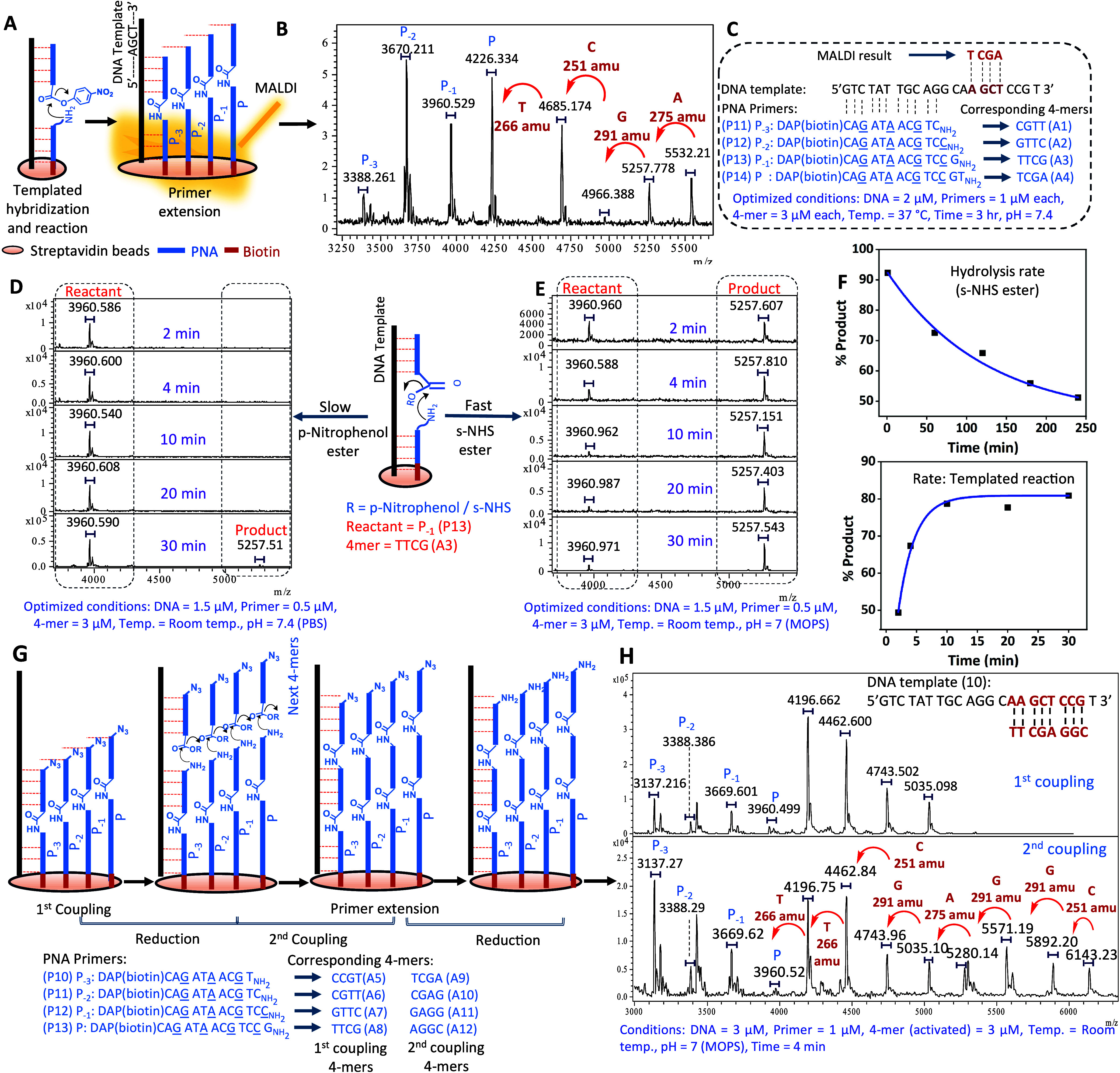
Initial screening and validation of primer extension reactions. (A) Pictorial representation of primer extension via a DNA templated reaction. (B) Observed MALDI spectra. (C) Primers and 4 mers sequences. Underline nucleobases are serine modified. (D and E) Product formation at different time points with p-Nitrophenol vs s-NHS ester. (F) Plots of reaction over time for the hydrolysis and templated reaction of s-NHS ester-PNA. (G) Pictorial representation of two cycles of primer extension with reversible termination. (H) MALDI result with sequencing information. PNA sequences are written as C→N. MW is given for the first monoisotopic peak, and the isotopic distribution is shown with the brackets.

Based on the high stability of the PNA:DNA duplex, we anticipated that a biotinylated 14-mer PNA primer (Tm > 60 °C) would suffice to capture a DNA analyte on streptavidin beads or surfaces for primer extension chemistry.^34^ Precedents in templated reactions using DNA:PNA duplexes suggested that a 4-mer PNA should afford efficient primer extension using amide bond formation.^35^ Recognizing that primer extension by four nucleotides alone would lack the resolution necessary for sequencing, we reasoned that using the primer and its −1, −2, and −3 deletion should afford the ladder of products corresponding to molecular weight increment of each nucleobase in the sequence. To test this hypothesis, a 22nt DNA fragment was captured by a mixture of four primers (P, P_−1_, P_−2_, P_−3_) immobilized on streptavidin beads and treated with a mixture of four 4-mer PNAs, activated at the C-ter as *p*-nitrophenyl ester and capped at the N-ter. Analysis of the product by MALDI revealed a partial conversion of the 4 primers to the sequence-specific products, enabling a straightforward sequence deconvolution (Figure 2 A–C, see Figure S1 for sequencing workflow and explicit structures of products as well as their molecular weight). Encouragingly, there was no cross-reactivity leading to scrambled sequencing information, but the reaction was deemed too slow (ca. 50% conversion after 3 h). Further testing of the primer-extension reaction in buffer, without beads, showed the same results (Figure S2), indicating that surface or bead confinement was not at the origin of slow reaction performance. No product was detected in control experiments, without the DNA template, confirming template-specific primer extension (Figure S3).

Next, we screened different carboxylic acid activating agents (EDC/NHS, EDC/s-NHS, and DMT-MM) for templated reactions with in situ activation; however, capped products arising from the N-ter of the PNA reacting with the activating agent were observed (Figure S4). Following an optimization for preactivation of the 4-mer at higher concentration and lower EDC equivalents, we found that 4-mers at 25 mM concentration can be activated within 30 min using 4 equiv of EDC and s-NHS at pH 7 (>95% based on trapping of the active ester with BuNH_2_, Figure S5). Dilution of the active ester into the buffer of the primer extension reaction to 3 μM delivered primer extension with *t*_1/2_≈ 2 min and no observed capping product due to the lower final concentration of EDC. The reaction was dramatically faster than the *p*-nitrophenyl activated ester one which, under the same conditions, afforded marginal product after 30 min (Figure 2D and E). Measuring the rate of s-NHS ester hydrolysis indicated a *t*_1/2_> 250 min, thus very slow relative to the rate of primer extension (Figure 2F).

To achieve a longer sequence read, iterative cycles of primer extension are necessary. To this end we made use of PNA with an azide at the N-ter.^36^ Using the optimized primer extension procedure with s-NHS activation, we performed a first round of primer extension using a mixture of four 4-mers corresponding to the four primers for 4 min. The streptavidin beads were washed with buffer then treated with PMe_3_ (500 mM) in buffer for 10 min, washed again, and treated to a second cycle of primer extension, again with a mixture of four 4-mers corresponding to the four different primers (Figure 2G). Analysis of the MALDI spectra clearly revealed the ladder of products corresponding to the sequence of DNA that templated the reaction (Figure 2H). It should be noted that it is possible to analyze the streptavidin beads directly on the MALDI plate with the addition of the matrix (DHB) in a solution containing 0.1% TFA.^37^ The acidity of the solution evidently dissociated the PNA from its complementary DNA and streptavidin beads. Given the resolution of MALDI imaging (50 μm), a density of 40,000 sequences/cm^2^ could be analyzed.

### Fidelity of Primer Extension

**Figure 3:**
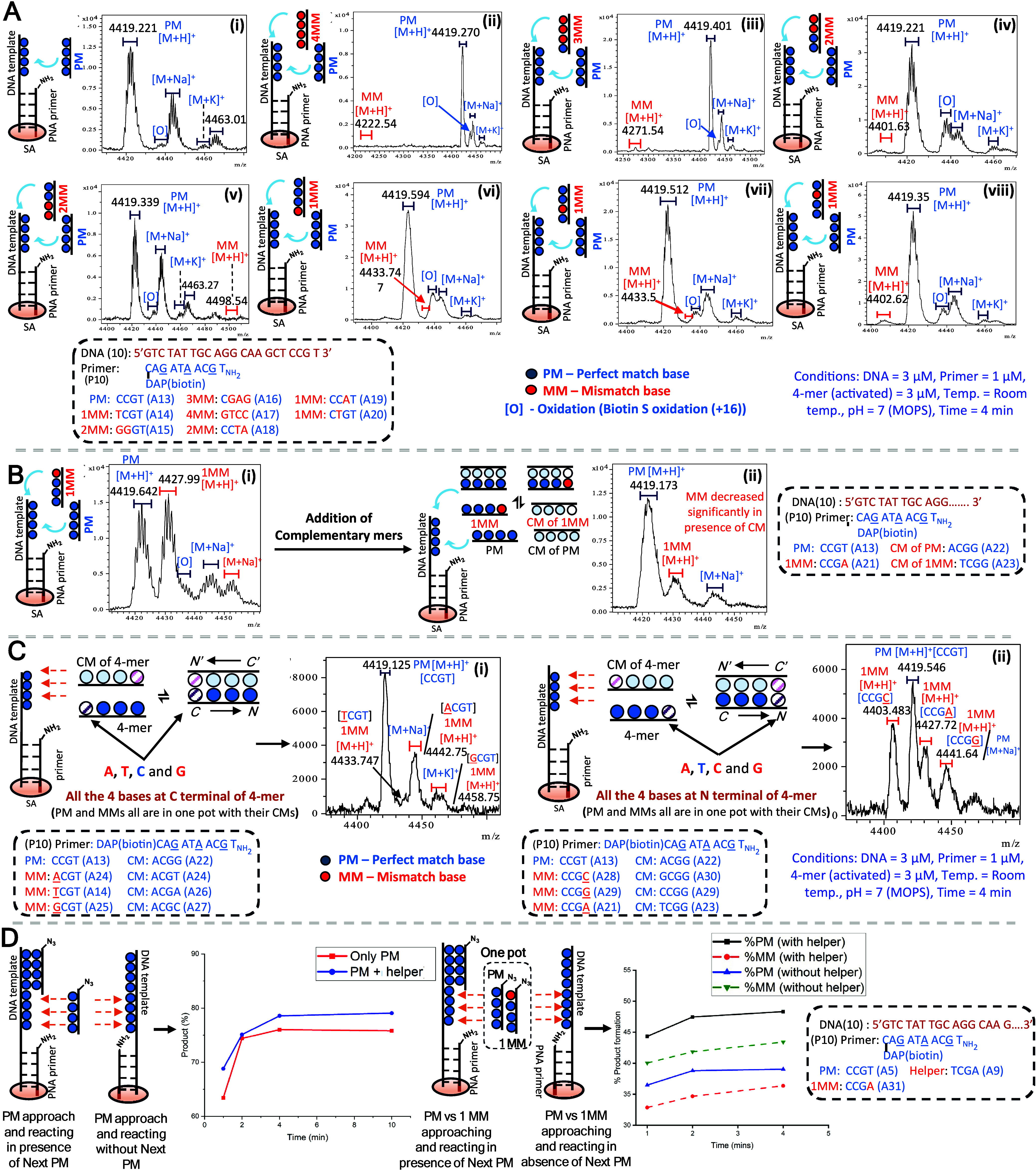
Selectivity profile. (A) Competition between perfect match(PM) vs different mismatches (MM) 4-mers (i–viii). (B) Competition between perfect match (PM) vs terminal 1MM (i), MM peak decreased significantly in the presence of complementary matches (CM) (ii). (C) Competition between PM vs 1 MMs (all four bases at 1MM site) in one pot with their CMs. 1MM at C-ter (i) and N-ter (ii) of 4 mers. (D) Representation and% product formation of confined hybridization of PM and PM vs 1MM followed by primer extension in the presence and absence of helper (next PM). PNA sequences are written C→N. Molecular weights are given for the first monoisotopic peak, and the isotopic distribution is shown with the brackets.

To assess the fidelity of the primer extension reaction, competition reactions between the perfect match and mismatch 4-mer, varying the position of the mismatch, and the number of mismatched nucleobases were performed. When more than a single nucleobase is mismatched, the primer extension proceeds with very high fidelity (Figure 3A, i-v). The peaks of the perfect match (PM) are observed predominantly as the M+H^+^ peak with smaller peaks for M+Na^+^ and M+K^+^. Also, a small set of peaks corresponding to the biotin oxidation (+16 Da) are observed. For the competition reactions where only a single nucleobase is mismatched (Figure 3A, vi-viii and 3B, i), the position of this mismatch (MM) has a significant impact. Only a mismatch at the terminal position yielded an ambiguous product (1:1 PM:MM peak intensity; Figure 3B, i). Considering that all internal mismatched nucleobases affect the overall π-stacking of the duplex relative to a terminal mismatch that can more freely accommodate the mismatched interaction, it is not surprising to observe a different behavior for the terminal nucleobase. However, the observed fidelity relative to the terminal nucleobase is clearly not sufficient for sequencing.

We next asked if the presence of complementary 4-mer PNA would impact the yield and fidelity of the primer extension reactions. A complementary 4-mer results in equilibria between the PM 4-mer hybridizing to the template or its complementary PNA 4-mer which might slow the reaction. Previous studies^38^ indicated that the dissociation constant (*K*_D_) of 4-mer PNA is in the 3–15 μM range with dissociation speed (*k*_off_ ∼ 0.3–1.5 s^–1^). Thus, the system will be highly dynamic. Hybridization to the template uniquely adds π-stacking interactions at the site of ligation. Pleasingly, we found that the reaction performed equally well in the presence of complementary 4-mer (Figure S6). Using the worse-case scenario identified in the single nucleotide mismatch analysis (terminal T/A mismatch), the PM:MM peak intensity progressed from 1:1 to 4:1 in the presence of CMs (Figure 3B, ii). This improvement can be rationalized by a shift in the equilibrium from MM^PNA^:template to its complementary 4-mer (MM^PNA^:CM). This scenario does reflect the more complex situation of the template presented with all permutations of 4-mers which will de facto have their complementary sequence present in the mixture. It should also be noted that similar observations have been made by Szostak and co-workers studying 5′-5′imidazolium bridged dinucleotides.^39^ We further investigated this reaction with all possible mismatch at the C-ter (G:A vs G:C, G:T, G:G; Figure 3C, i) and at the N-ter (A:A vs A:C, A:T, A:G; Figure 3C, ii). Gratifyingly, the PM was the highest intensity peak in these experiments as well, allowing unambiguous product assignment.

Next, we examined the ability of a sequence with a terminal mismatch to engage in a second cycle of primer extension. The first cycle was performed in the absence of complementary 4-mers (CM) to obtain a 1:1 mixture of PM and MM product. The second round of primer extension with the next PM 4-mer showed a 6:1 ratio for the extension of the PM primer versus the MM primer (Figure S7). The result indicates that a sequence with terminal MM will not participate in primer extension as efficiently, thus reducing the propagation of misinformation for downstream sequencing. The nature of the blocking group at the N-ter was also investigated, demonstrating that Fmoc and azide (N_3_) yielded comparable results (Figure S8).

Considering the scenario where the whole repertoire of 4-mers is used, we further investigated the impact of an additional 4-mer hybridizing adjacent to the 4-mer participating in the primer extension reaction (Figure 3D). This further improved the rate of reaction and fidelity of primer extension (Figure 3D), consistent with prior work.^9,40^ Finally, we tested the distance dependence of primer extension reactions, comparing the selectivity in competition reactions of PM 4-mers hybridizing adjacent to the primer or skipping 1 or 2 nucleotides. The product of the adjacent hybridization was strongly favored, as could be anticipated on the basis of preorganization, proximity and π-stacking in the hybridization (Figure S8).

### Primer Extension with Complex Mixture and Sequencing d/l DNA, LNA and PNA

**Figure 4:**
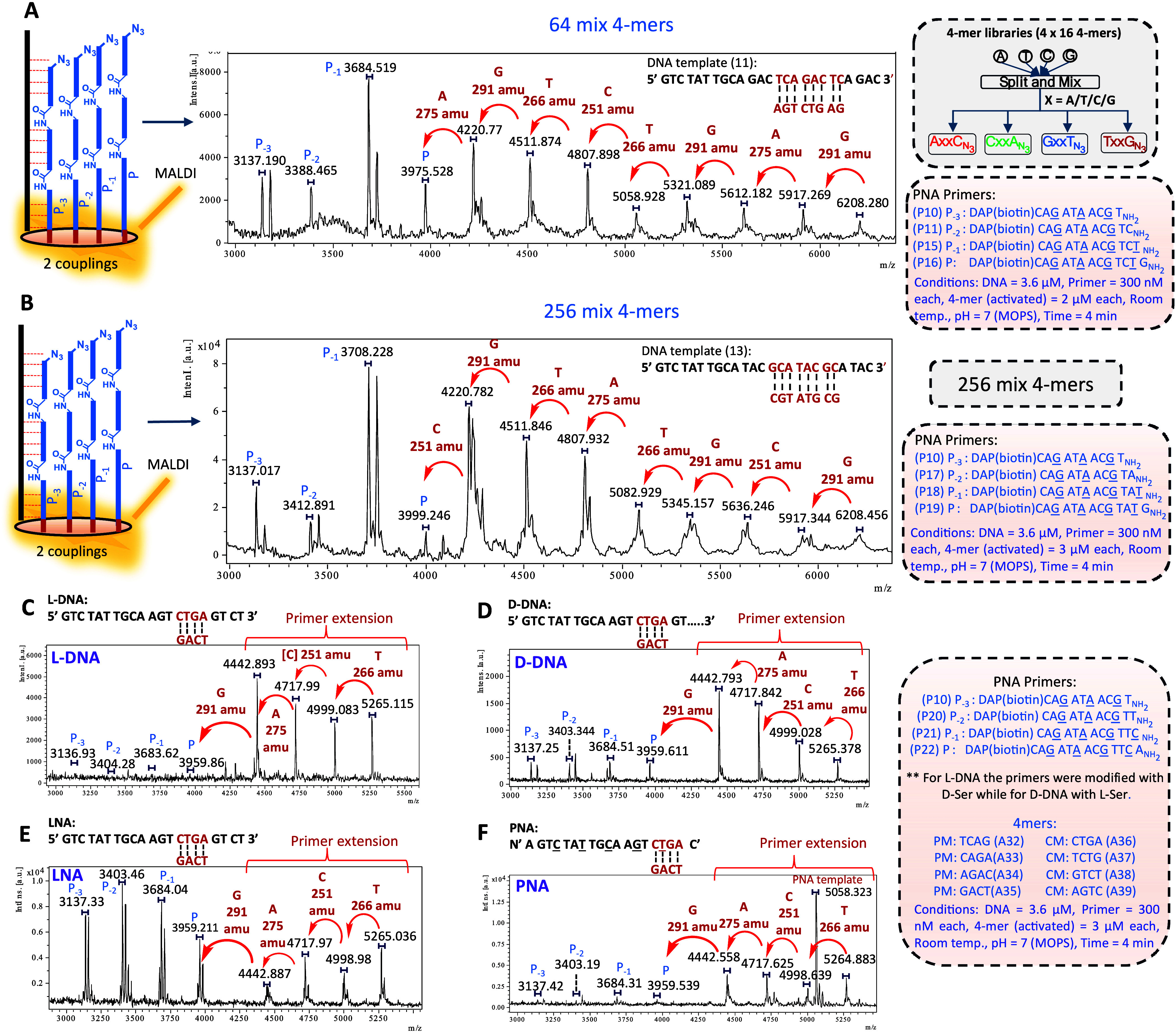
Sequencing nucleic acids. (A) Two consecutive couplings with 64 compound library (4 × 16 4-mers) followed by sequencing DNA template. 4-mer libraries were synthesized by split and mix method. (B) Two consecutive couplings with 256 4-mer mix followed by sequencing the DNA template. Primer extension with different nucleic acid templates, (C) l-DNA, (D) d-DNA, (E) LNA and (F) PNA. Pure 4-mers with their CMs were used. 4-mer sequences are written C→N-ter. Molecular weights are given for the first monoisotopic peak, isotopic distribution shown with the brackets.

To investigate the impact of more complex mixtures of 4-mer PNAs on the performance of enzyme-free primer extension reactions, we used mix-and-split synthesis to obtain a library of 64 PNAs where each member has its PM complement (AxxC-N_3_: GxxT-N_3_, CxxA-N_3_: TxxG-N_3_, Figure 4). Using the optimized procedure, the DNA was captured with the set primers (P, P_–1_, P_–2_, and P_–3_, 300 nM each) and loaded onto streptavidin beads. The beads were treated with the library of activated 4-mers (2 μM each, 128 μM total concentration) for 4 min, washed, treated with PMe_3_ to reduce to azide, and then treated with the library of activated 4-mers. MALDI analysis yielded spectra with clearly discernible peaks corresponding to the stepwise mass increment of the target sequence (Figure 4A). Three other sequences were tested in parallel and afforded comparable results (Figures S9–S11). The peak intensity decreases with the length of the sequence, consistently with the lower ionization efficiency of longer sequences. The fact that the primer extension reactions do not go to completion under these conditions results in the ladder of products that corresponds to the sequence. We found that a fraction of primer, once loaded on the streptavidin bead, always failed to react. We speculate that crowding effect on the beads may be at the origin of this unreactive fraction. Fortuitously, it facilitates sequence analysis without resorting to partial capping. We next performed a primer extension reaction with a full set of all possible 4-mer PNAs (mixture of 256 sequences; Figure 4B). The sequence could be unambiguously assigned based on the mass differential of the peaks (see Figure S12 and Supporting Information for detailed analysis of MM). The experiment explores a complex mixture of possible mismatches, yet, the PM peak is the most intense relative to possible mismatches. Collectively, the results demonstrate that primer extension can be achieved with good fidelity in 4 min.

Based on the achiral nature of the PNA 4-mer used, the same chemistry should work identically on l-DNA. However, the primers that were used contain chiral PNA residues^22,41^ that hybridize exclusively to d-DNA.^42,43^ We thus prepared the mirror image PNA primers and tested the primer extension. As shown in Figure 4C and D, the l-DNA yielded results comparable to those of d-DNA, as could be anticipated, but only with the appropriate PNA primer chirality (d-PNA hybridizes to l-DNA). Thus, the chirality of DNA can be determined based on the chirality of the primer and the sequence assigned using a single chemistry, irrespectively of DNA’s chirality. We further compared sequencing of d- and l-DNA with the full set of 4-mers (256 permutations) using matched chiral primers to obtain sequencing information with high fidelity (Figures S13 and S14). We next asked if the same strategy could be used to sequence other oligonucleotide polymers (XNA). We tested the primer extension with LNA and PNA. Both XNA showed primer extension yielding unambiguous sequencing information from the MALDI spectra (Figure 4E and F), as could be anticipated for an enzyme-free process dependent only on the effective molarity of reactive partners upon hybridization.

Collectively, the results demonstrate that different oligonucleotides (d/l-DNA, LNA, PNA) can be sequenced by primer extension using PNA. The readout by MALDI provides sequencing information by mass increment and is amenable to parallelization with a density of 30–50 μm/sequence. Further improvement in the method should be possible with noncanonical nucleobases.^44−46^ Such artificial nucleobases should further improve the fidelity of the sequence and may prove important for AT rich sequences, which was not thoroughly investigated in the present work. The achiral nature of PNA does not discriminate between d- or l-DNA; however, the primer can be engineered to discriminate DNA’s chirality using gamma-modified PNA^42^ which imparts chirality to the PNA primers. l-DNA is attracting interest for orthogonal data storage^47^ and as biostable aptamers^48−50^ but are limited by the ability to sequence this mirror image DNA.^49,51^ Finally, the sequencing method is akin to a transcription of DNA into PNA, while the sequencing of PNA may enable the amplification of PNA; both should be of interest for the development of PNA-based aptamers.^25,27^ This work parallels the recent report of template-directed synthesis of acyclic l-threoninol nucleic acid.^52^ It should also be noted that PNA has been postulated as a prebiotic intermediate to an RNA world.^53−55^ While there is no evidence for azide functioning as a reversible chain terminator in prebiotic chemistry, the efficiency and fidelity of the templated primer extension with a complex mixture of PNA 4-mers are highly pertinent. It should be noted that MALDI is routinely used in clinical chemistry^56^ and primer extension reactions have already been analyzed using this technique.^57^

## Conclusions

This work demonstrates the potential of PNA-based primer extension for sequencing DNA, its mirror image (l-DNA), and other oligonucleotides with varied backbone chemistries, including LNA and PNA. The versatility in the polymers that can be sequenced is an inherent feature of enzyme-free primer extension chemistry, further enhanced by the achiral nature of PNA. These attributes position the technology as a promising tool for analyzing sequence-defined oligonucleotides without prior knowledge of their chirality or backbone structure, making it particularly relevant for artificial encoding systems or the search for evidence of life. MALDI analysis is compatible with a dense array format for parallelization of sequencing, and the resolution of latest generation instruments (30 μm with up to 40 000 kDa isotopic resolution) should enable sequence reads much longer than presently demonstrated using an older generation MALDI. A fast cycle time in the primer extension chemistry was achieved using the 4-mer PNA fragment activated at the C-ter with s-NHS and an N-ter azide for reversible termination. A set of four primers with sequential deletion is used to obtain the ladder of products corresponding to the single nucleotide increment. It should be noted that azide reduction is well-known to be compatible with the hybridization duplex and is at the core of the success of sequencing-by-synthesis. Additionally, performing primer extension with a complete library of 256 activated 4-mer PNAs is feasible, and simplified system studies indicate that mixtures improve the fidelity. This fidelity enhancement arises from competition among complementary 4-mers in solution and the stabilization provided by downstream hybridization during the extension reaction, which confines the reaction and enhances the stability.

## Methods

### Synthesis of PNA

Protected PNA monomers were synthesized by previously established protocols^41,58,59^ and oligomers were prepared using solid phase peptide synthesis, as previously reported.

### 4-mer Activation

Crude PNAs were purified as a mixture by HPLC and lyophilized. The 4-mers thus obtained were taken up in DMF (25 mM final concentration), followed by the addition of freshly prepared solution of EDC and s-NHS (400 mM, DMF) at the final concentration of 100 mM each. The reaction was allowed to proceed for 30 min and added directly to the templated primer-extension reactions.

### Templated Chemical Reaction

Primer-extension reaction on streptavidin beads using 4-mer PNA mix (256 mix):

(A) Loading: DNA (3.6 μM final concentration) and primers (300 nM final concentration) were taken in 100 μL of buffer (MOPS, pH7.0) and kept at RT for 10 min for complete hybridization. 20 μL streptavidin beads (Dynabeads MyOne Streptavidin C1 beads of size 1 μm, previously washed with the MOPS buffer) were added into the prehybridized mixture for primer capturing for 30 min. The beads were then washed thoroughly to remove unbound material.
(B) Reaction cycle: The beads (20 μL) in 100 μL MOPS buffer at room temperature were treated with activated 4-mers (final concentration of 3 μM). The reaction was allowed to proceed for 4 min at RT, after which the beads were washed.
(C) Deprotection: Beads from the previous reaction cycle step were resuspended in 20 μL of buffer (MOPS, pH 7.0) and treated with 20 μL of PMe_3_ (1 M in THF). The reduction process was allowed to proceed for 10 min at RT, after which the beads were washed thoroughly.

For the repetitive cycle (2nd coupling): Beads from the deprotection step (Step C) were treated in another reaction cycle (step B).

### MALDI Analysis

Direct analysis of beads:^37^ 5–7 μL of beads in water were spotted and air-dried on the plate. 1 μL of DHB matrix (30 mg of DHB in 1.0 mL of 70:30:0.01 water/acetonitrile/TFA) was spotted on top and the spotted bead solution and air-dried prior to analysis.

Release of the captured primers. The biotinylated primers captured onto streptavidin beads were released by the addition of pure TFA for 1 min with vortex mixing (30 μL of TFA for 10 μL of beads). The TFA solution was filtered and concentrated by using a flow of nitrogen gas. The residues were redissolved in MeCN:H_2_O (1:1) for MALDI analysis.

Solution extracted from beads: 10 μL beads were taken, extracted, and redissolved in 5 μL of MeCN:H_2_O. 0.8 μL of the extracted solution was spotted on the plate followed by addition and mixing of 0.8 μL of DHB matrix (30 mg of DHB in 1.0 mL of 70:30:0.01 water/acetonitrile/TFA) and air dried prior to analysis.

## Data Availability

All the raw data of measurements reported in this work has been deposited on Zenodo (DOI 10.5281/zenodo.14760440).

## Supporting Information Available

Characterization details and spectra of primers and 4-mers used in the studies with explicated structures. (PDF)
