# Supplementary material for: Sequencing of d/l-DNA and XNA by Templated-Synthesis
Source: J Am Chem Soc. 2025 Feb 11;147(7):6288–96. doi: 10.1021/jacs.5c00708 (PMC11848921; doi:10.1021/jacs.5c00708)
Supplement: Supplementary file 1 — ja5c00708_si_001.pdf [file ja5c00708_si_001.pdf]

Supplementary Information

Sequencing of D/L-DNA and XNA by templated-synthesis

Saurabh Joshi[a], Patrick Romanens[a] and Nicolas Winssinger[a]*

[a]Department of Organic Chemistry, Faculty of Sciences, University of Geneva, 1211 Geneva, Switzerland.

*nicolas.winssinger@unige.ch

Table of Content

Abbreviation.......... 2

General methods.......... 3

Oligonucleotide sequences and synthetic procedure.......... 3-4

General schemes for PNA synthesis (S1 -S3) 5

General scheme for DNA templated chemical reaction on solid support.......... 6

Full structural based back calculation of primer extension 6

DNA templated reaction in solution-Scheme, MALDI and Control exp.......... 7

Screening of activating agents and rate of 4-mer activation 8

Primer extension in the presence of CM.......... 9

Primer extension with PM and 1MM followed by next coupling with Next PM.......... 9

Primer extension in the presence of CM (-N_3 capped) 10

Selectivity for PM vs 1MM in presence and absence of CMs (-N_3 capped) 10

Selectivity profile with PM vs 1 and 2 NB skipped PMs..........11

Templated reaction (2 couplings) with 64 mix library and DNA 11,12 and 13.......... 11-12

Zoom in peak view.......... 13

Primer extension of L-DNA and D-DNA template with 256 mix 4-mers 14

Structure of Primers and their characterization.......... 15-33

Structure of 4-mers and their characterization.......... 33-54

LCMS and MALDI spectra of 4 libraries (4x16 compounds) 55-57

PNA monomers (-N_3 protected) – 1H NMR and LC-MS 57-61

Abbreviations

Boc - Tert-butyloxycarbonyl

DCE - 1,2-dichloroethane

DHB - 2,5-Dihydroxybenzoic acid

DIPEA - N,N-Diisopropylethylamine

DMF - Dimethylformamide

DMSO - Dimethyl sulfoxide

ESI - Electrospray ionization

EtOAc - Ethyl acetate

Fmoc - Fluorenylmethoxycarbonyl

HATU - Hexafluorophosphate Azabenzotriazole Tetramethyl Uronium

HFIP - Hexafluoroisopropanol

HOBt - Hydroxybenzotriazole

HPLC - High performance liquid chromatography

LC-MS - Liquid chromatography-mass spectrometry

MALDI - Matrix-assisted laser desorption/ionization

MS - Mass spectrometry

NMP - N-Methyl-2-pyrrolidone

PG - Protecting group

PNA - Peptide Nucleic Acid

TFA - Trifluoroacetic acid

THF – Tetrahydrofuran

PM – Perfect match

CM – Complementary match

1. General methods

All reagents and solvents for the organic synthesis were purchased from commercial sources and were used without further purification. NovaPEG Rink amide resin for PNA synthesis was obtained from EMD Millipore. Streptavidin beads (Dynabeads MyOne Streptavidin C1) were purchased from Thermo Fisher Scientific Inc. with catalog number 65001. HPLC purification was performed with an Agilent Technologies 1260 infinity HPLC using a ZORBAX 300SB-C18 column (9.4 x 250 mm). LC-MS spectra were recorded on a DIONEX Ultimate 3000 UHPLC (condition for elution gradient: 0 min, A:B =100:0; 4 min, A:B = 10:90; solution A: 0.01% aqueous TFA solution; solution B, 0.01% TFA in HPLC grade acetonitrile; flow rate: 0.750 mL/min) with a Thermo LCQ Fleet Mass Spectrometer System using PINNACLE DB C18 column (1.9 µm, 50 x 2.1 mm) operated in positive mode. All the LC-MS spectra were measured by electrospray ionization (ESI), linear gradient 0 to 100%. MALDI-TOF Mass spectra were measured using a Bruker Daltonics Autoflex spectrometer operated in positive mode. The samples were analyzed using 2,5-dihydroxybenzoic acid (DHB) matrix. SFC analyses were performed on a Waters Acquity UPC2 with OD-H column. Retention times (RT) are given in minutes. Thin layer chromatography (TLC) was performed on plates of silica precoated with 0.25 mm Kieselgel 60 F254 from Merck. Flash chromatography was performed using silica gel SiliaFlash® P60 (230-400 mesh) from Silicycle. Automated solid-phase synthesis was carried out on an Intavis AG Multipep RS instrument. Concentration of the PNA or DNA stocks was measured by NanoDropRM 2000c at 260nm wavelength. All DNA sequences were purchased from Eurogentec and were used as received.

Nucleic acid used

Code	Sequence
DNA 10	5' GTC TAT TGC AGG CAA GCT CCG T 3'
DNA 11	5' GTC TAT TGCA GAC TCA GAC TCA GAC 3'
DNA 12/D-DNA	5' GTC TAT TGCA AGT CTGA GT CTG AGT 3'
DNA 13	5' GTC TAT TGCA TAC GCA TAC GCA TAC 3'
L-DNA (DNA 12')	5' GTC TAT TGCA AGT CTGA GT CT 3'
LNA	5' GTC TAT TGCA AGT CTG AGT CT 3'
PNA	C' AG<u>T</u> CT<u>G</u> AA<u>C</u> GT<u>T</u> AT<u>C</u> TGA N'

Synthetic protocols:

Synthesis of PNA oligomers

5.0 mg of Nova PEG® Rink amide resin (0.44mmol/g, NovaBiochem) or 10 mg of chlorotrityl resin (0.22 mmol/g) or 10 mg of wang resin (1.2 mmol/g, NovaBiochem) were swollen in DCM for 30 minutes and washed twice with DMF. Iterative cycles of amide coupling (Procedure 1), capping of the resin (Procedure 4), and deprotection of the protecting group (Procedure 2 or 3) were done to synthesize the PNA probes. The compounds were deprotected and cleaved from the resin using TFA (Procedure 5) and finally purified using HPLC.

Procedure 1 (P1): Amide coupling.

The corresponding protected PNA monomer, (4.0 equiv., 0.2M in NMP) was incubated for 5 minutes with HATU (3.5 equiv., 0.5M in NMP) and base solution [DIPEA, 1.2M (4.0 equiv.) and 2,6-lutidine 1.8M (6.0 equiv. in NMP]. The mixture was then added to the corresponding resin. After 30 minutes, the mixture was filtered, the resin was washed with DMF, and a new premixed reaction solution was added to the resin and let react for another 30 minutes. Finally, the resin was washed with 2x DMF, 2x CH_2Cl_2, and 2x DMF.

Procedure 2 (P2): Fmoc deprotection.

A solution of 20% piperidine in DMF was added to the resin and allowed to react for 5 minutes. The mixture was then filtered, the resin washed with DMF, and the sequence repeated a second time for another 5 minutes. Finally, the resin was washed with 2x DMF, 2x CH_2Cl_2, and 2x DMF.

Procedure 3 (P3): Mtt deprotection.

A solution (made from 244 mg of HOBt in 10 mL of HFIP and 10 mL of DCE) was added to the prewashed resin to reach a volume of 10 mL/g of resin and allowed to react for 5 minutes. The solution was flushed, the resin washed with CH_2Cl_2, and the sequence repeated a second time for another 5 minutes. Finally, the resin was washed with 2x CH_2Cl_2, and 2x DMF.

Procedure 4 (P4): Capping.

The resin was treated with a capping mixture (0.92 mL of acetic anhydride and 1.3 mL of 2,6 lutidine in 18 mL of DMF: 10 mL of solution/g of resin) for 5 minutes. After flushing the solution, the resin was washed with 2x DMF, 2x CH_2Cl_2, and 2x DMF.

Procedure 5 (P5): Cleavage from the resin and final deprotection.

Resin (5.0 mg, 1.0 µmol) was treated with 125 µL of a mixture of TFA and scavengers (440 µL of TFA + 25 mg phenol + 25 µL water + 10 µL tri-isopropylsilane) for 2 hr. The resin was filtered, washed with TFA (50 µL), and the collected fractions of cleavage product precipitated in cold ether (1.5 mL). After centrifugation, the pellet was vortexed again with cold Et_2O (1.5 mL) and centrifuged (14K rpm). The pellet was dissolved in water/acetonitrile (3:1; 1.5 ml) and lyophilized to obtain a white powder.

Procedure 6 (P6): Microcleavage for quality control.

The minimum number of beads were picked up with a pipette plastic tip and transferred to 50 µL of TFA. The solution was left for 1 hr and transferred to 1.0 mL of ether. The ether solution was kept for 5 minutes at -20 °C and then centrifuged for 5 minutes at 14K rpm. The ether supernatant was removed, and the pellet dissolved in 20 µL 1:1 acetonitrile/water, which was then analyzed by MALDI and/or LC-MS.

Procedure 7 (P7): Coupling of Biotin.

To 5.0 mg of resin (0.0022 mmol), a solution of 2.15 mg of biotin (0.0088 mmol, 4 equiv.), 3 mg of HATU (0.0077 mmol, 3.5 equiv.) and 2.3 µL of DIPEA (0.0132, 6 equiv.) in 50 µL of NMP was added. After 30 minutes reaction at room temperature, the mixture was filtered, the resin was washed with DMF, and a new premixed reaction solution was added to the resin and let react for another 30 minutes. Finally, the resin was washed with 2x DMF, 2x CH_2Cl_2, and 2x DMF.

Characterization of PNA.

Characterization of the PNAs was done by MALDI (Bruker Daltonics Autoflex spectrometer with Flex control 3.4 software and analysis with FlexAnalysis 3.4) and/or LC-MS (DIONEX Ultimate 3000 UHPLC with a Thermo LCQ Fleet Mass Spectrometer System using PINNACLE DB C18 column (1.9 µm, 50 x 2.1 mm) with Thermo Xcalibur 2.2.SP1.48 software and analysis with Thermo Xcalibur Qual Browser 2.2.Sp1.48). For MALDI analysis, 1.0 µL of the sample (in either water or water/acetonitrile 1:1) was mixed with 1.0 µL of DHB matrix solution (30 mg of DHB in 1.0 mL of 70:30:0.01 water/acetonitrile/TFA), and the mixture spotted on a MALDI plate. The measurements were done in a positive linear mode. Calibration Standard Peptide II (Bruker LabScape – Daltonics 8222570) used prior to any measurement. For LC-MS analysis, 20 µL of sample in water or water/acetonitrile 1:1 was injected on the LC and further analyzed by MS on a positive mode.

Templated chemical reaction

Solution primer-extension: Different concentrations of DNA, PNA primers and 4-mers were incubated in pH 7.4 buffer for different time intervals at RT or 37 °C. After that 0.8 µL of the solution was aspirated from the reaction mixture and dropped on MALDI plate followed by addition of DHB matrix on top of it in liquid state. Let both mixed solution dry and MALDI MS spectra was recorded.

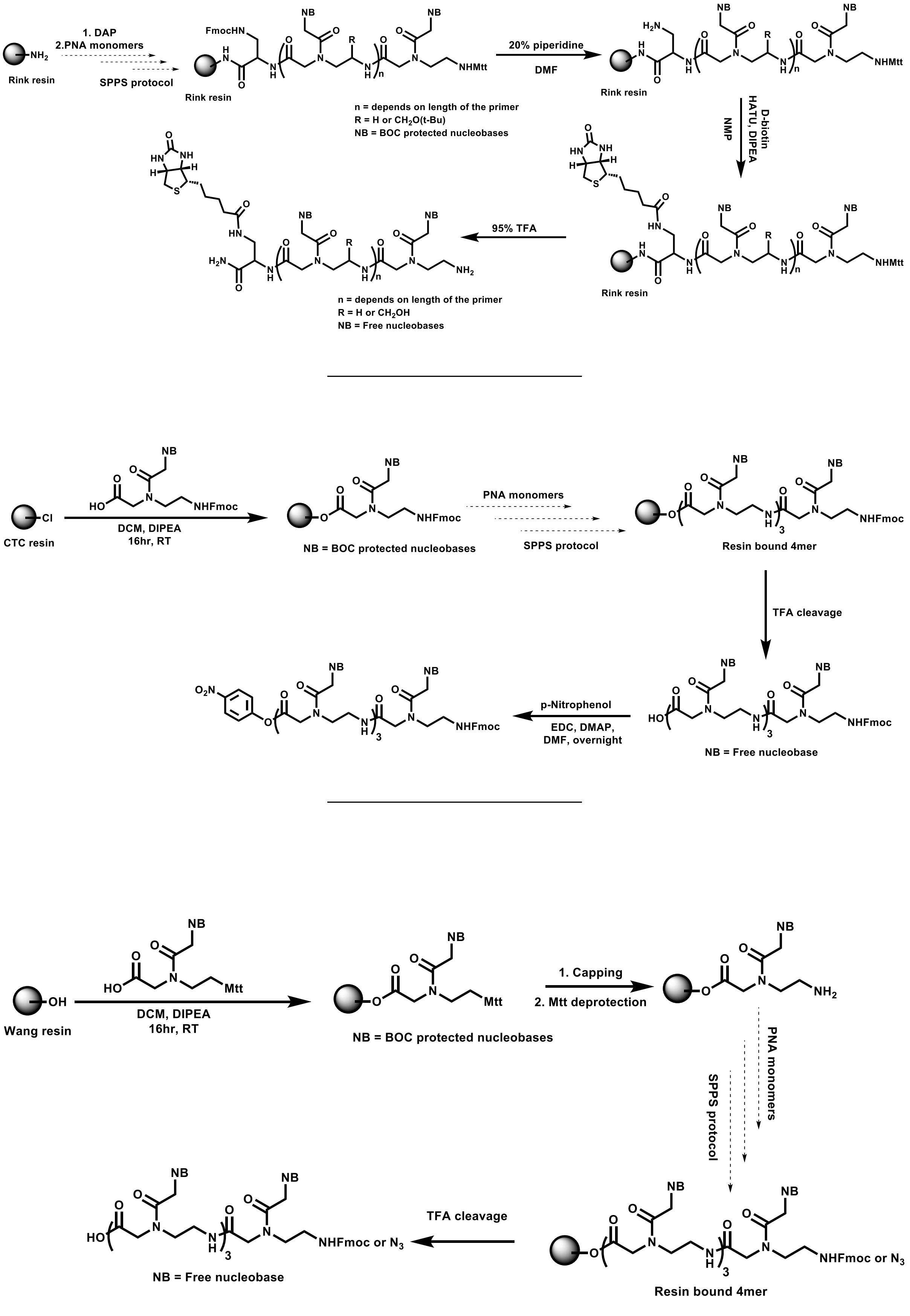

Scheme S1. General scheme for PNA primer synthesis (top), PNA 4-mer synthesis with CTC resin(middle), 4-mer synthesis with wang resin.

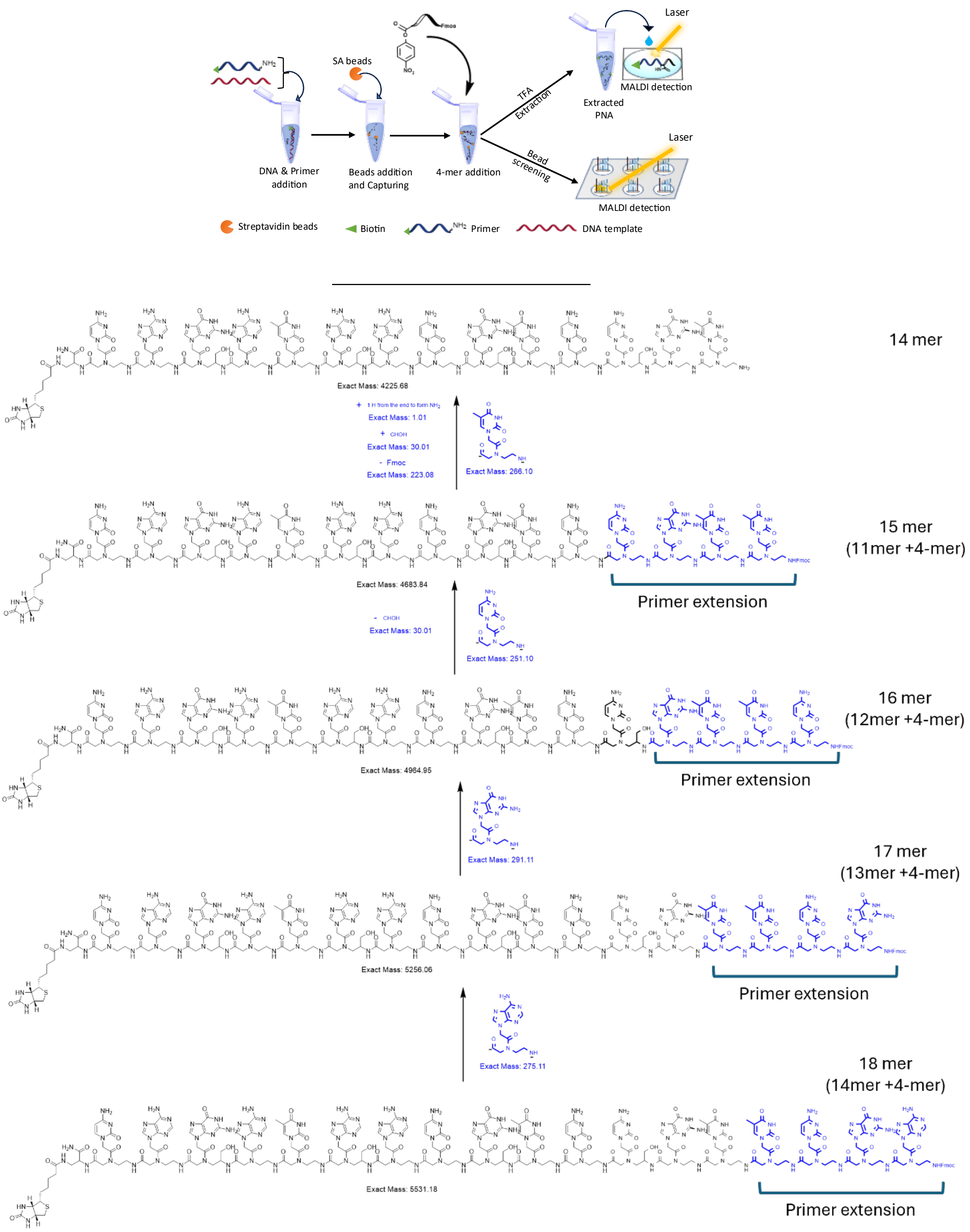

Figure S1. Structure and Calculations - primer extension followed by analysing mass of the incorporated nucleobases in sequential manner

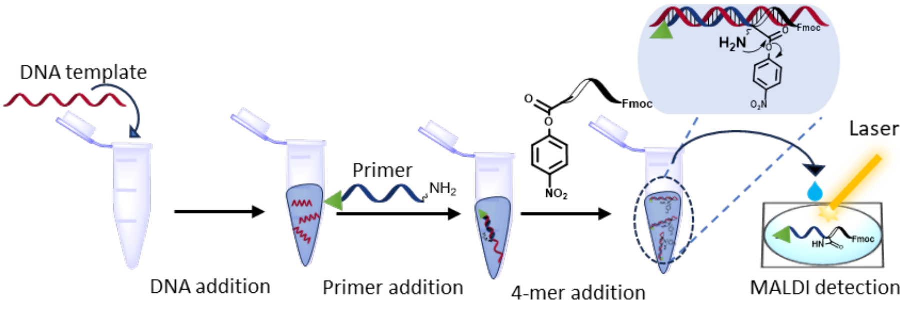

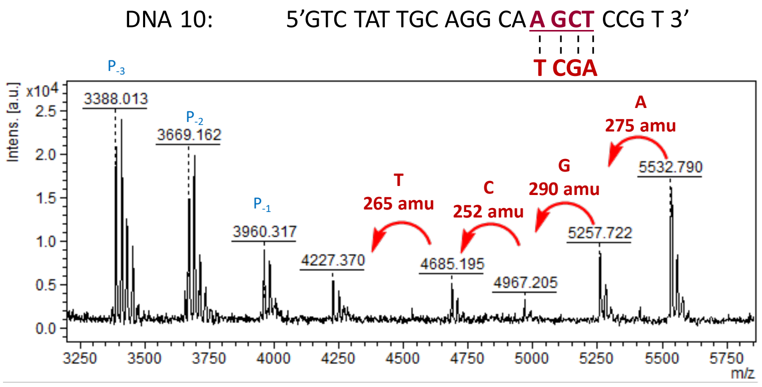

Figure S2. MALDI spectra of DNA templated reaction performed in solution phase to check the hypothesis.

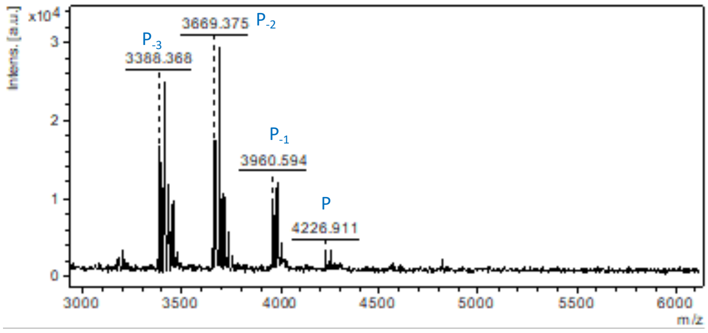

Figure S3: MALDI spectra of DNA templated reaction performed in solution phase without DNA (Control)

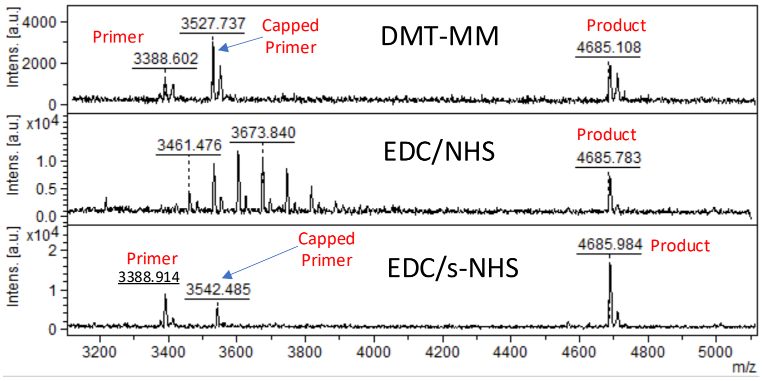

Figure S4. Screening of activating agents for templated reactions. EDC/s-NHS showed the best and clean result with least capping. Reactant (primer) was capped by DMT-MM and EDC. Condition: DNA - 2 µM, Primer - 1 µM, 4-mer - 3 µM, 20 mM EDC/15 mM s-NHS or 20 mM NHS, 40 mM DMT-MM, overnight and room temperature.

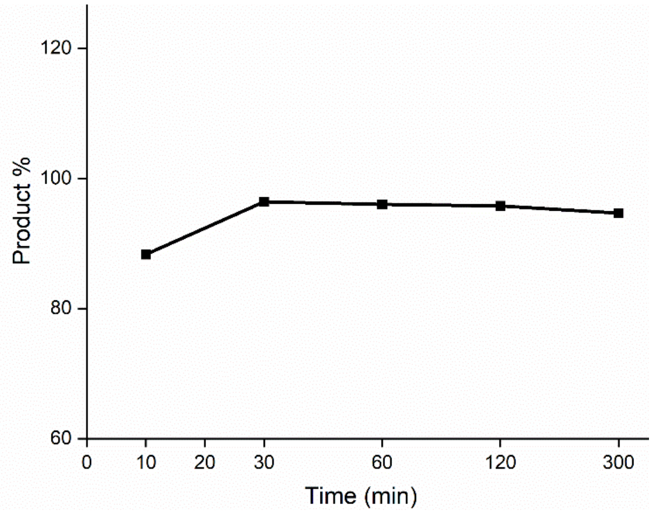

Figure S5. Rate of activation of 4-mers (-COOH). Mapped via the formation of amide bond between butylamine and 4-mer. The activation was mapped by the addition of butylamine through LC-MS.

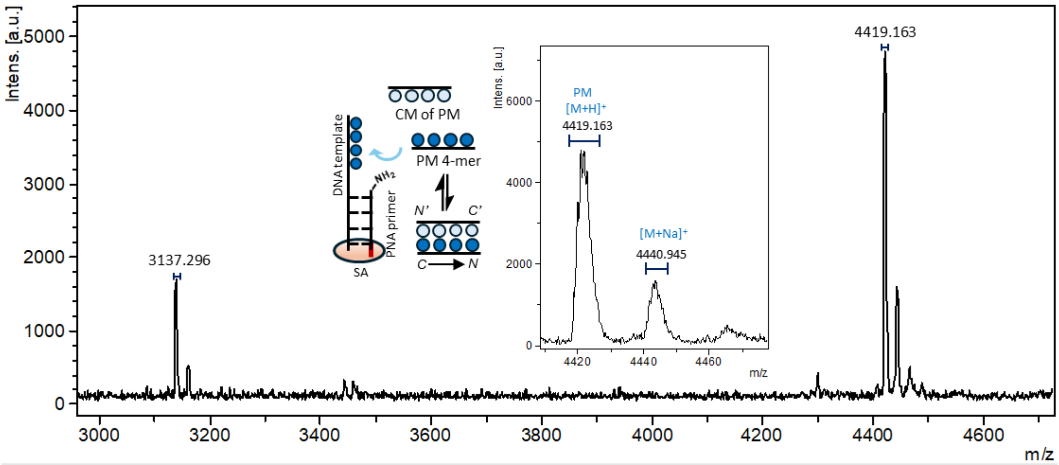

Figure S6. Primer extension in the presence of CM.

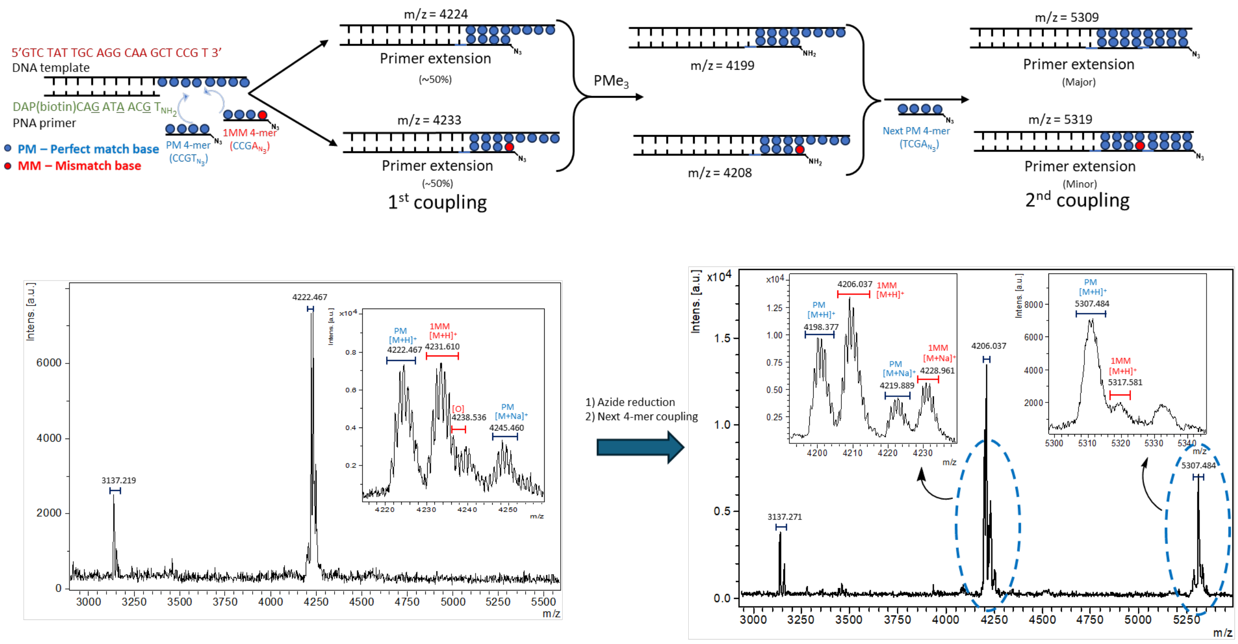

Figure S7: Primer extension with PM and 1MM followed by next coupling with Next PM.

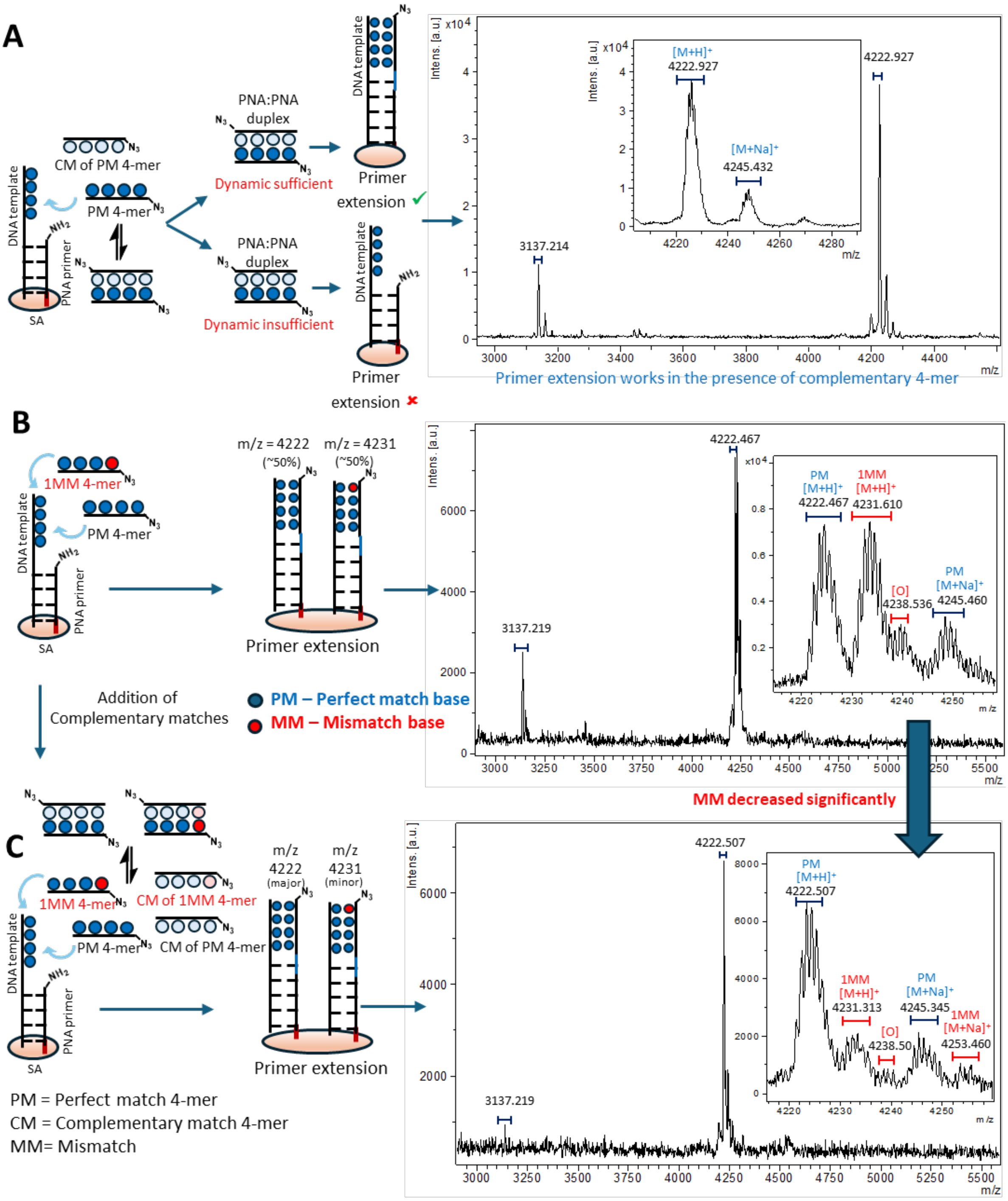
A
CM of PM 4-mer
PM 4-mer
DNA template
PNA primer
SA
PNA:PNA duplex
Dynamic sufficient
Dynamic insufficient
Primer extension
Primer extension works in the presence of complementary 4-mer
B
1MM 4-mer
PM 4-mer
m/z = 4222 (~50%)
m/z = 4231 (~50%)
Primer extension
Addition of Complementary matches
PM – Perfect match base
MM – Mismatch base
MM decreased significantly
C
1MM 4-mer
CM of 1MM 4-mer
PM 4-mer
CM of PM 4-mer
m/z 4222 (major)
m/z 4231 (minor)
Primer extension
PM = Perfect match 4-mer
CM = Complementary match 4-mer
MM= Mismatch

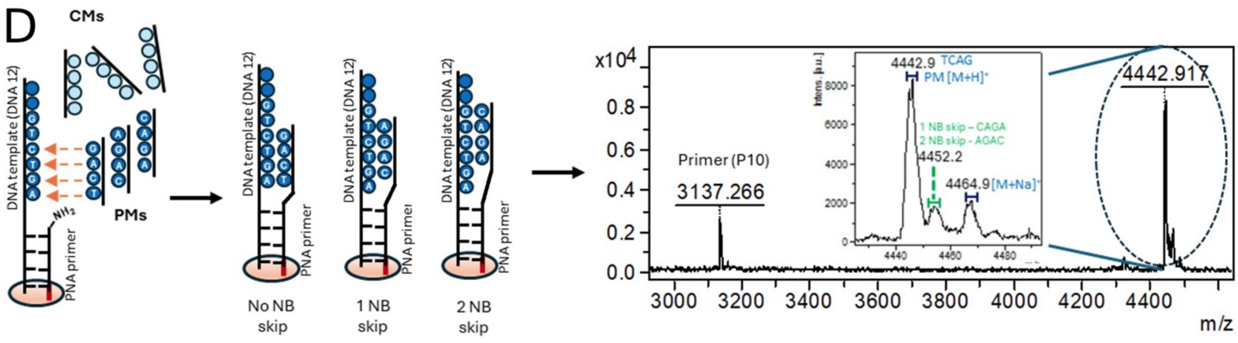

Figure S8: A) Templated primer extension with perfect match 4-mer in the presence of its complementary match that is in dynamic equilibrium with its duplex form. B) Templated reaction in the presence of perfect match and 1-mismatch without their complementary matches. C) Templated reaction in the presence of perfect match and 1-mismatch with their complementary matches (Mismatch peak significantly decreased). D_ Templated primer extension of DNA template (DNA 12), in presence of PM 4-mer, 1 and 2 nucleobase skipped 4-mers with their CMs in one pot. Primer extension with PM is high while with 1 and 2 NB skipped 4-mers, it's not significantly high. The 4-mers are Fmoc protected at the N-ter. 4-mer sequences are given in C→N way.

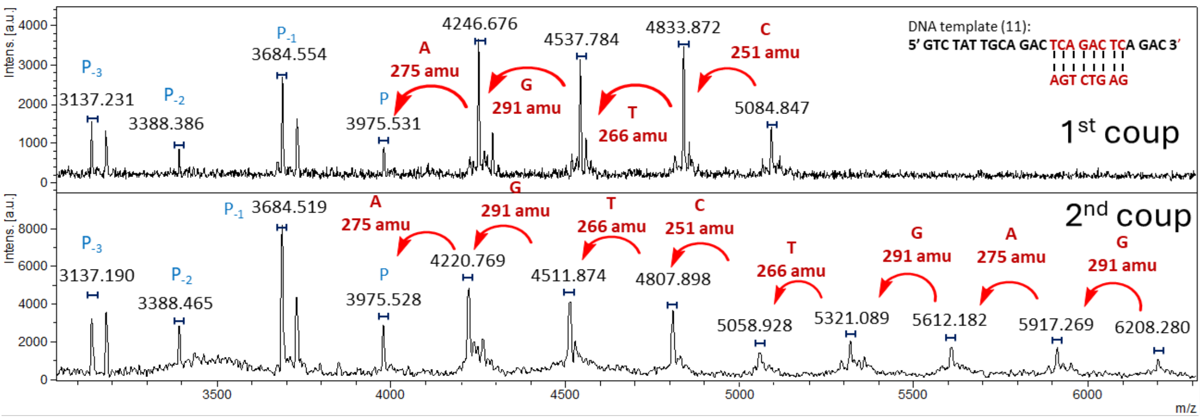

Figure S9: Templated reaction (2 couplings) with 64 mix 4-mers and DNA 11.

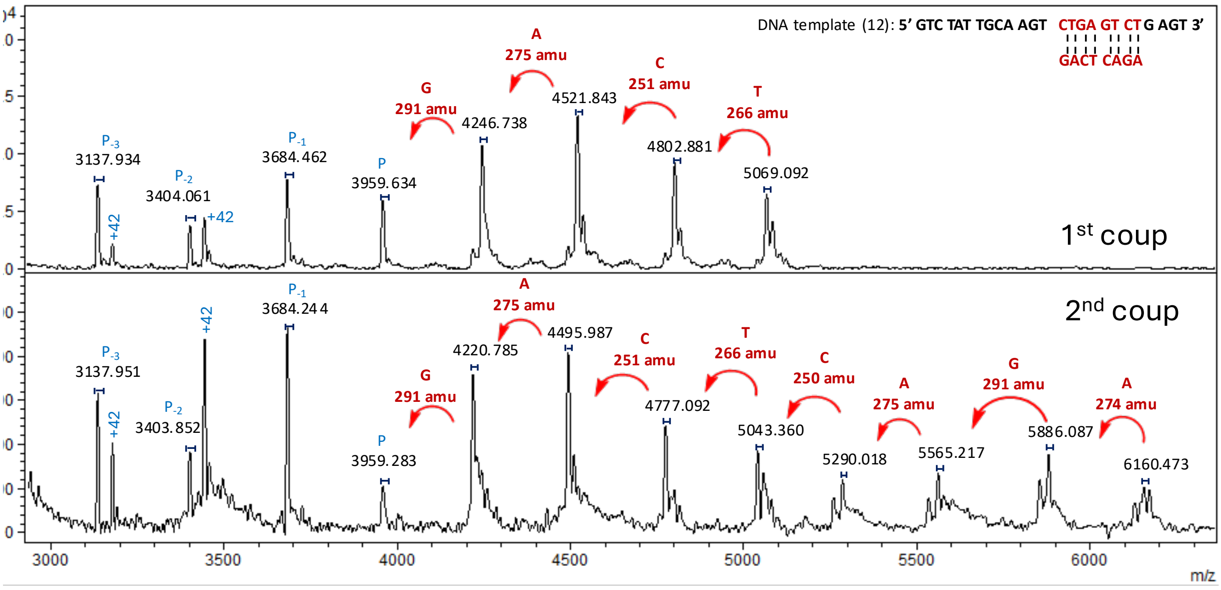

Figure S10: Templated reaction (2 couplings) with 64 mix 4-mers and DNA 12.

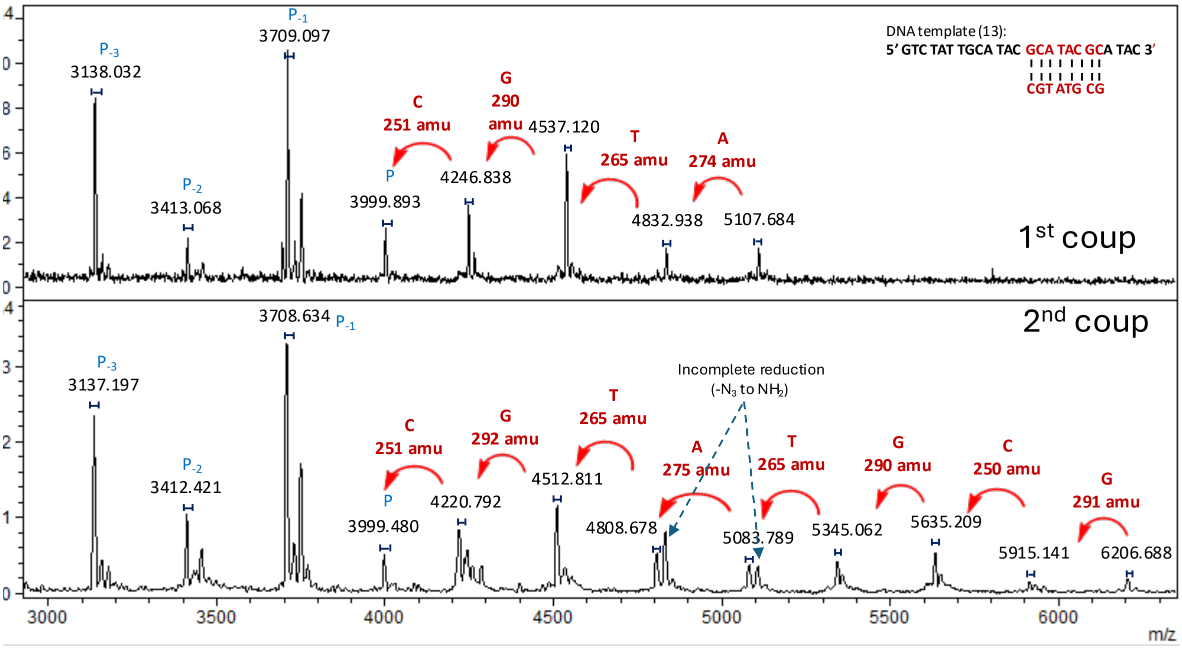

Figure S11: Templated reaction (2 couplings) with 64 mix 4-mers and DNA 13.

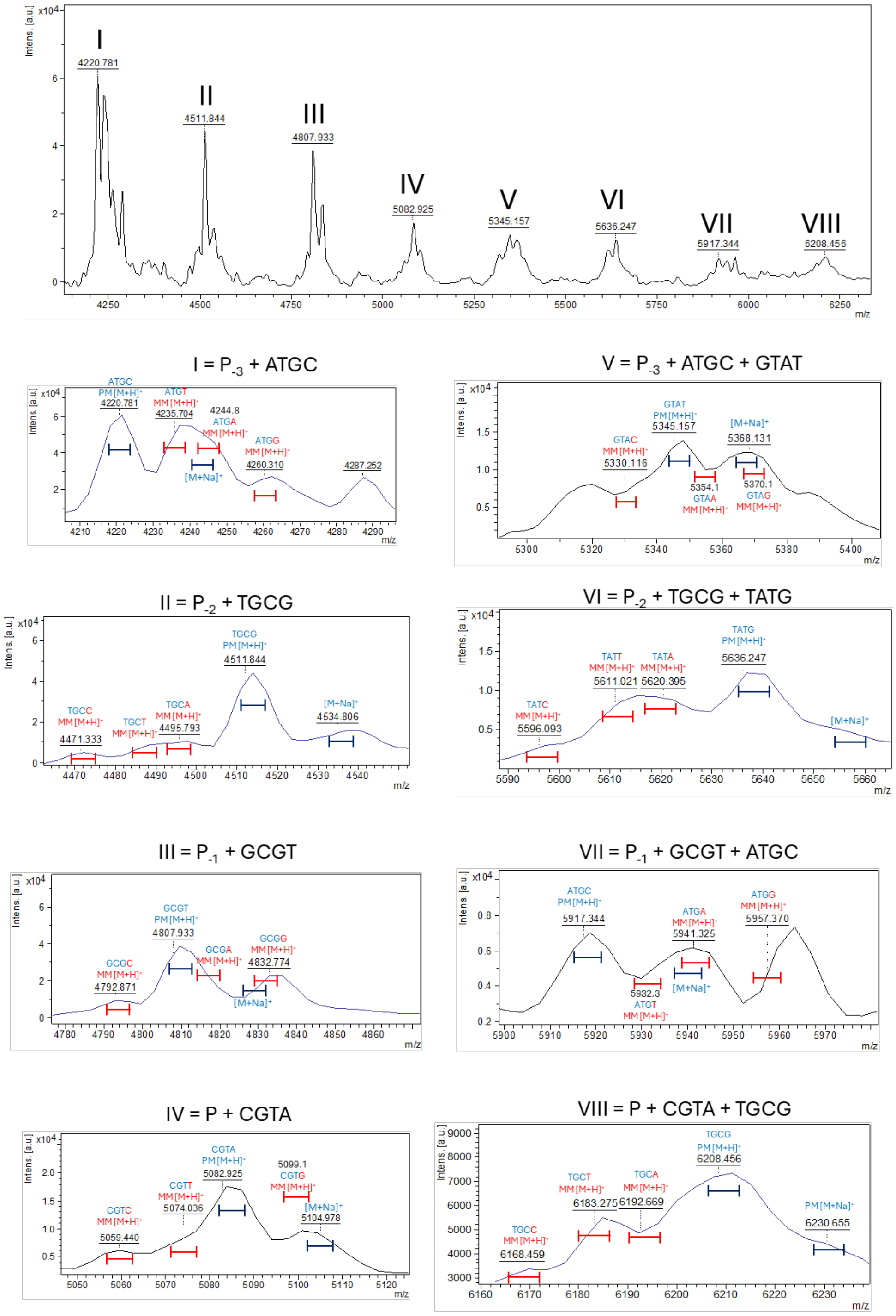

Figure S12: Zoom in view of the peaks of 256 mix 4-mers spectra (Figure 4B of manuscript) to show the potential terminal MMs along with Perfect match. Perfect match is the most intense peak as compared to MM.

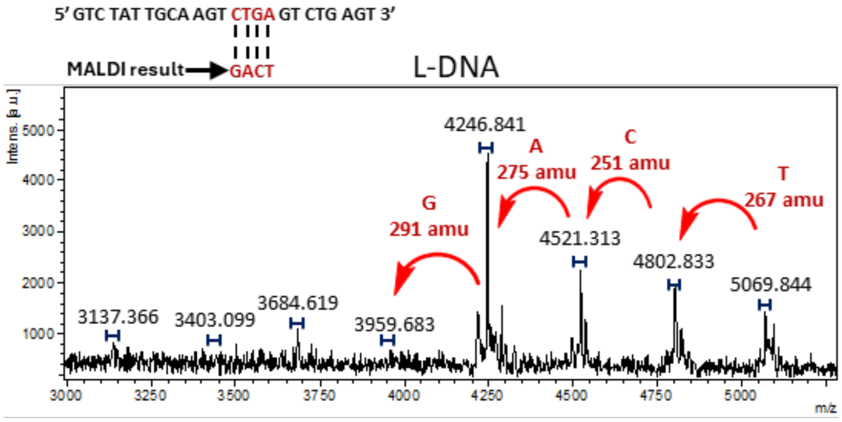

Figure S13: Primer extension of L-DNA template with 256 mix 4-mers.

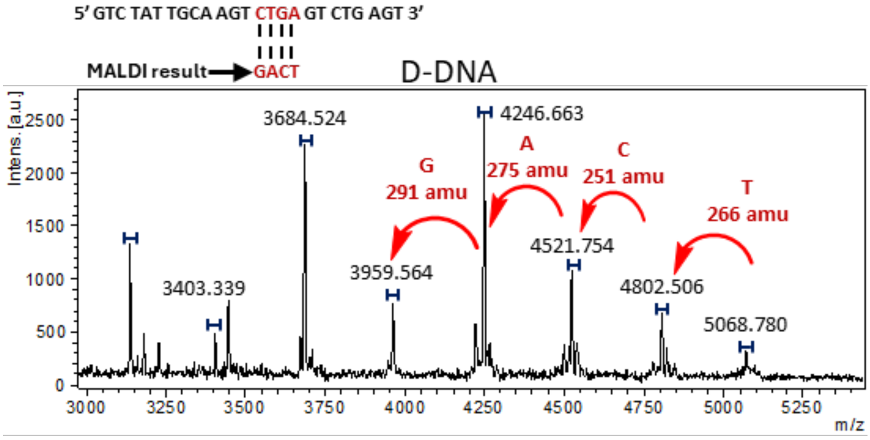

Figure S14: Primer extension of D-DNA template with 256 mix 4-mers.

Primers:

P10: Dap(biotin)CAG ATA ACG T

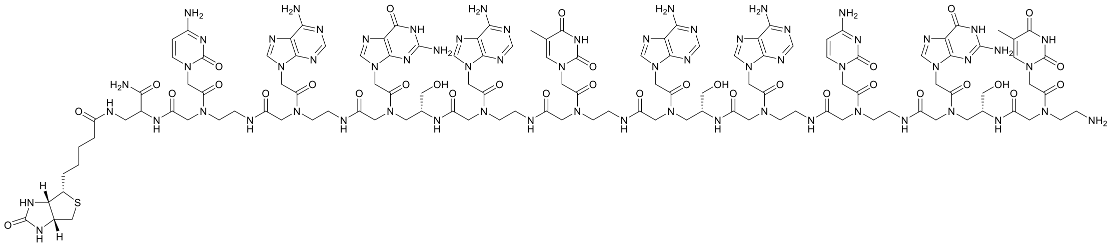

Chemical formula: $C_{124}H_{161}N_{65}O_{34}S$, MALDI-TOF-MS: m/z expected for $[M+H]^+$: 3137.266, m/z found: 3137.511

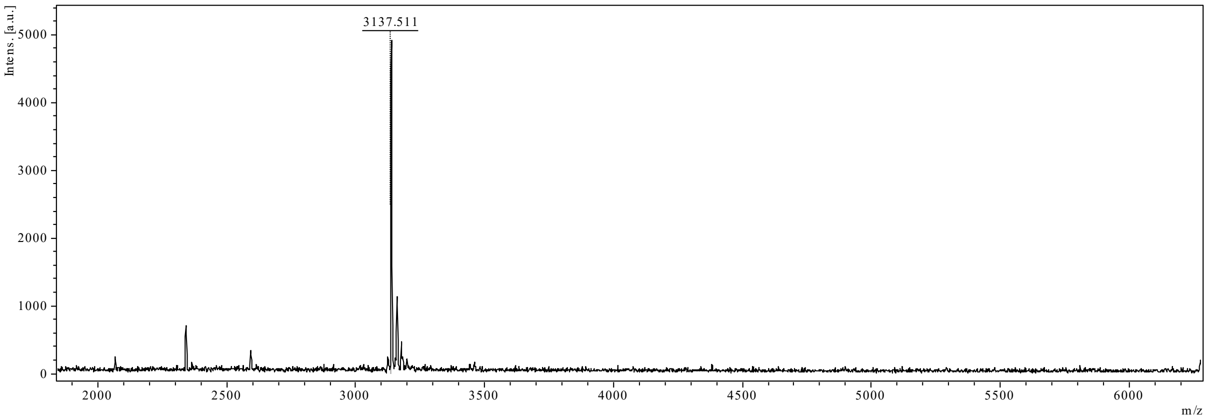

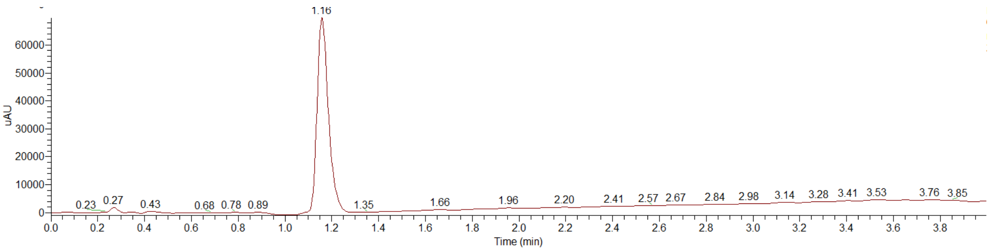

HRMS: expected for $[M+4H]^{4+}$: 785.3228, m/z found: 785.3166

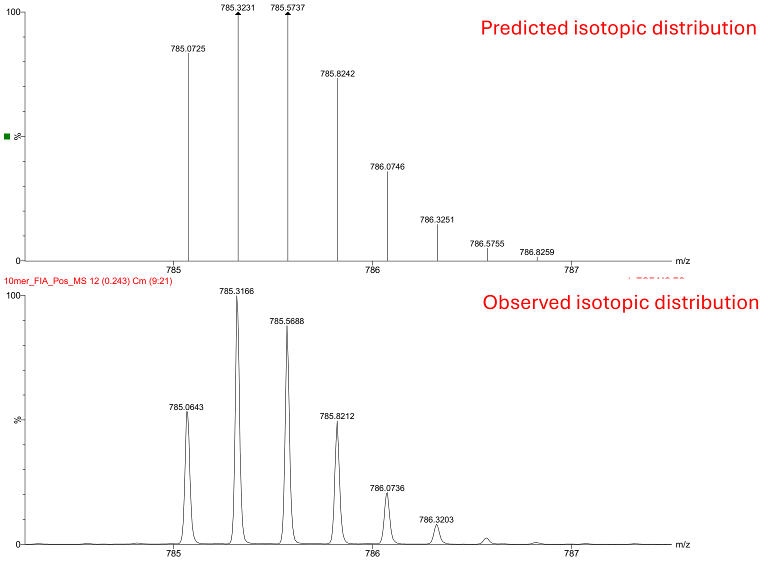

P11: Dap(Biotin)CAGATAACGTC

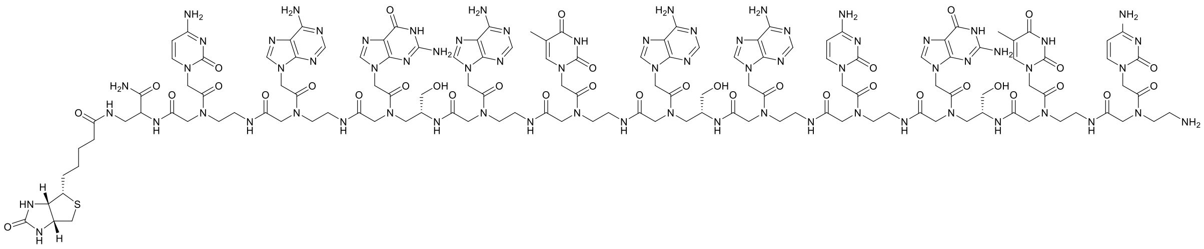

Chemical formula: $C_{135}H_{175}N_{70}O_{37}S$, MALDI-TOF-MS: m/z expected for $[M+H]^+$: 3388.3790, m/z found: 3388.368

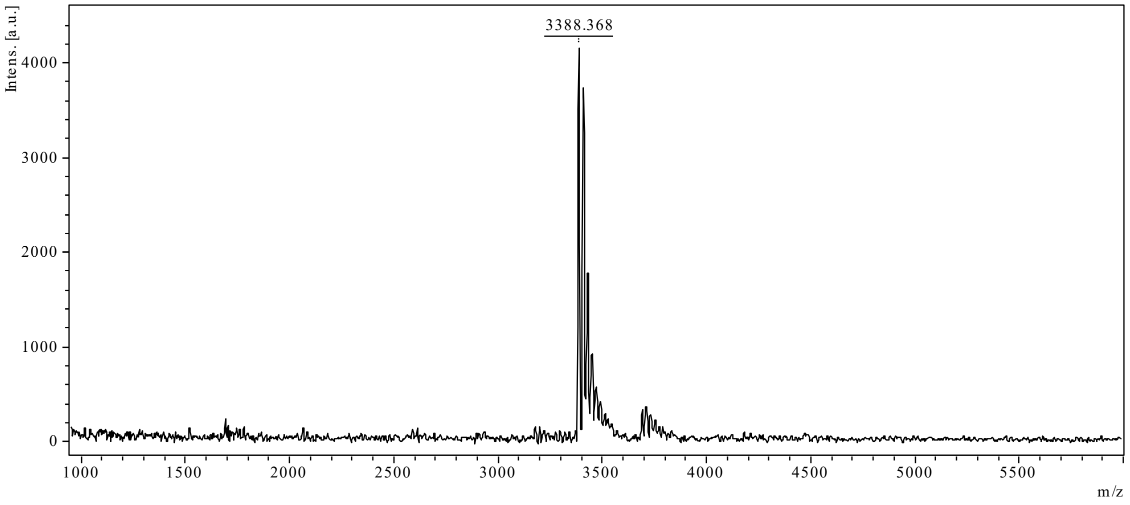

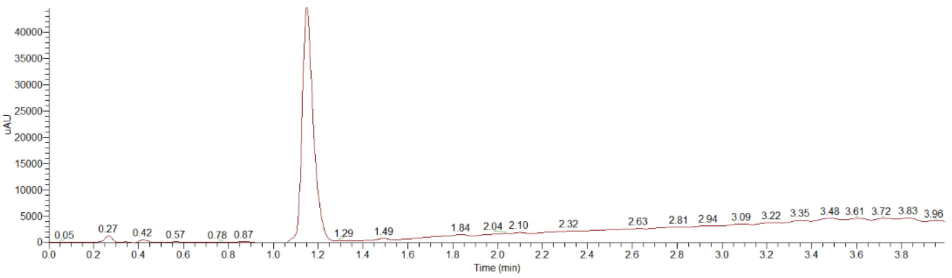

HRMS: expected for $[M+4H]^{4+}$: 848.0983, m/z found: 848.0930

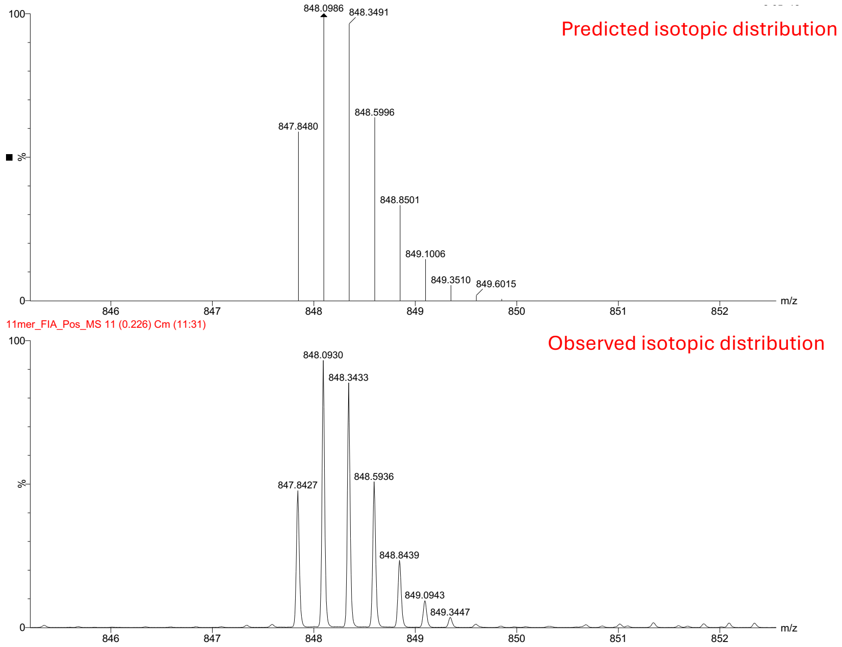

P12: Dap(Biotin)CAGATAACGTCC

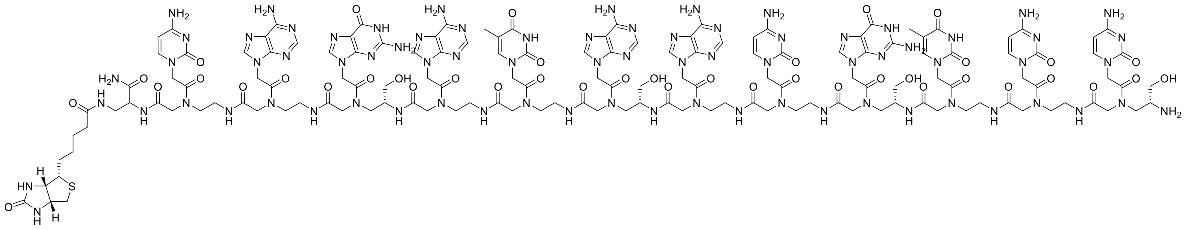

Chemical formula: $C_{145}H_{189}N_{75}O_{41}S$, MALDI-TOF-MS: m/z expected for $[M+H]^{+}$: 3669.6510, m/z found: 3669.480

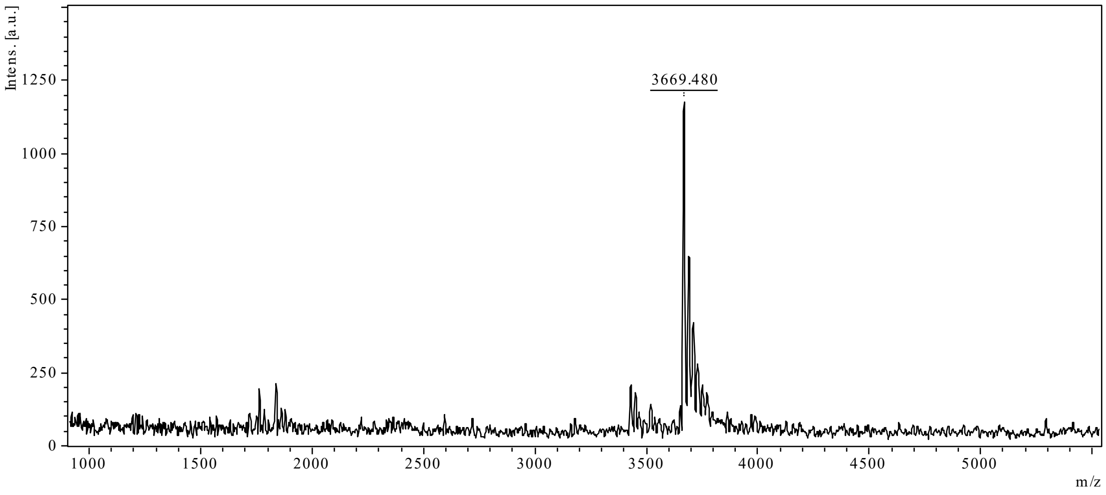

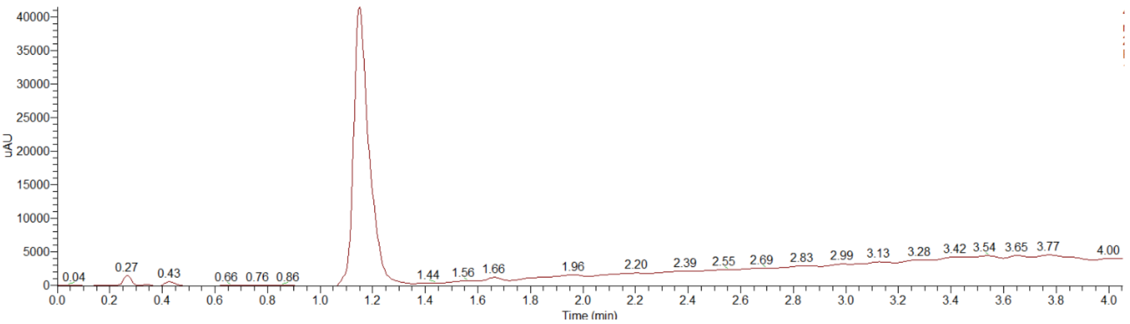

HRMS: expected for $[M+5H]^{5+}$: 734.9026, m/z found: 734.8984

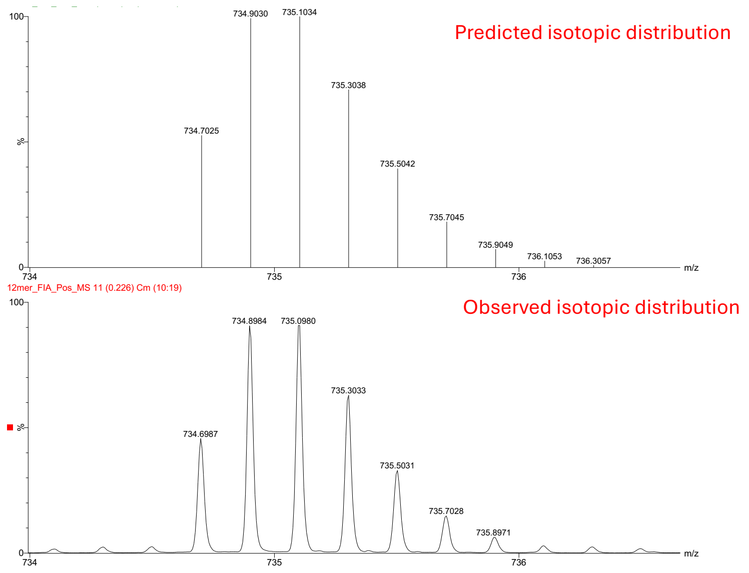

P13: Dap(Biotin)CA<u>G</u>AT<u>A</u>AC<u>G</u>TC<u>C</u>G

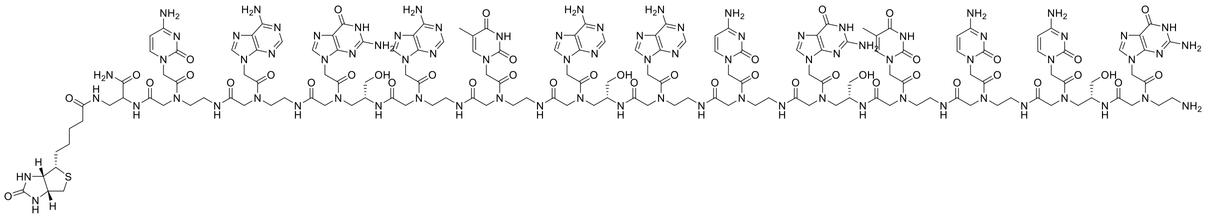

Chemical formula: $C_{156}H_{202}N_{82}O_{44}S$, MALDI-TOF-MS: m/z expected for $[M+H]^+$: 3960.5844, m/z found: 3960.588

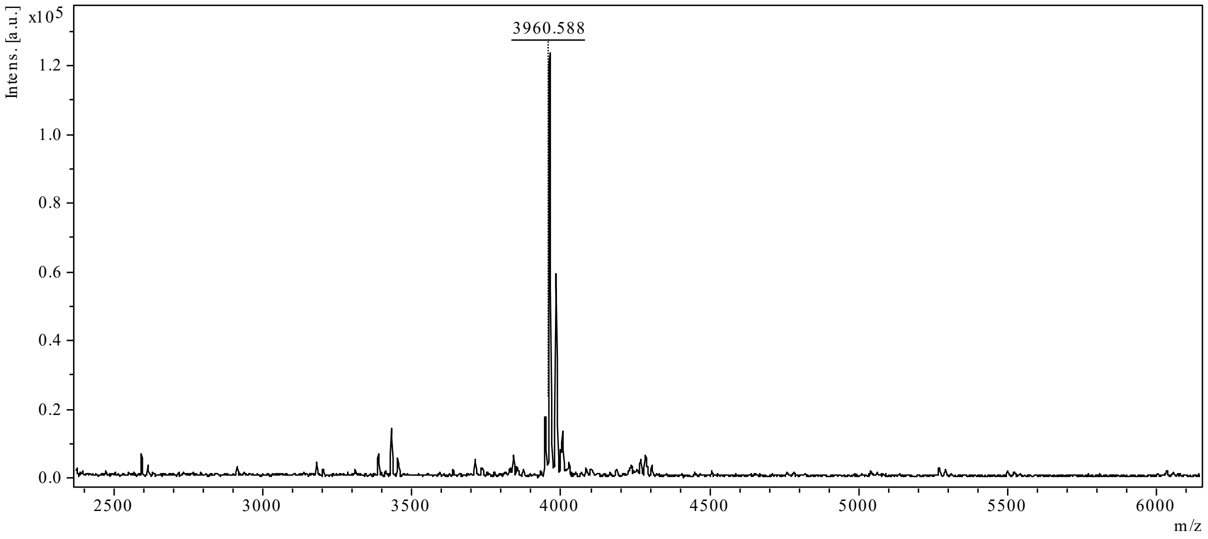

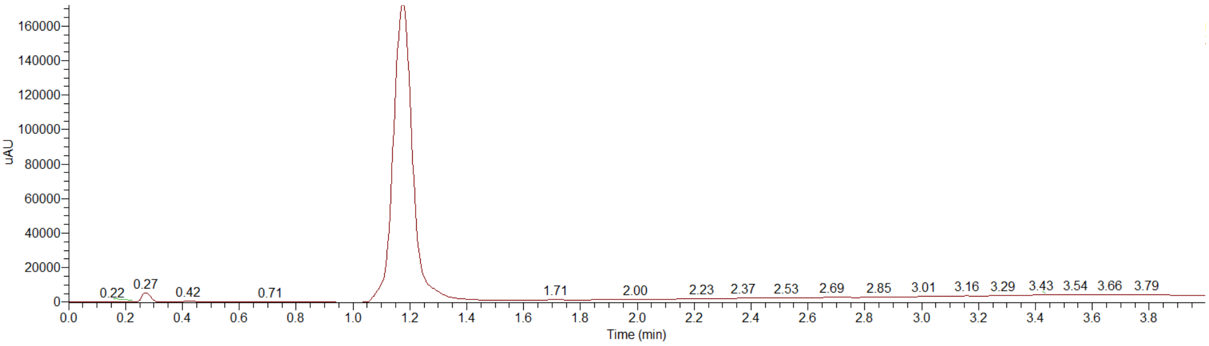

HRMS: expected for $[M+5H]^{5+}$: 793.1242, m/z found: 793.1224

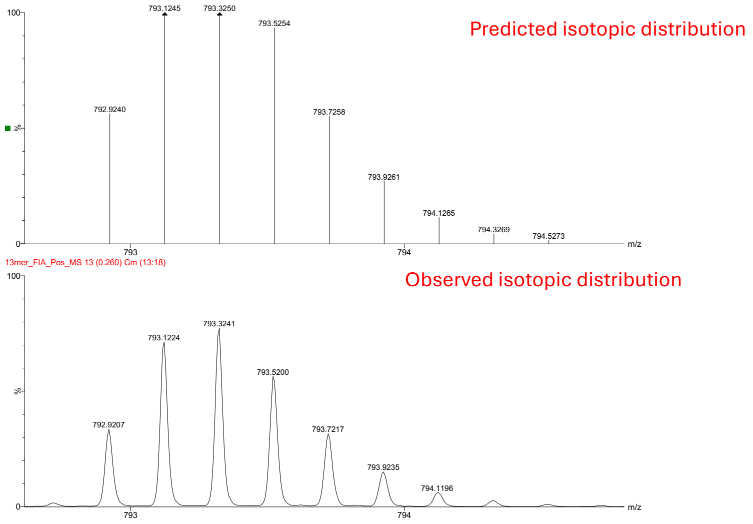

P14: Dap(Biotin)CAGATAACGTCCGT

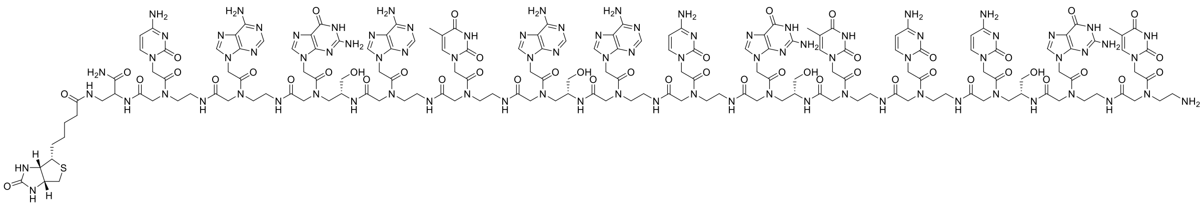

Chemical formula: $C_{167}H_{216}N_{86}O_{48}S$, MALDI-TOF-MS: m/z expected for $[M+H]^+$: 4226.6859, m/z found: 4226.690

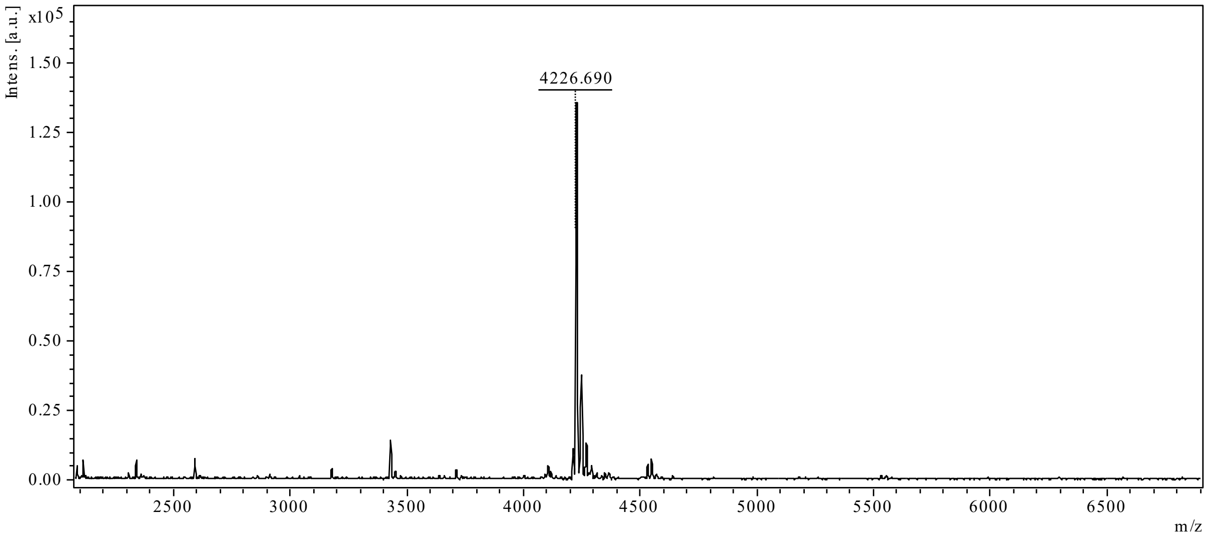

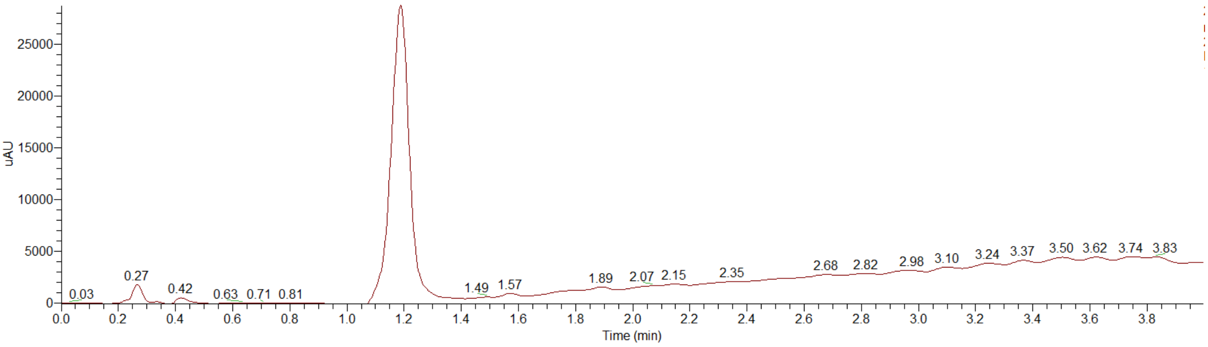

HRMS: expected for $[M+4H]^{4+}$: 1057.6788, m/z found: 1057.6733

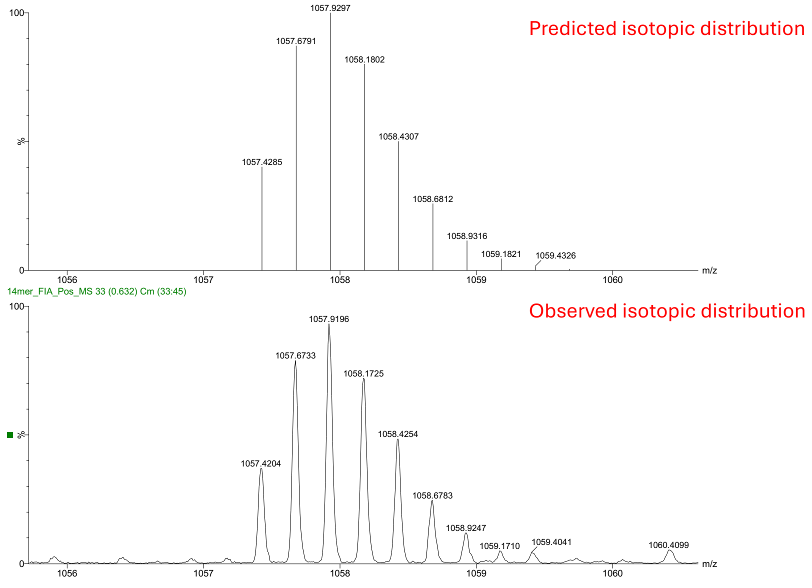

P15: DAP(biotin) CA<u>G</u> AT<u>A</u> AC<u>G</u> TC<u>T</u>

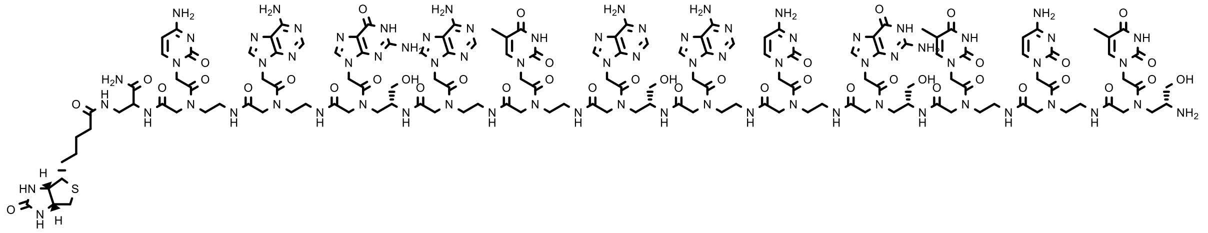

Chemical formula: $C_{146}H_{190}N_{74}O_{42}S$, MALDI-TOF-MS: m/z expected for $[M+H]^+$: 3684.4800, m/z found: 3685.588

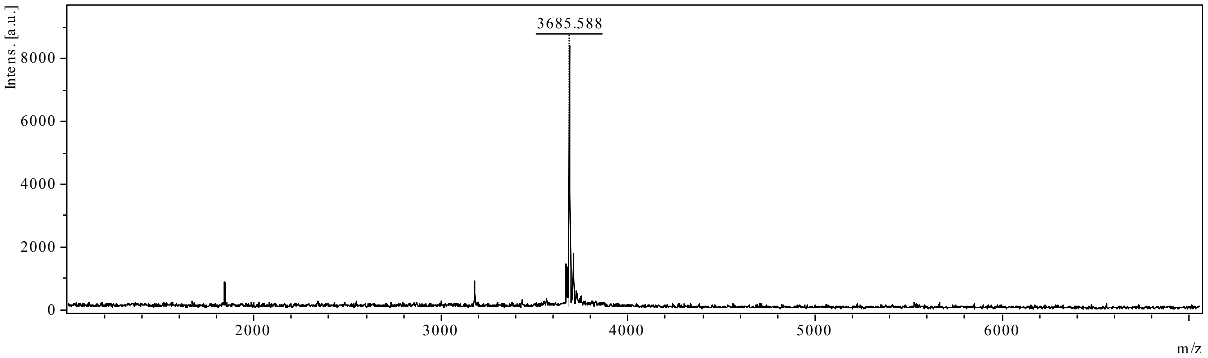

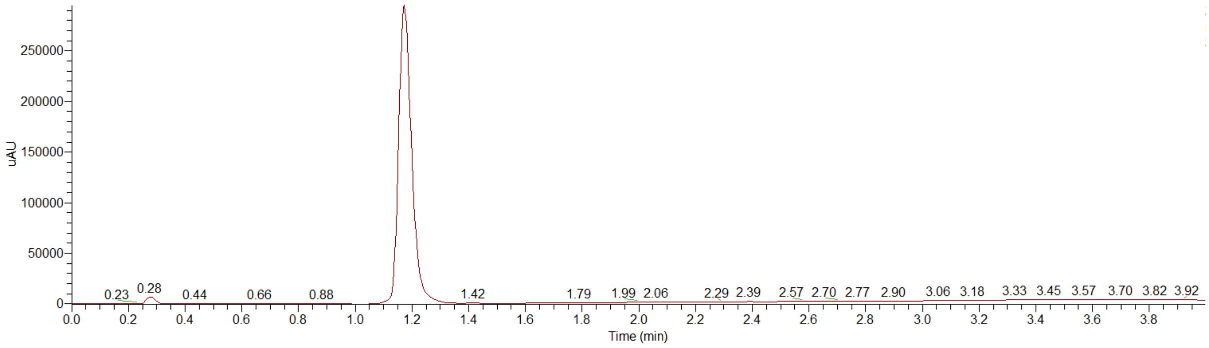

HRMS: expected for $[M+4H]^{4+}$: 922.1263, m/z found: 922.1216

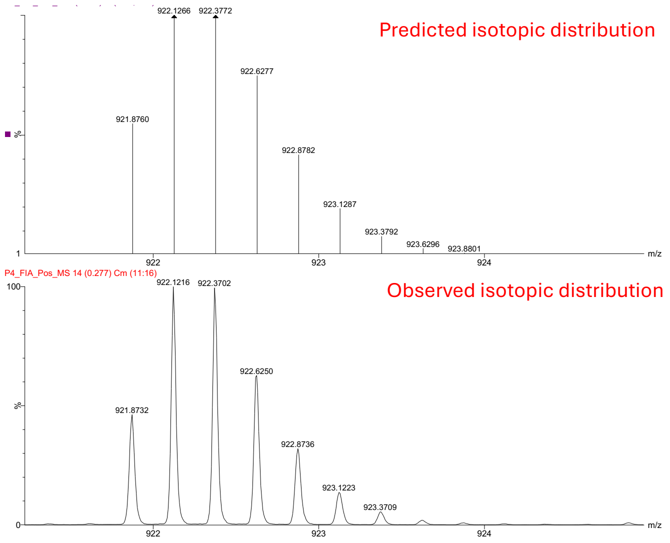

P16: DAP(biotin) CAG ATA ACG TCTG

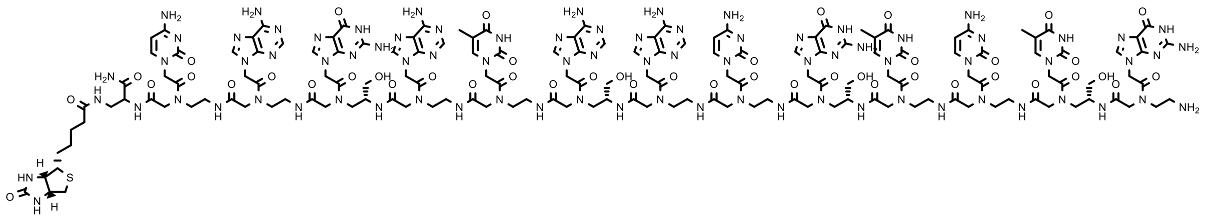

Chemical formula: $C_{157}H_{203}N_{81}O_{45}S$, MALDI-TOF-MS: m/z expected for $[M+H]^+$: 3975.5841, m/z found: 3977.713

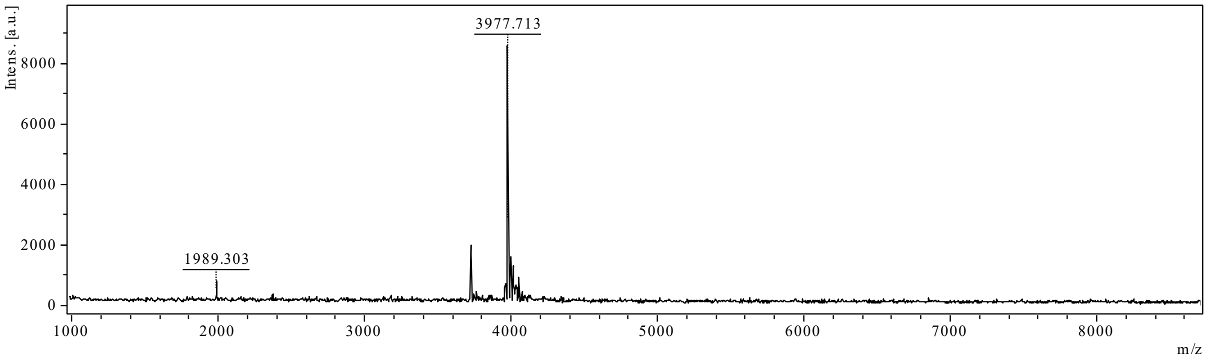

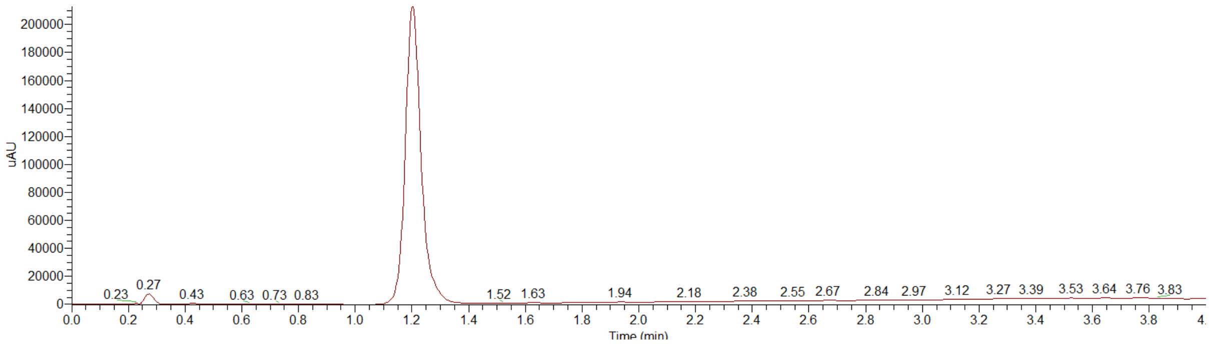

HRMS: expected for $[M+4H]^{4+}$: 994.9033, m/z found: 994.8929

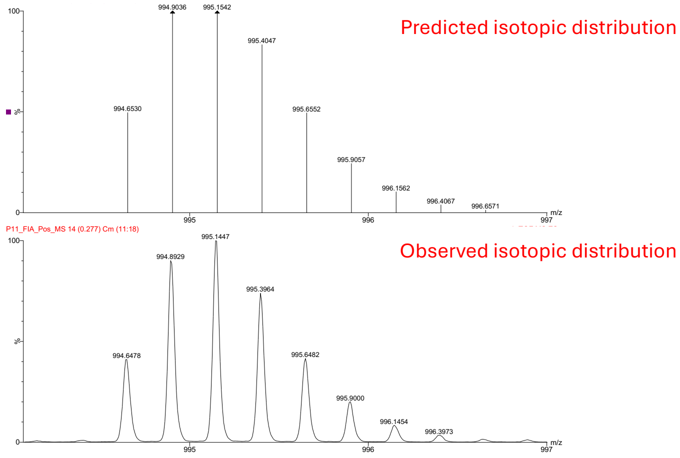

P17: DAP(Biotin) CAG ATA ACG TA

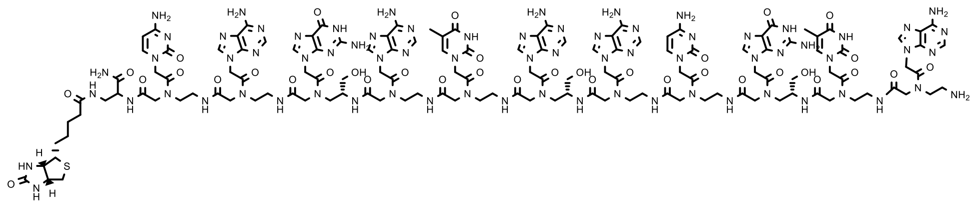

Chemical formula: $C_{135}H_{174}N_{72}O_{36}S$, MALDI-TOF-MS: m/z expected for $[M+H]^+$: 3412.3720, m/z found: 3413.460

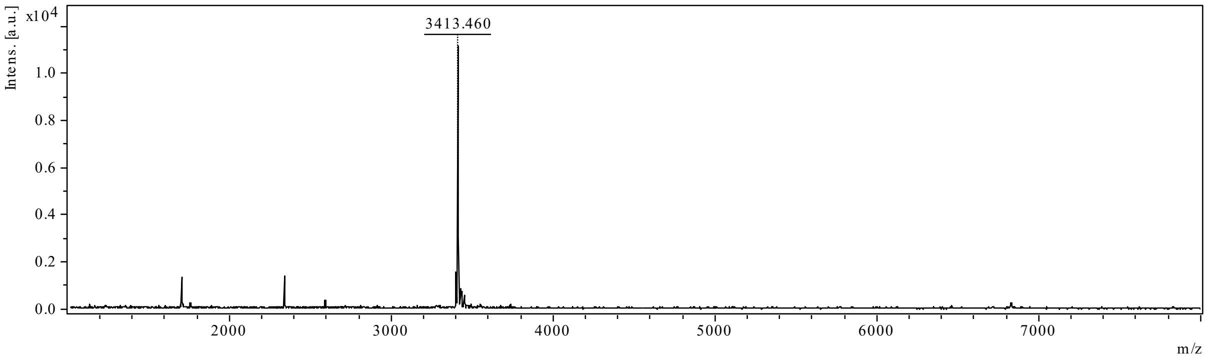

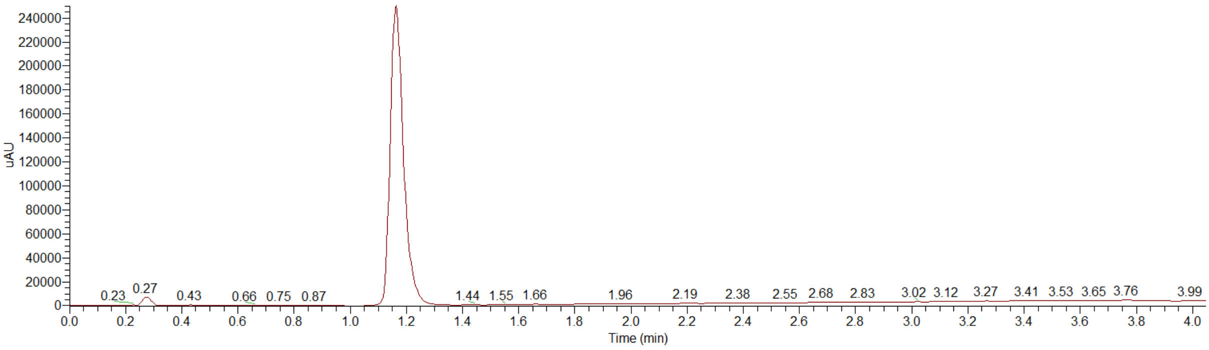

HRMS: expected for $[M+3H]^{3+}$: 1138.4657, m/z found: 1138.4579, $[M+2H]^{2+}$: 1707.1949, m/z found: 1707.1898

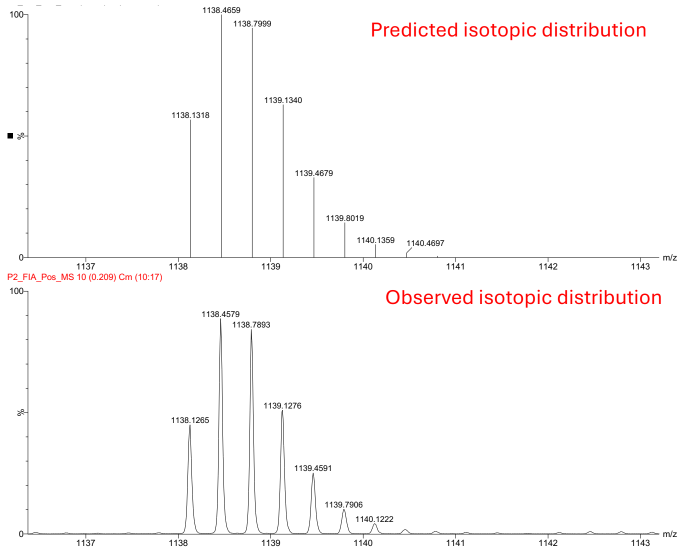

P18: DAP(Biotin) CA<u>G</u> AT<u>A</u> AC<u>G</u> TA<u>T</u>

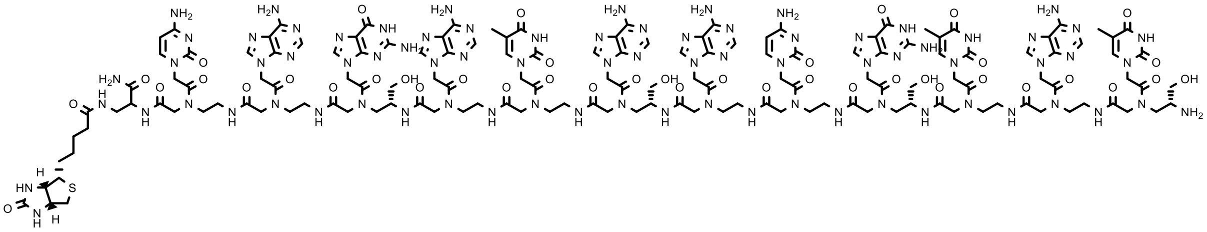

Chemical formula: $C_{147}H_{190}N_{76}O_{41}S$, MALDI-TOF-MS: m/z expected for $[M+H]^+$: 3708.4912, m/z found: 3709.825

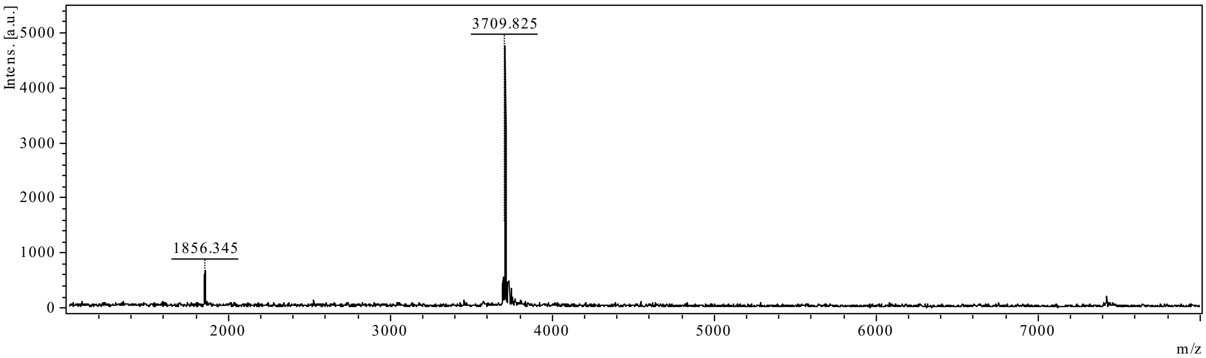

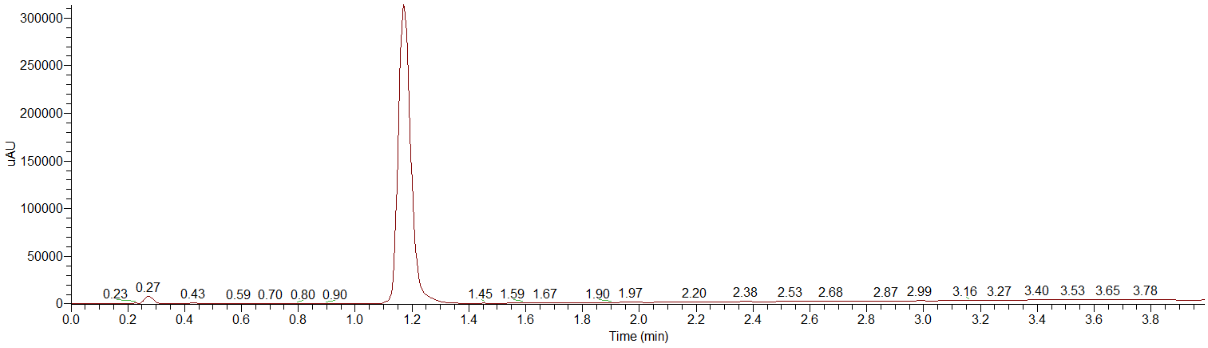

HRMS: expected for $[M+4H]^{4+}$: 928.1291, m/z found: 922.1209

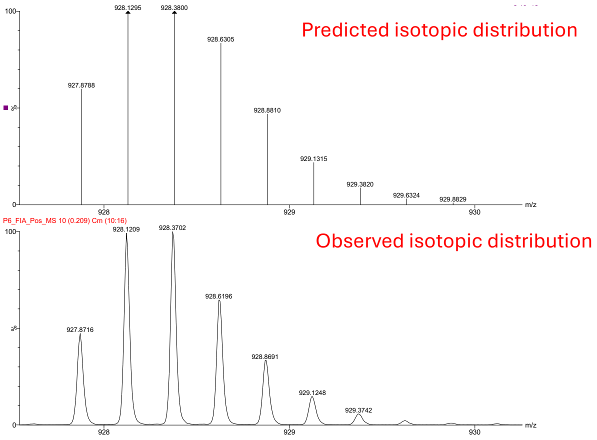

P19: DAP(biotin) CAG ATA ACG TATG

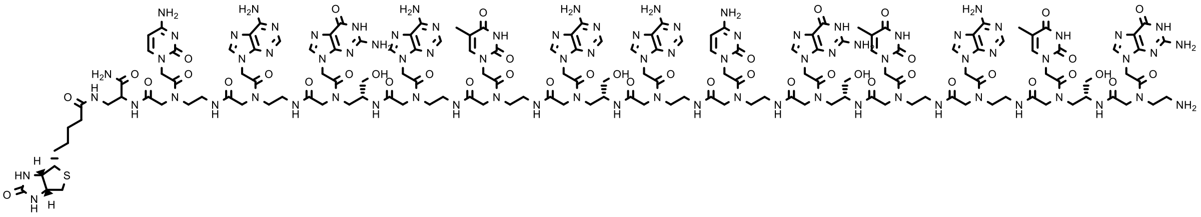

Chemical formula: $C_{158}H_{203}N_{83}O_{44}S$, MALDI-TOF-MS: m/z expected for $[M+H]^+$: 3999.5953, m/z found: 4002.066

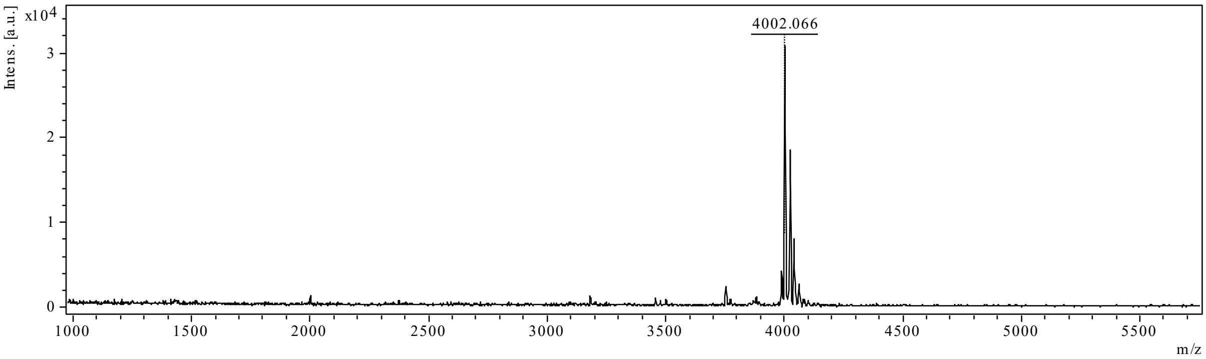

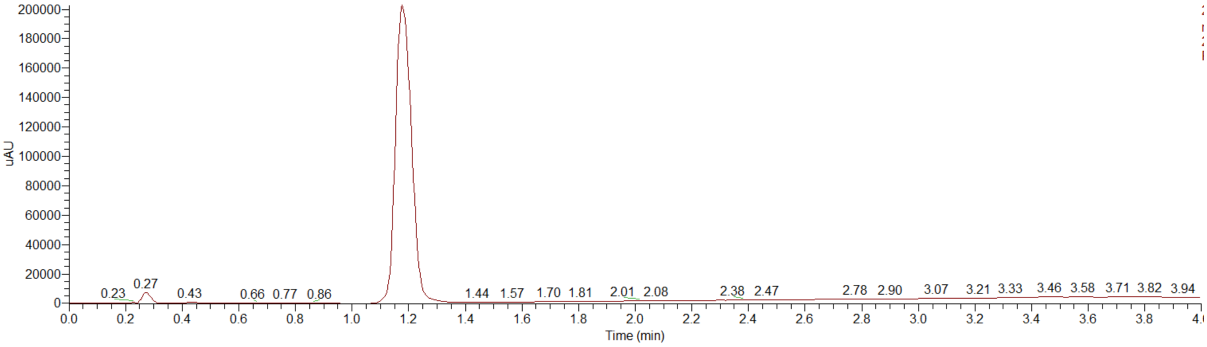

HRMS: expected for $[M+4H]^{4+}$: 1000.9061, m/z found: 1000.8975

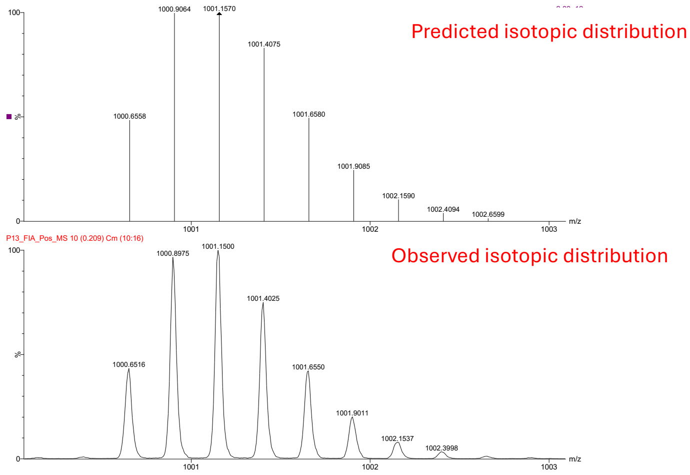

P20: DAP(biotin) CA<u>G</u> AT<u>A</u> AC<u>G</u> TT

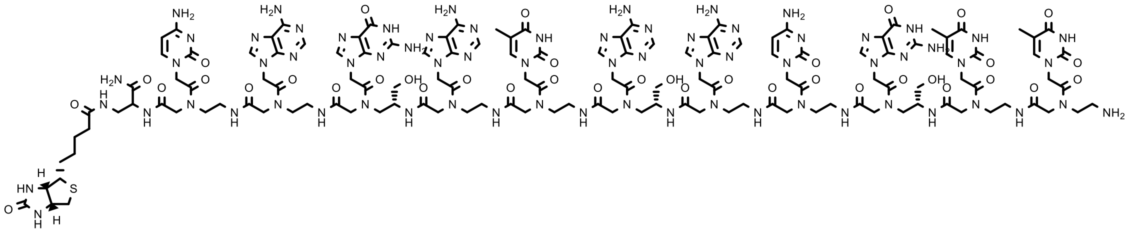

Chemical formula: $C_{135}H_{175}N_{69}O_{38}S$, MALDI-TOF-MS: m/z expected for $[M+H]^+$: 3403.3676, m/z found: 3403.967

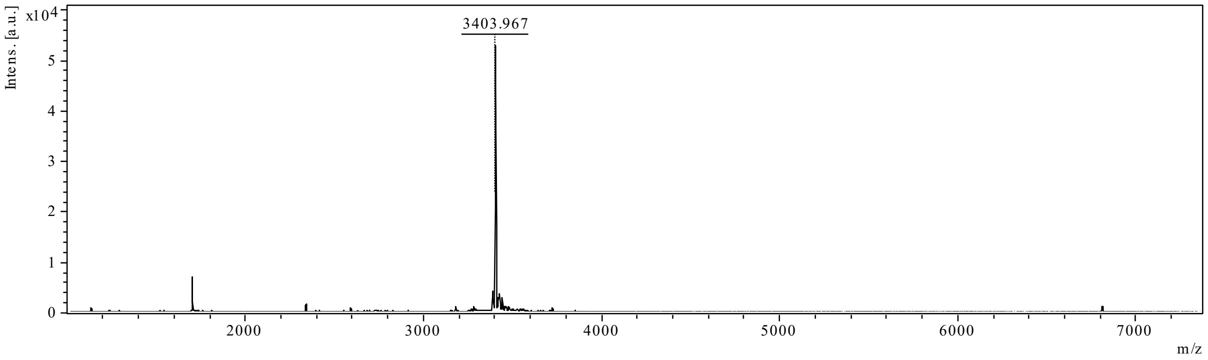

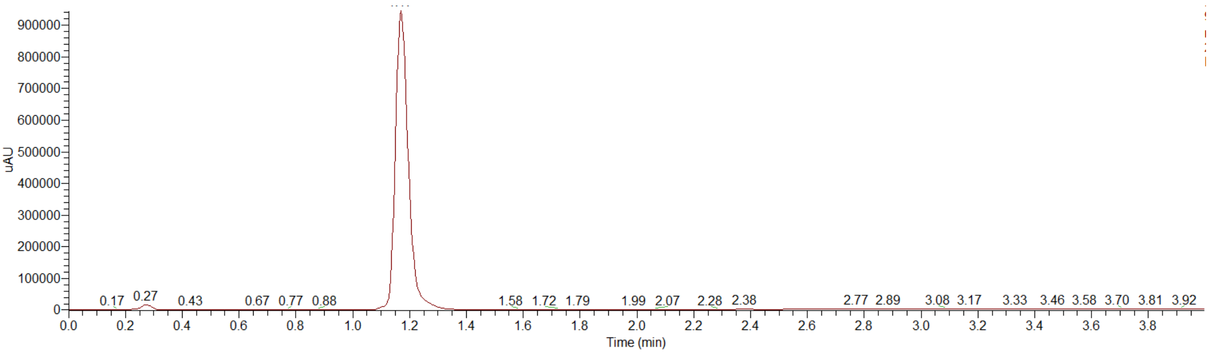

HRMS: expected for $[M+4H]^{4+}$: 851.8482, m/z found: 851.8450

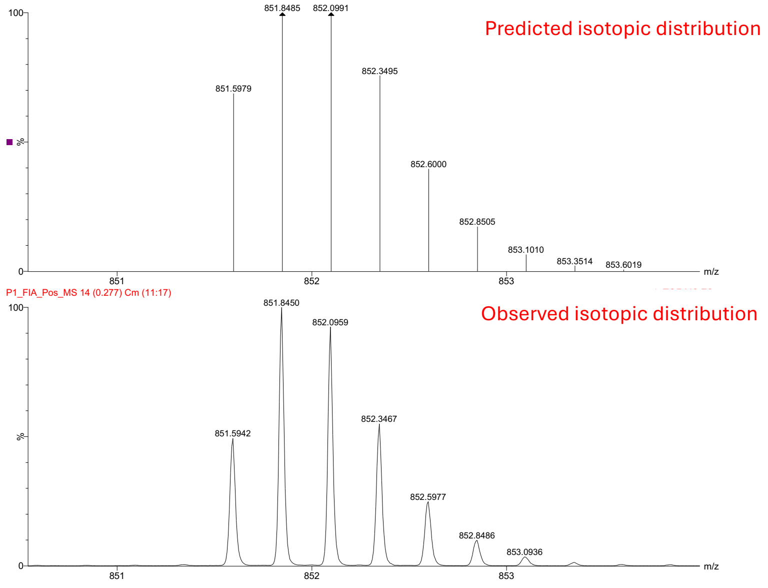

P21: DAP(biotin) CAG ATA ACG TTC

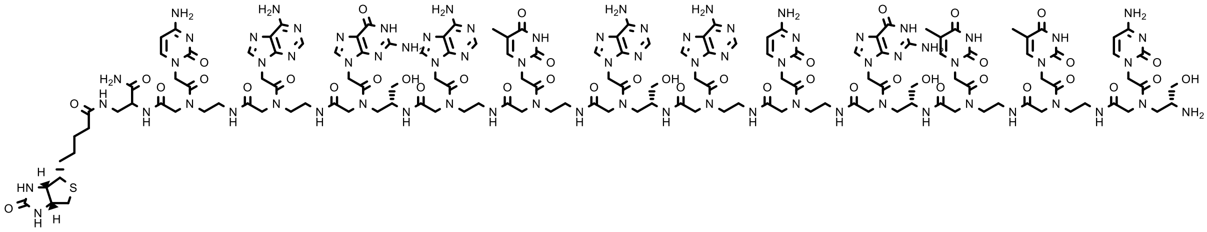

Chemical formula: $C_{146}H_{190}N_{74}O_{42}S$, MALDI-TOF-MS: m/z expected for $[M+H]^+$: 3684.4800, m/z found: 3686.009

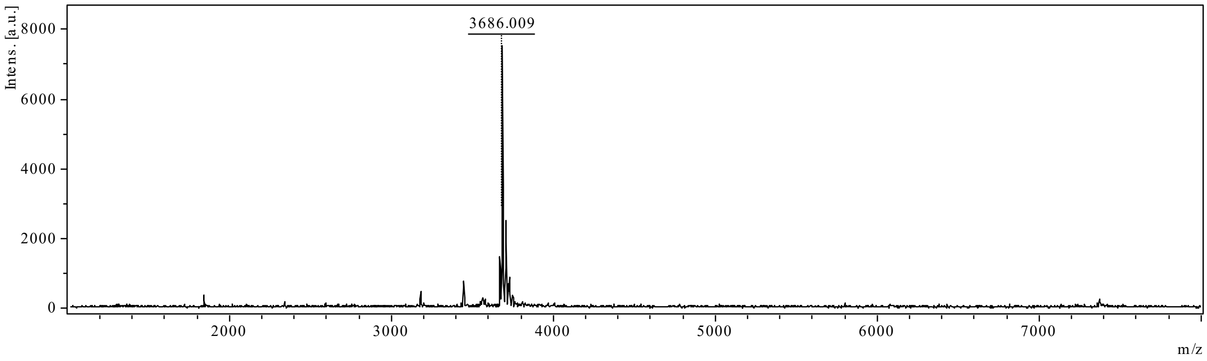

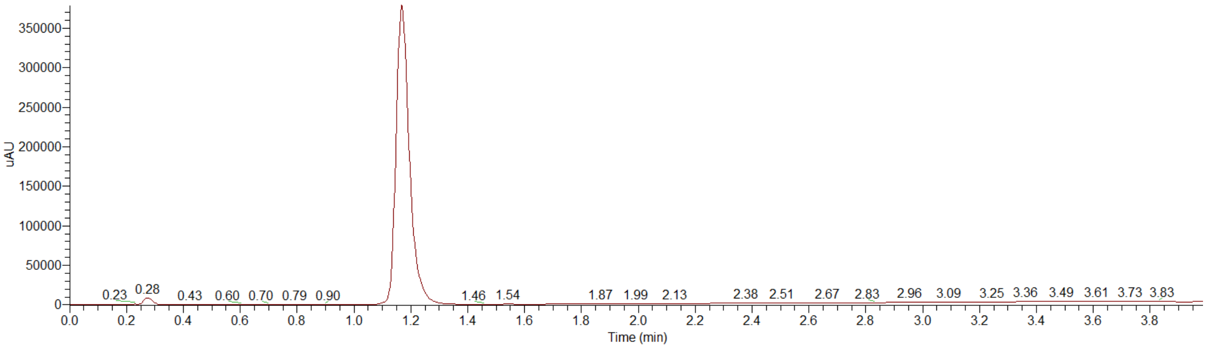

HRMS: expected for $[M+4H]^{4+}$: 922.1263, m/z found: 922.1216

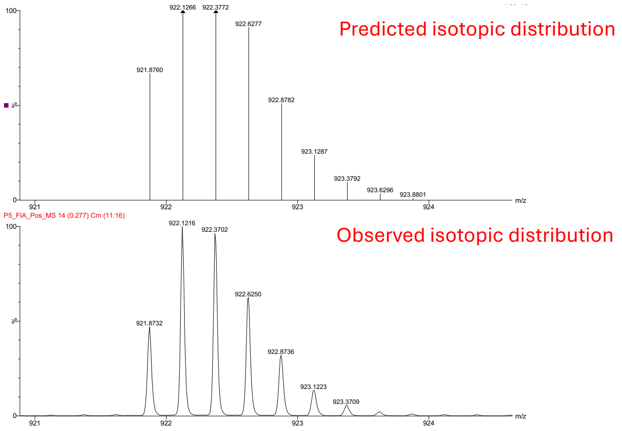

P22: DAP(biotin) CAG ATA ACG TTCA

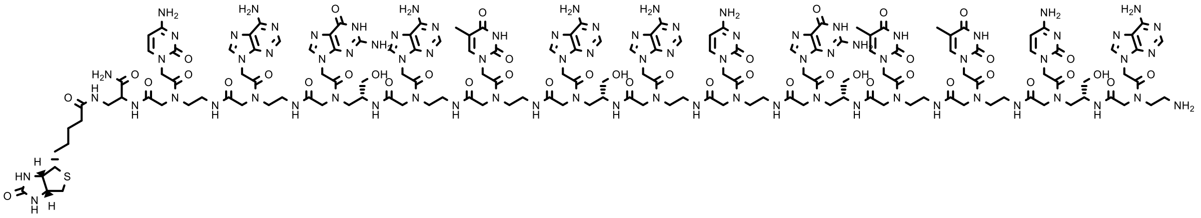

Chemical formula: $C_{157}H_{203}N_{81}O_{44}S$, MALDI-TOF-MS: m/z expected for $[M+H]^+$: 3959.5931, m/z found: 3962.043

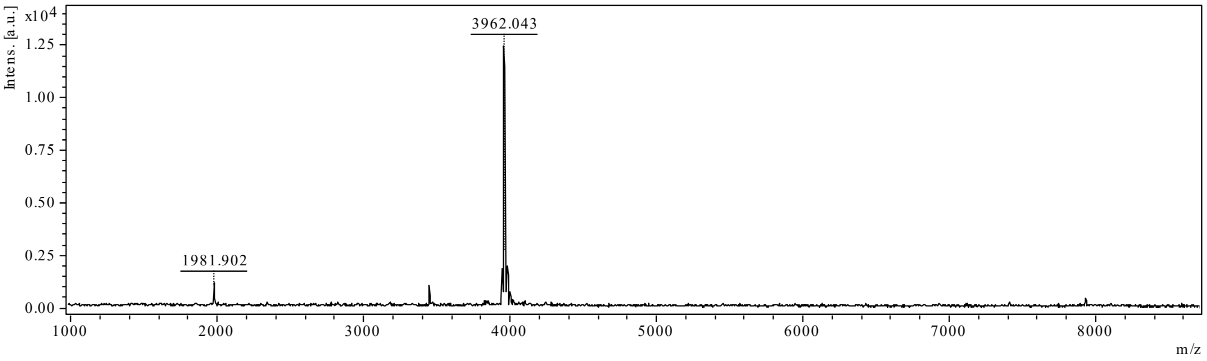

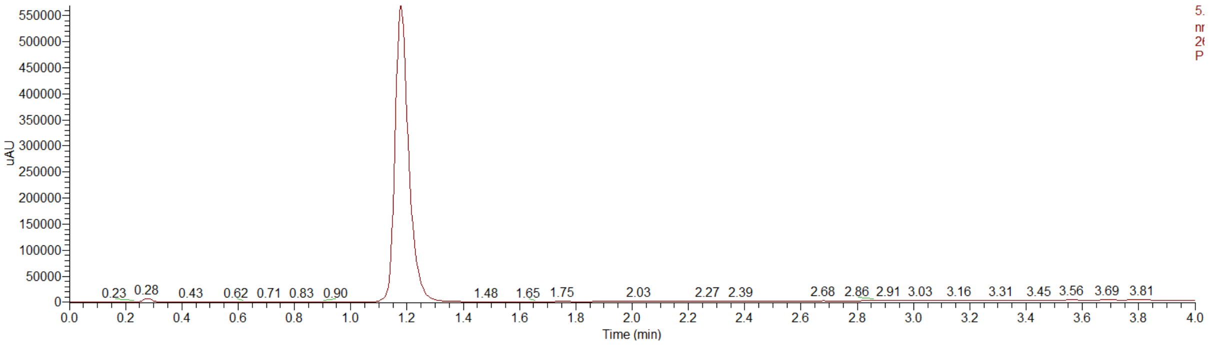

HRMS: expected for $[M+3H]^{3+}$: 1320.8703, m/z found: 1320.8678

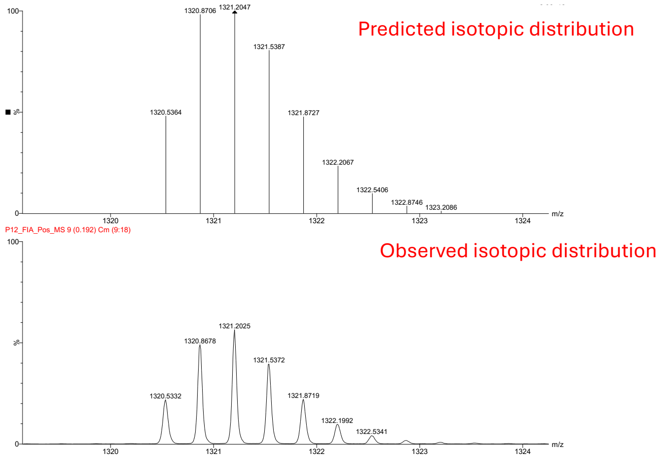

10mer_L (P10’) - Biotin-DAP- CAG ATA ACG T

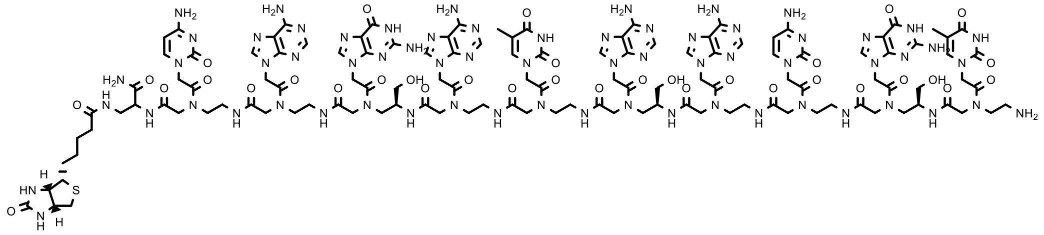

Chemical formula: $C_{124}H_{161}N_{65}O_{34}S$, MALDI-TOF-MS: m/z expected for $[M+H]^+$: 3137.2661, m/z found: 3137.628

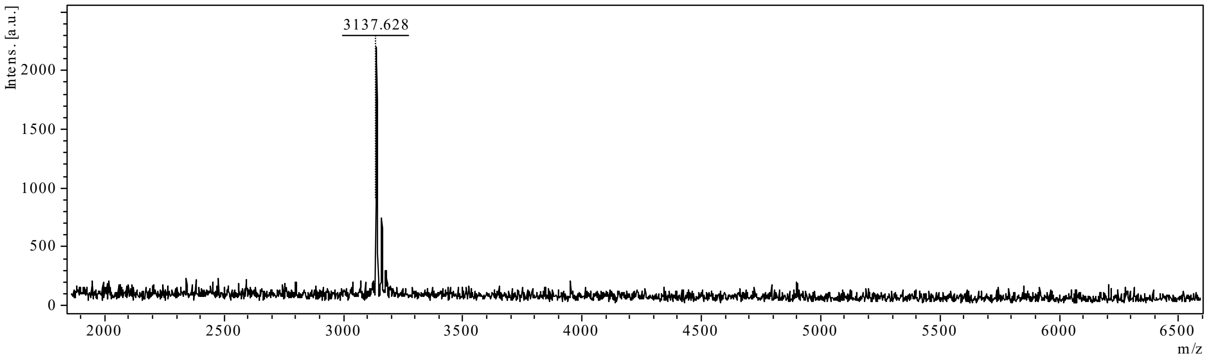

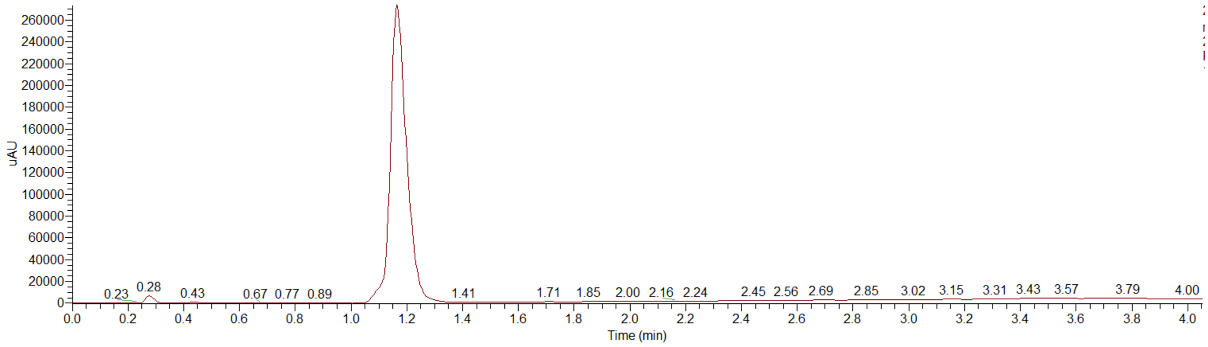

HRMS: expected for $[M+4H]^{4+}$: 785.3228, m/z found: 785.3166

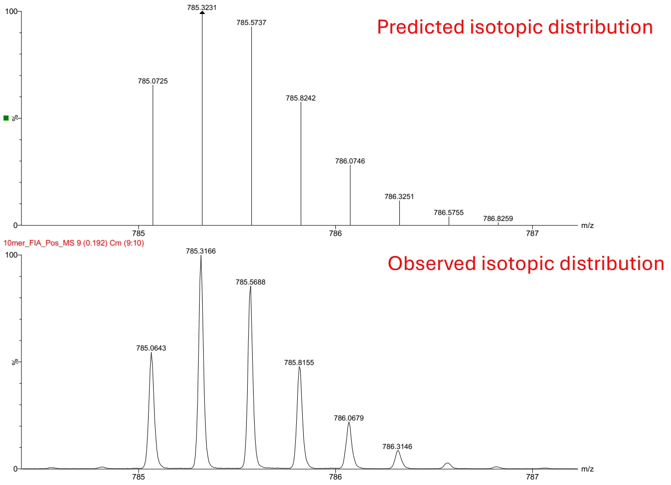

11mer_L (P20') - Biotin-DAP- CA<u>G</u> AT<u>A</u> AC<u>G</u> TT

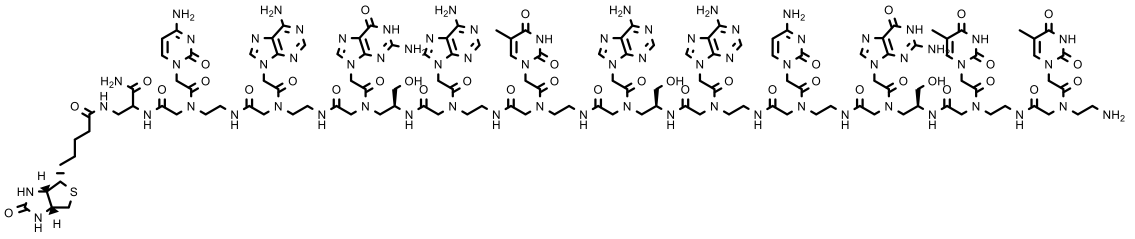

Chemical formula: $C_{135}H_{175}N_{69}O_{38}S$, MALDI-TOF-MS: m/z expected for $[M+H]^+$: 3403.3676, m/z found: 3403.876

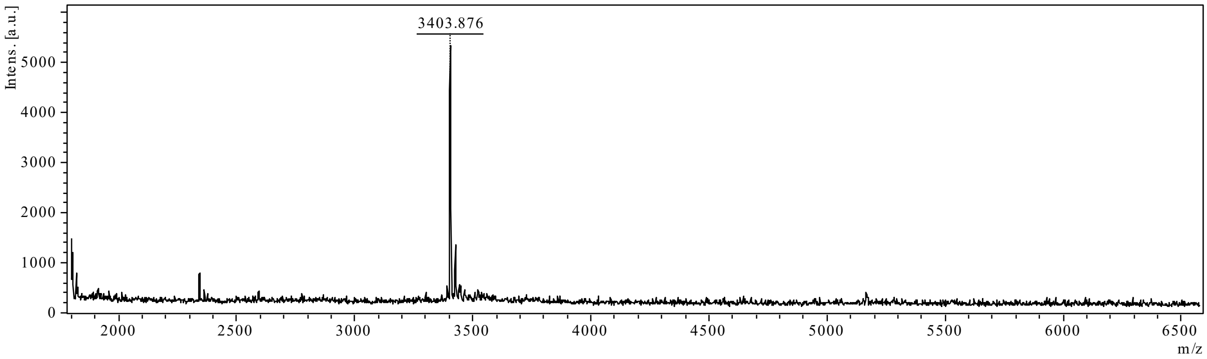

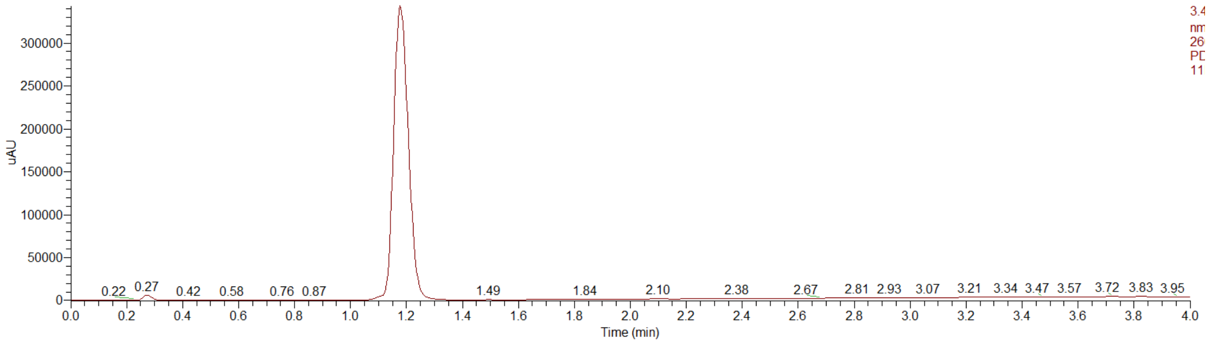

HRMS: expected for $[M+4H]^{4+}$: 851.8482, m/z found: 851.8391

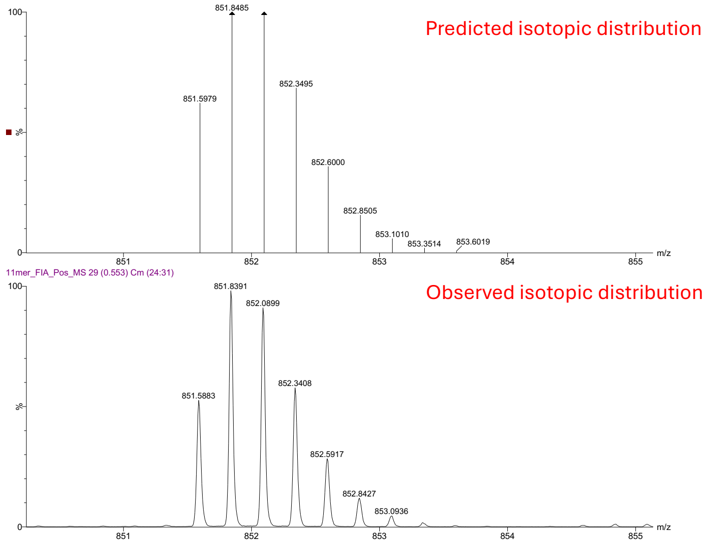

12 mer_L (P21’) - Biotin-DAP- CA<u>G</u> AT<u>A</u> AC<u>G</u> TT<u>C</u>

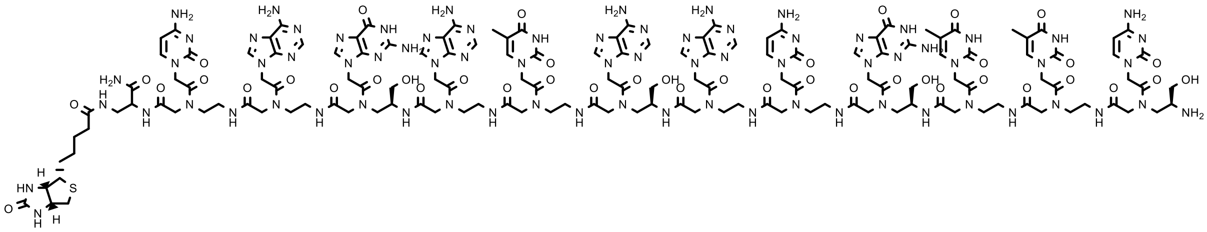

Chemical formula: $C_{146}H_{190}N_{74}O_{42}S$, MALDI-TOF-MS: m/z expected for $[M+H]^{+}$: 3684.4800, m/z found: 3685.029

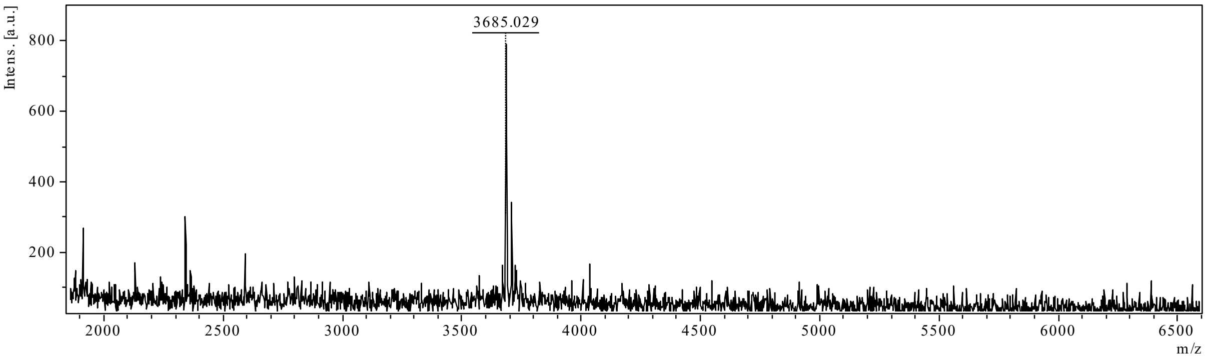

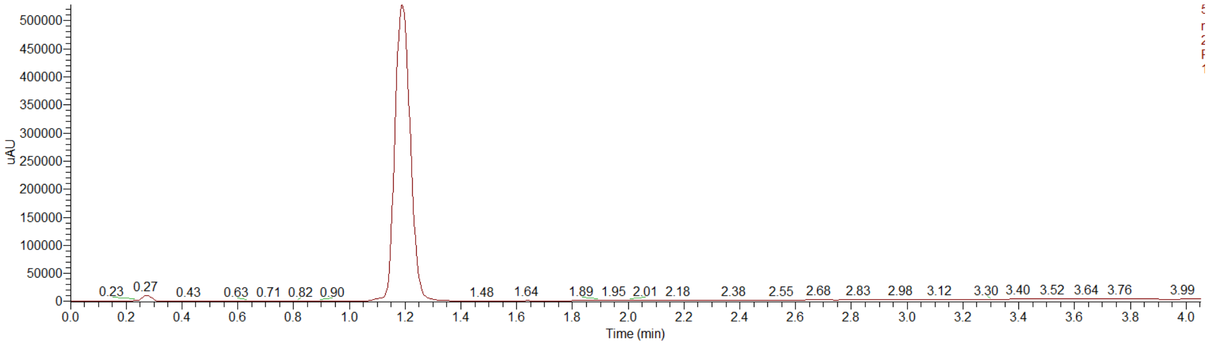

HRMS: expected for $[M+4H]^{4+}$: 922.1263, m/z found: 922.1216

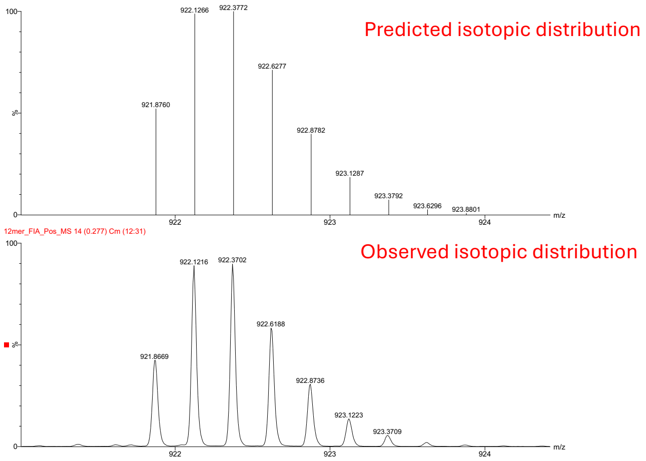

13mer_L (P22’) - Biotin-DAP- CA<u>G</u> AT<u>A</u> AC<u>G</u> TT<u>C</u>A

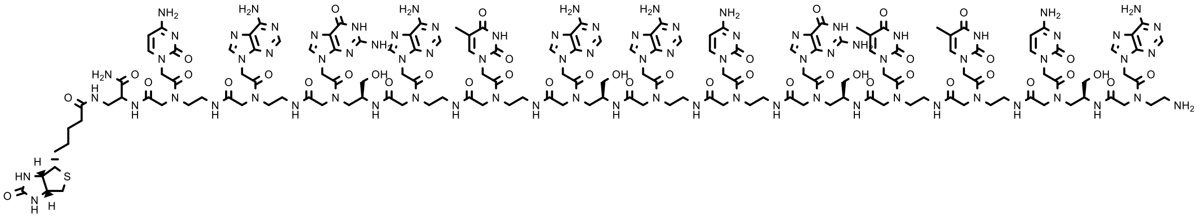

Chemical formula: $C_{157}H_{203}N_{81}O_{44}S$, MALDI-TOF-MS: m/z expected for $[M+H]^+$: 3959.5931, m/z found: 3960.252

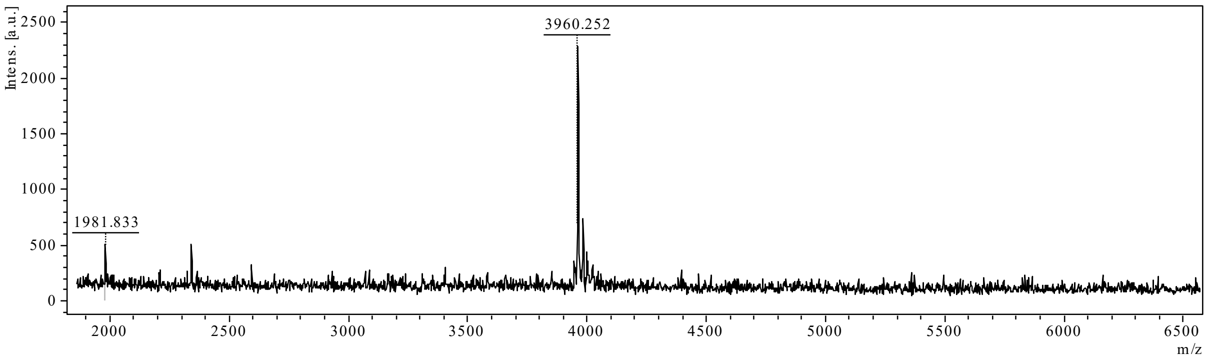

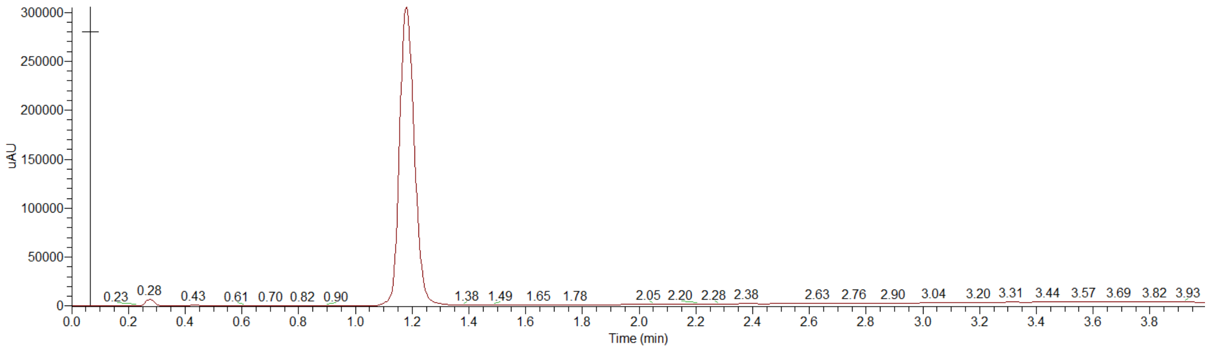

HRMS: expected for $[M+3H]^{3+}$: 1320.8703, m/z found: 1320.8678

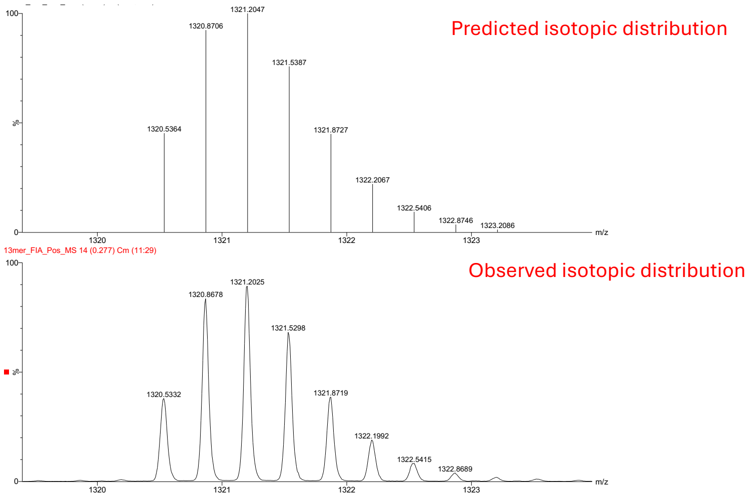

PNA template: C’ AGTCTGAACGTTATCTGA N’

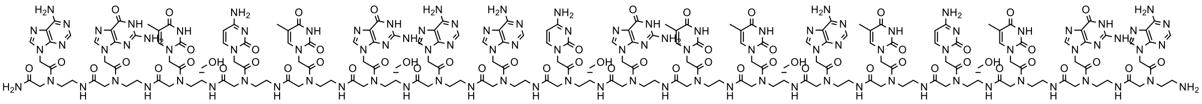

Chemical formula: $C_{200}H_{253}N_{103}O_{60}$, MALDI-TOF-MS: m/z expected for $[M+H]^+$: 5057.9985, m/z found: 5058.411

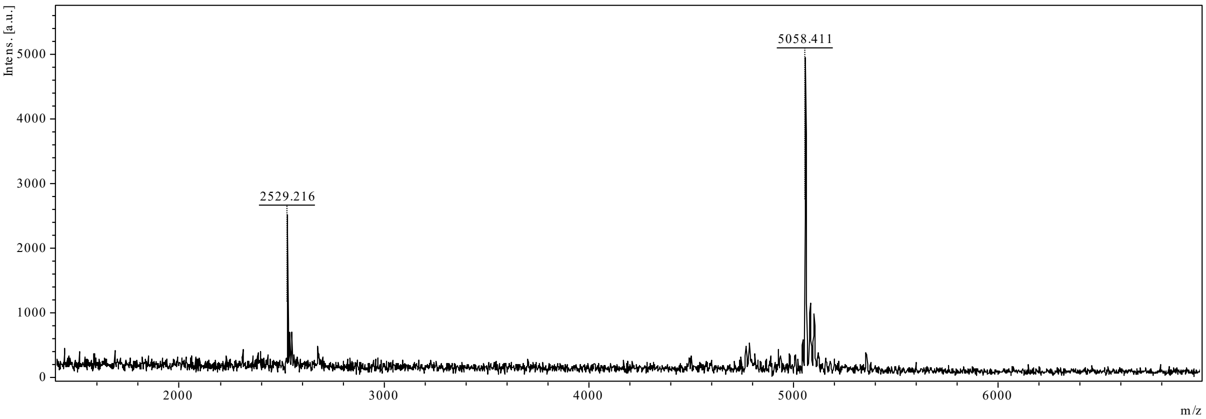

4-mers:

A1-

p-Nitrophenol-$CGTT_{Fmoc}$

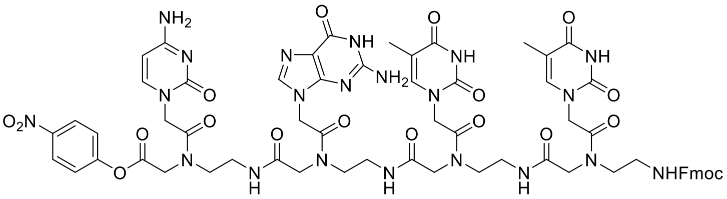

LCMS-ESI (m/z): $[M+H]^+$, calcd for $C_{64}H_{69}N_{21}O_{19}$, 1436.52; found, 1436.17

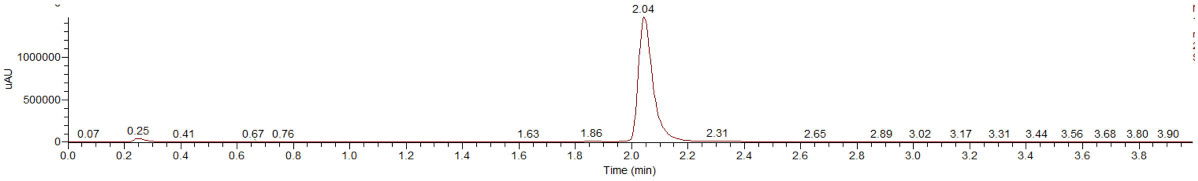

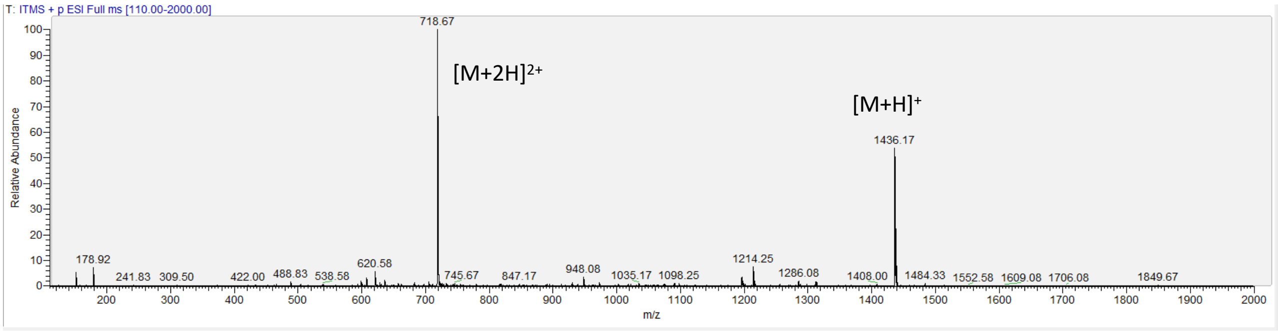

A2

p-Nitrophenol-GTTC$_{Fmoc}$

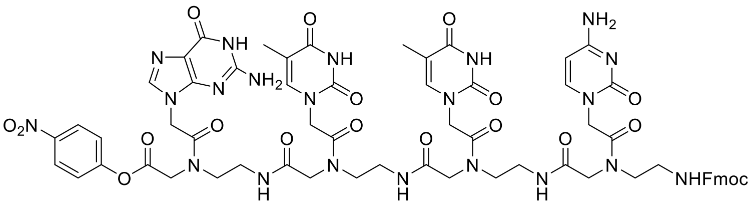

LCMS-ESI (m/z): $[M+H]^+$, calcd for $C_{64}H_{69}N_{21}O_{19}$, 1436.52; found, 1436.08

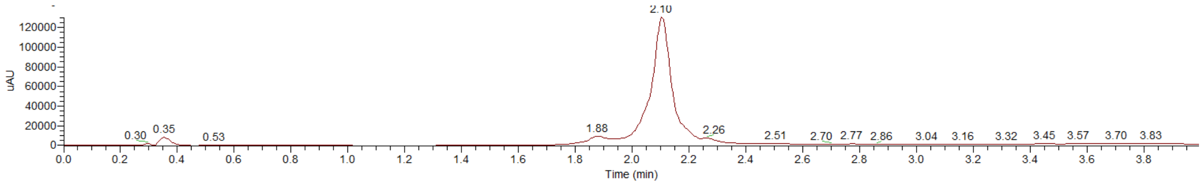

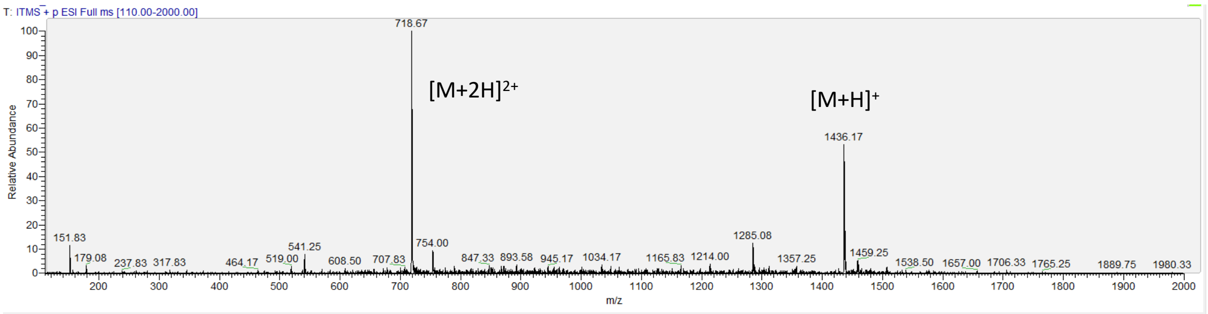

A3

p-Nitrophenol-TTCG$_{Fmoc}$

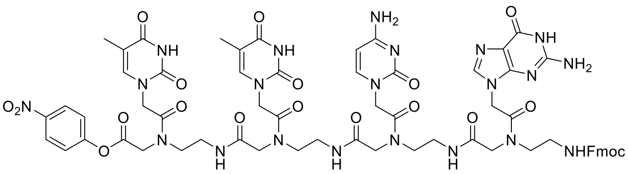

LCMS-ESI (m/z): [M+H]$^+$, calcd for $C_{64}H_{69}N_{21}O_{19}$, 1436.52; found, 1436.25

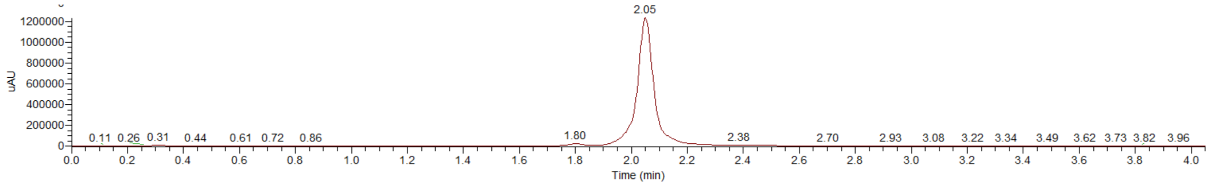

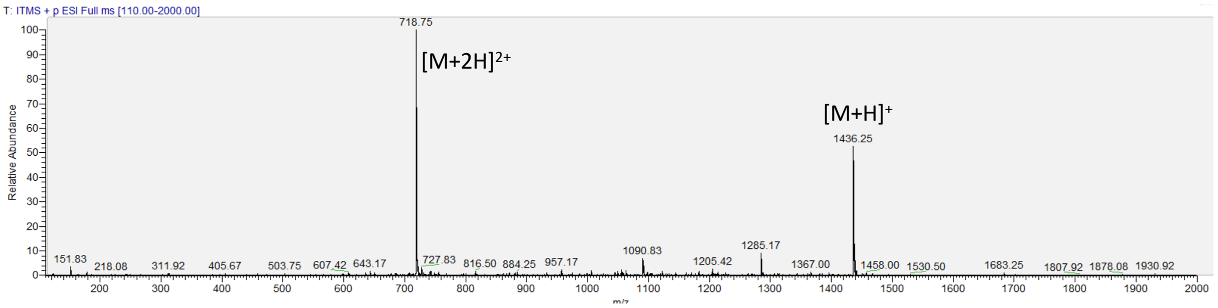

LCMS-ESI (m/z): [M+H]$^+$, calcd for $C_{64}H_{69}N_{21}O_{19}$, 1436.52; found, 1436.25

A4

p-Nitrophenol-TCGA$_{Fmoc}$

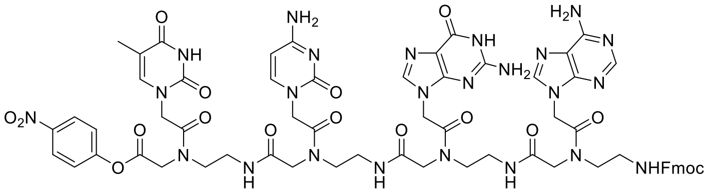

LCMS-ESI (m/z): [M+H]$^+$, calcd. for $C_{64}H_{68}N_{24}O_{17}$, 1445.53; found, 1445.17

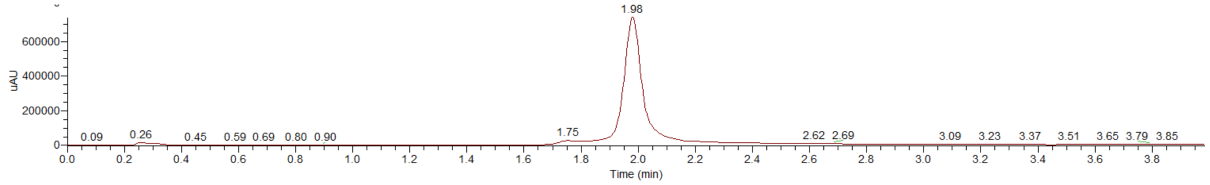

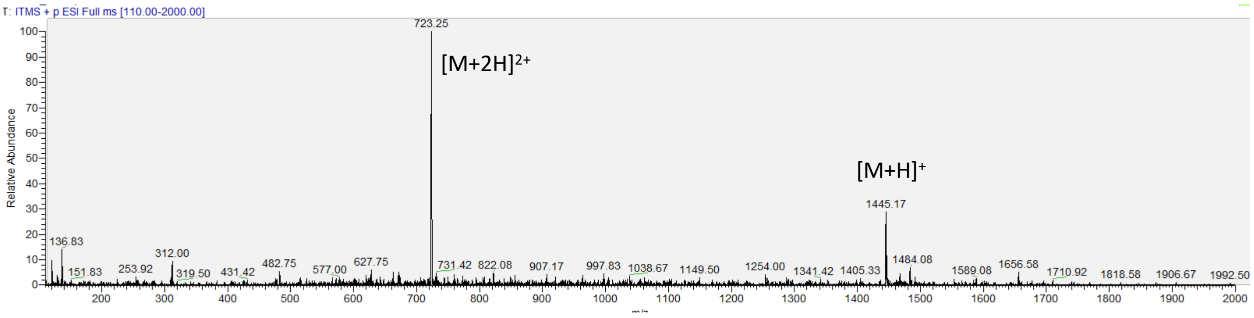

A5:

CCGT$_{N3}$

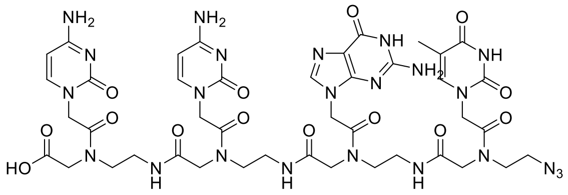

LCMS-ESI (m/z): [M+H]$^+$, calcd. for $C_{42}H_{53}N_{23}O_{14}$, 1104.42; found, 1104.33

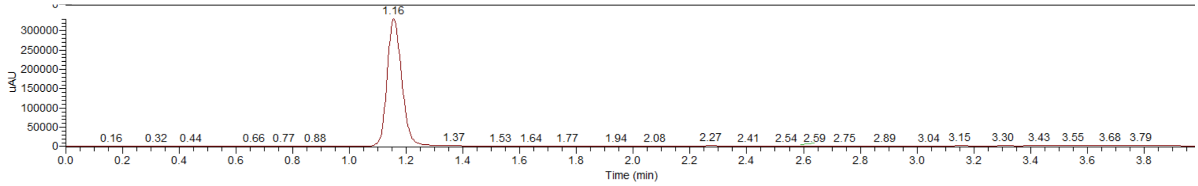

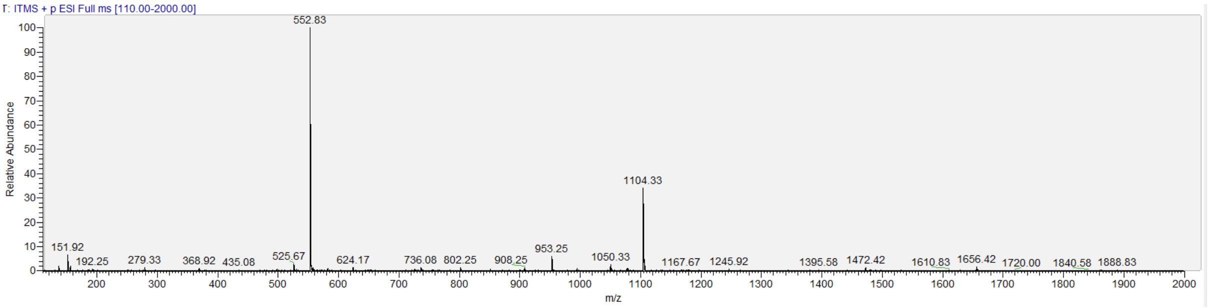

A6:

CGTT$_{N3}$

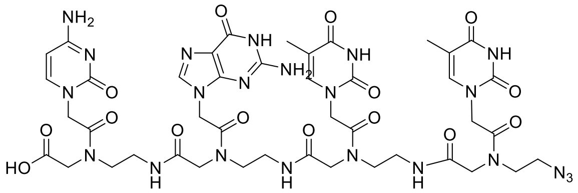

LCMS-ESI (m/z): [M+H]$^+$, calcd. for $C_{43}H_{54}N_{22}O_{15}$, 1119.42; found, 1119.33

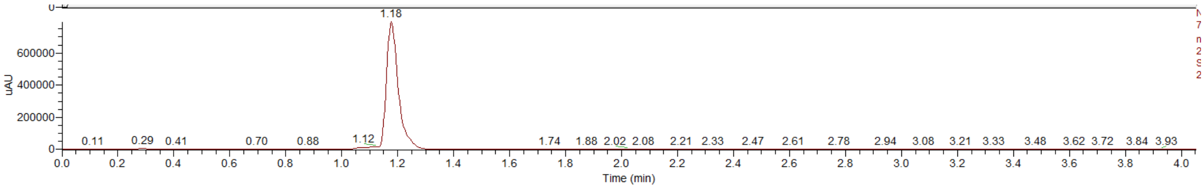

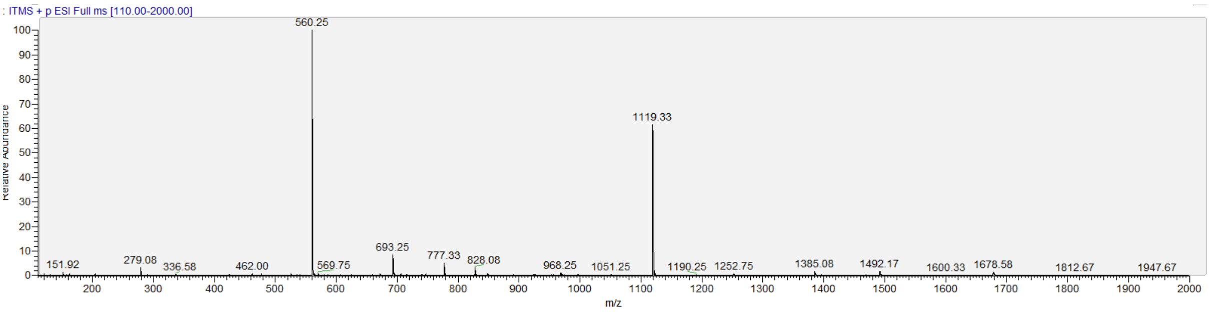

A7:

GTTC N3

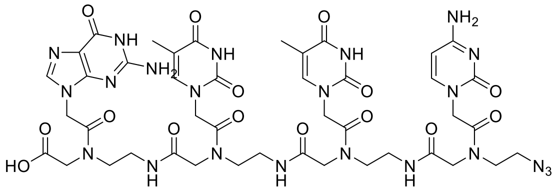

LCMS-ESI (m/z): $[M+H]^+$, calcd. for $C_{43}H_{54}N_{22}O_{15}$, 1119.42; found, 1119.33

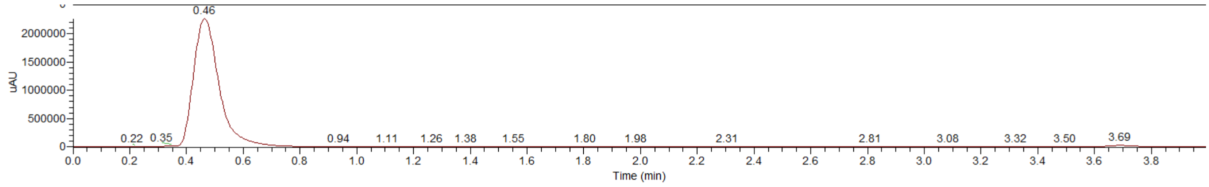

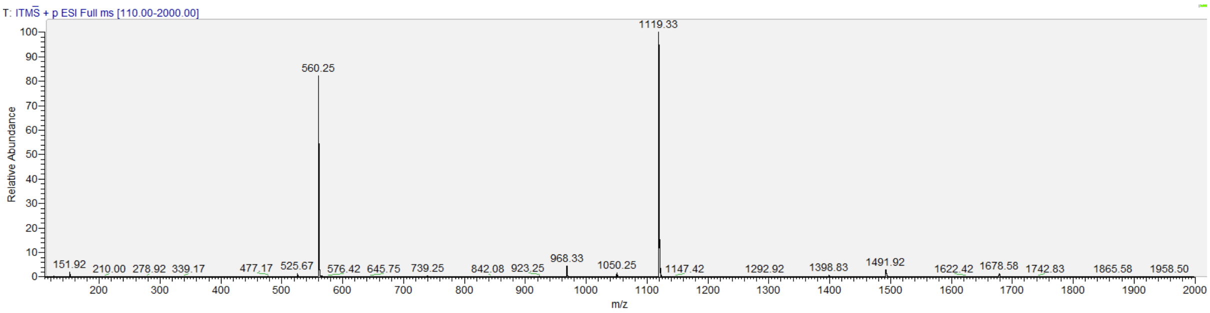

A8:

TTCG N3

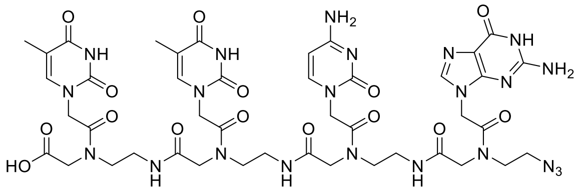

LCMS-ESI (m/z): $[M+H]^+$, calcd. for $C_{43}H_{54}N_{22}O_{15}$, 1119.42; found, 1119.33

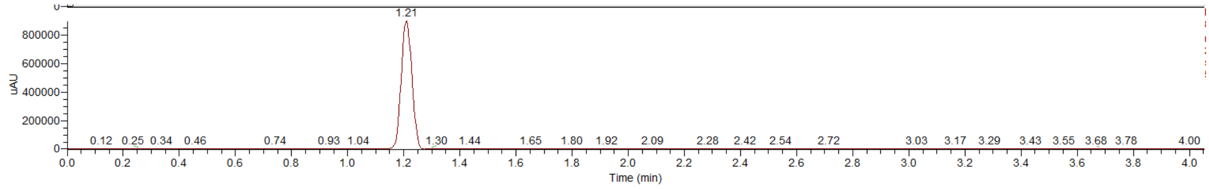

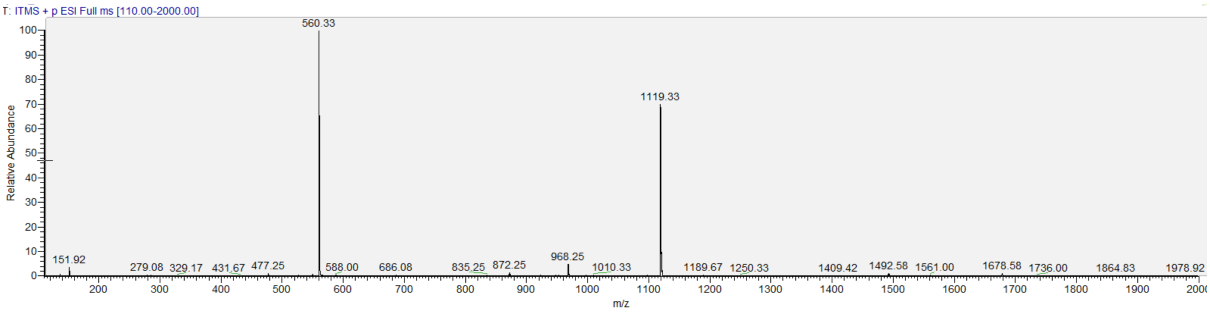

A9

TCGA$_{N3}$

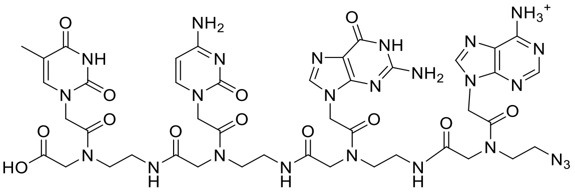

LCMS-ESI (m/z): $[M+H]^+$, calcd. for $C_{43}H_{53}N_{25}O_{13}$, 1128.43; found, 1128.33

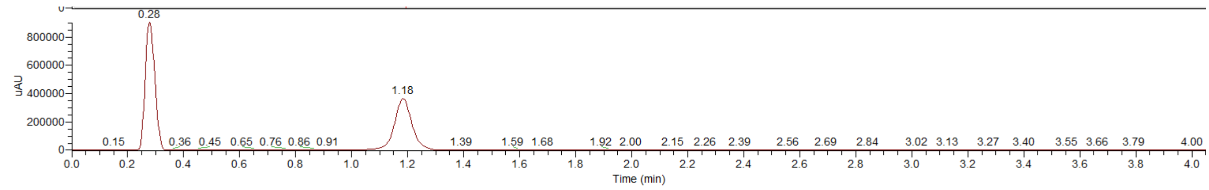

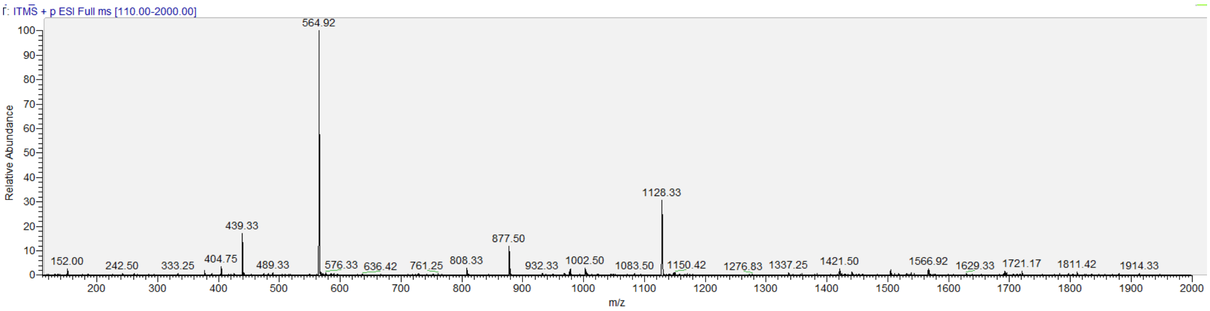

A10

CGAG$_{N3}$

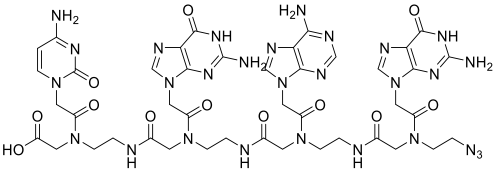

LCMS-ESI (m/z): $[M+H]^+$, calcd. for $C_{43}H_{52}N_{28}O_{12}$, 1153.44; found, 1153.33

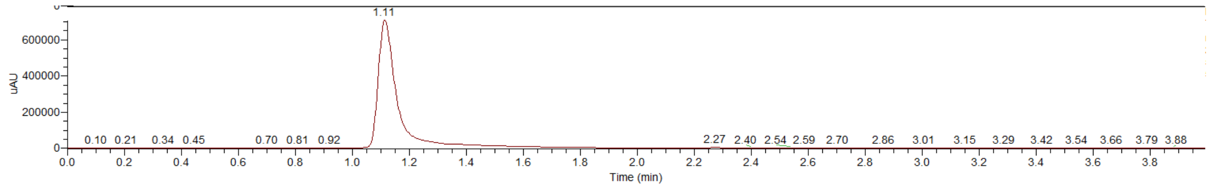

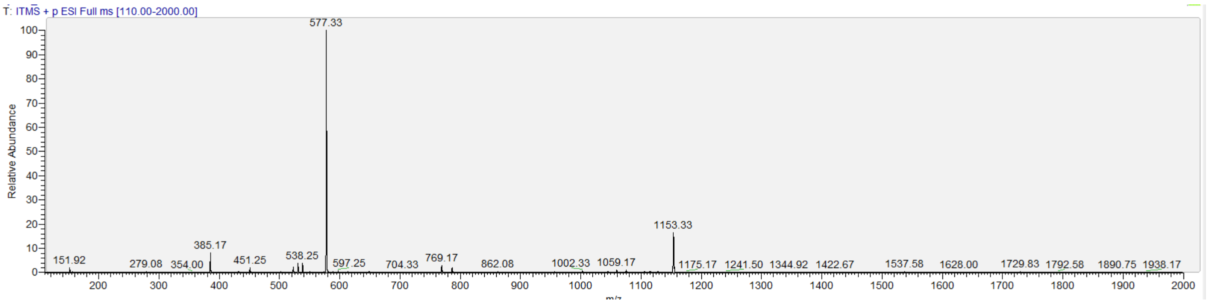

A11

GAGG$_{N3}$

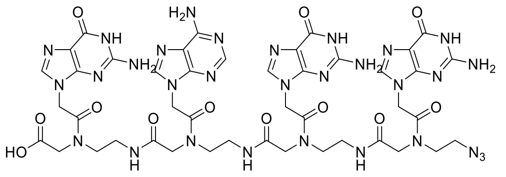

LCMS-ESI (m/z): $[M+H]^+$, calcd. for $C_{44}H_{52}N_{30}O_{12}$, 1193.45; found, 1193.33

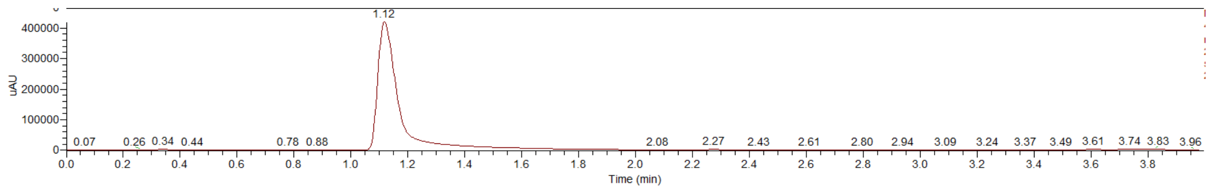

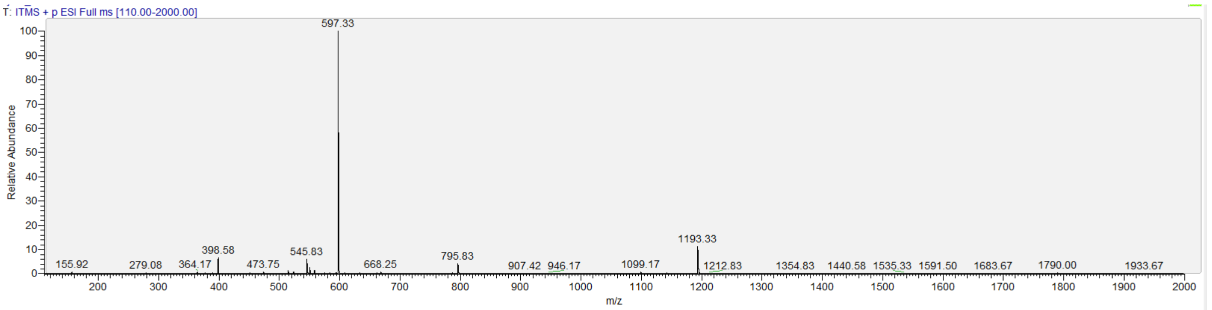

A12

AGGC$_{N3}$

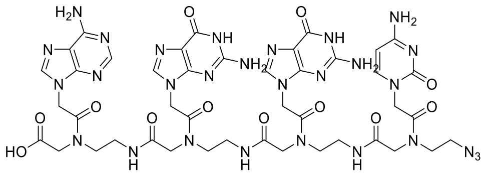

LCMS-ESI (m/z): $[M+H]^+$, calcd. for $C_{43}H_{52}N_{28}O_{12}$, 1153.44; found, 1153.42

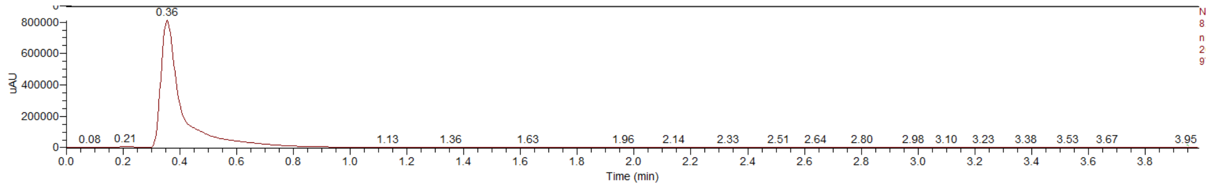

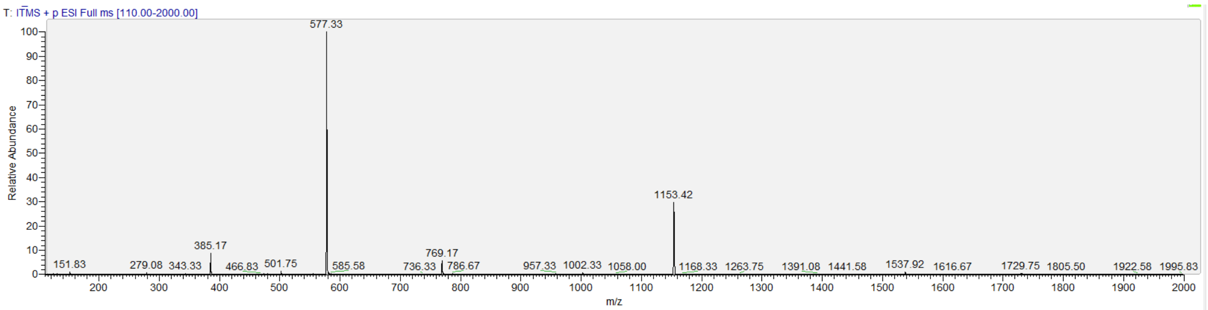

A13

CCGT$_{Fmoc}$

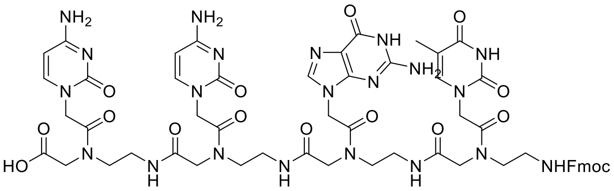

LCMS-ESI (m/z): $[M+H]^+$, calcd. for $C_{57}H_{65}N_{21}O_{16}$, 1300.50; found, 1300.83

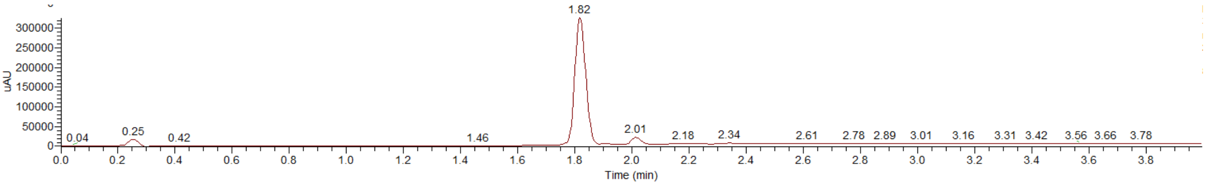

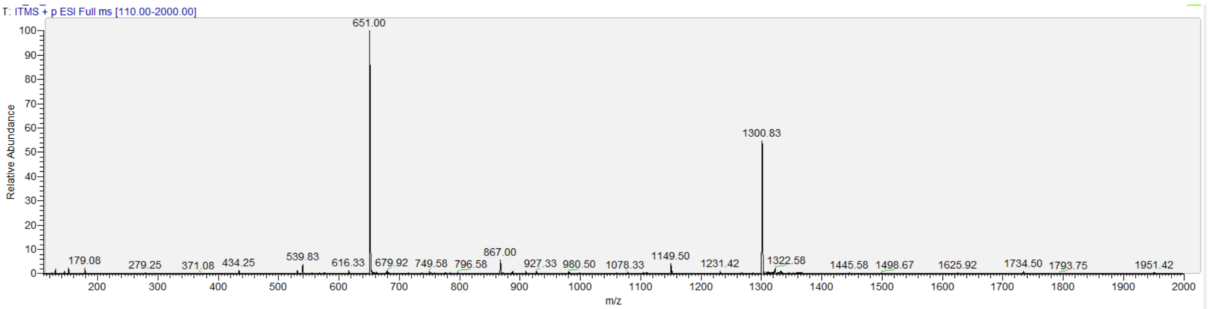

A14:

TCGT$_{Fmoc}$

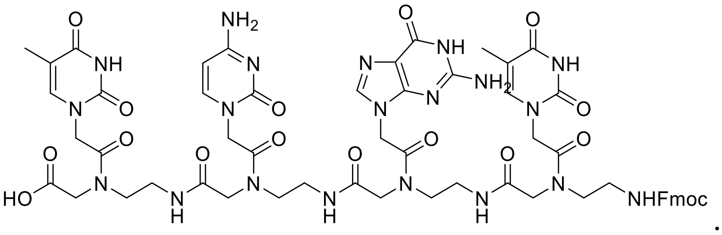

LCMS-ESI (m/z): $[M+H]^+$, calcd. for $C_{58}H_{66}N_{20}O_{17}$, 1315.50; found, 1316.33

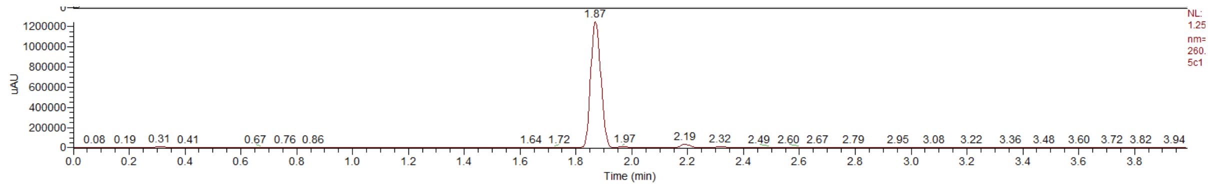

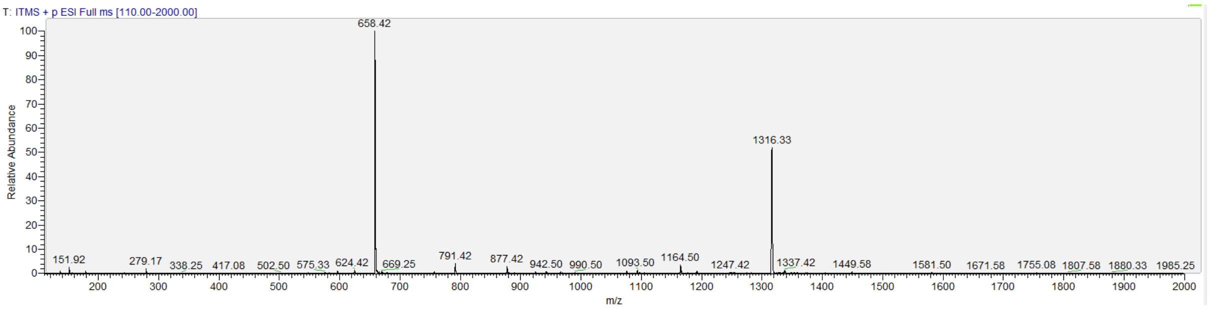

A15:

GGGT$_{Fmoc}$

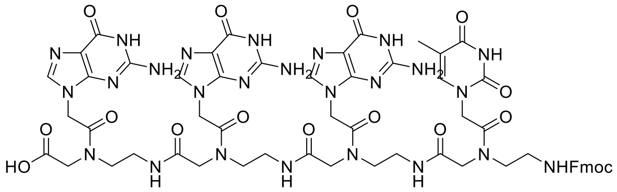

LCMS-ESI (m/z): [M+H]$^+$, calcd. for $C_{59}H_{65}N_{25}O_{16}$, 1380.51; found, 1381.08

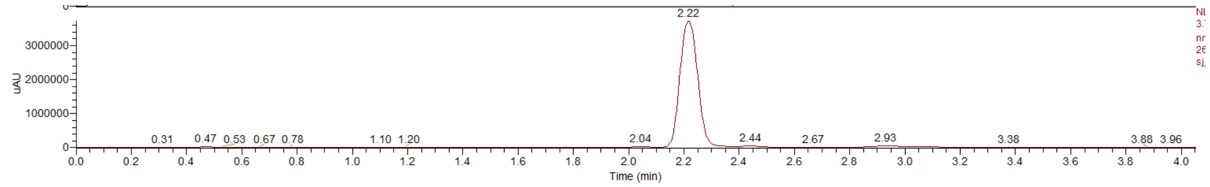

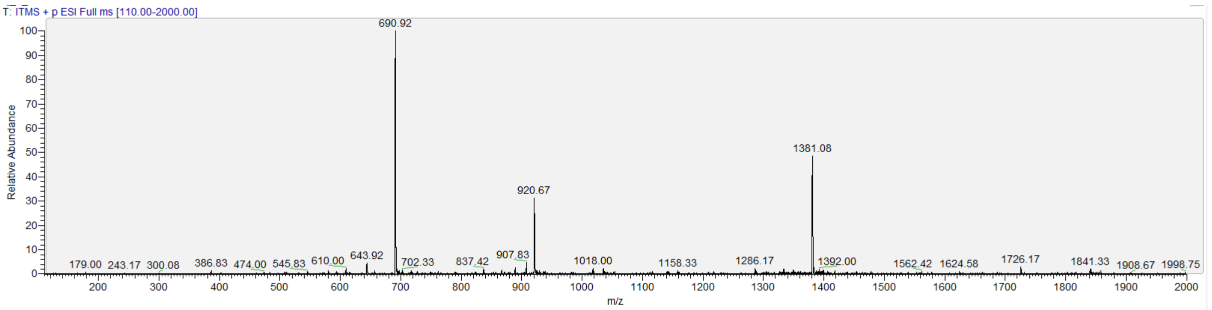

A16:

CGAG$_{N3}$

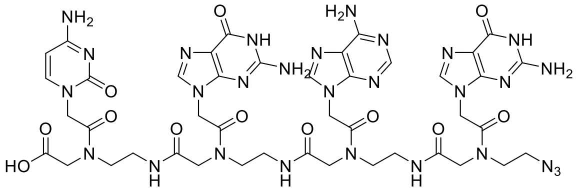

LCMS-ESI (m/z): [M+H]+, calcd. for $C_{43}H_{58}N_{28}O_{12}$, 1153.44; found, 1153.33

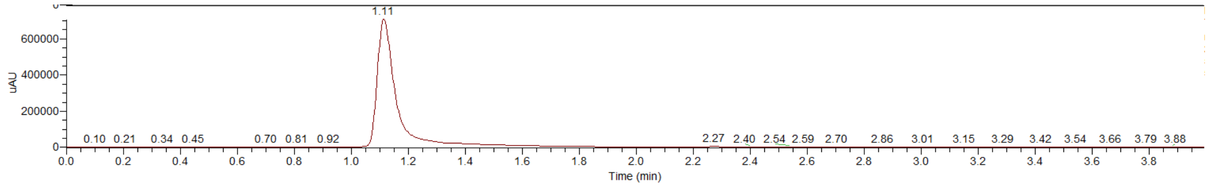

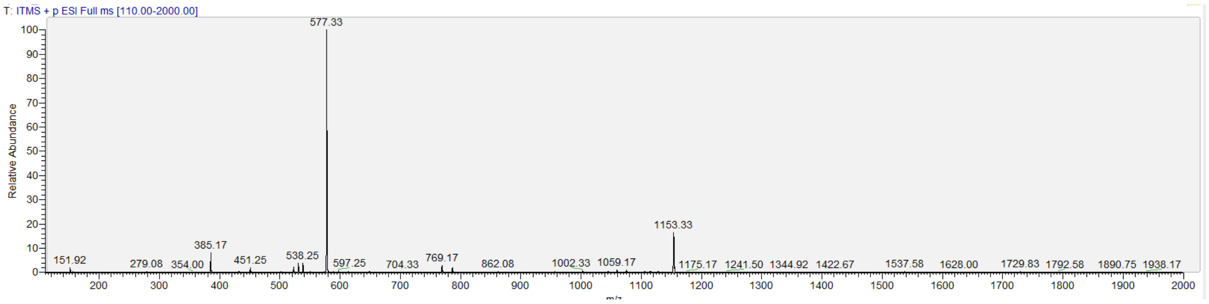

A17:

GTCC$_{N3}$

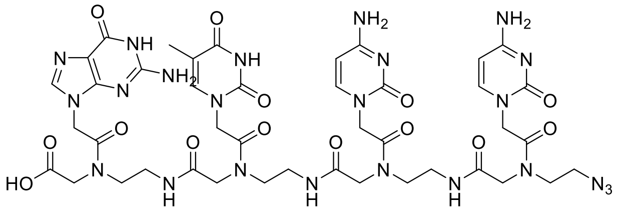

LCMS-ESI (m/z): [M+H]+, calcd. for $C_{42}H_{53}N_{23}O_{14}$, 1104.42; found, 1104.42

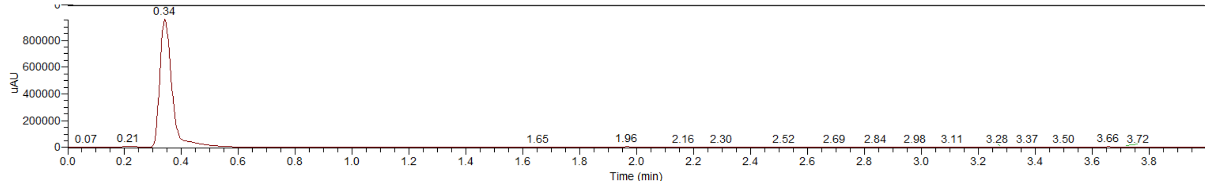

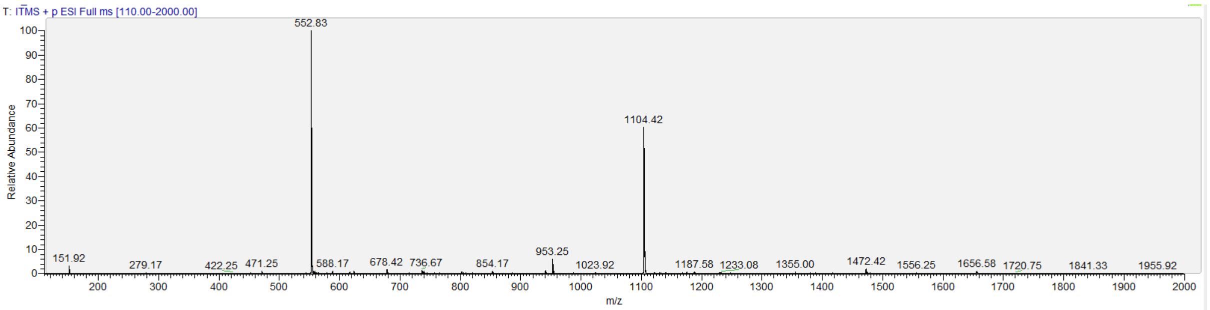

A18:

CCTA$_{Fmoc}$

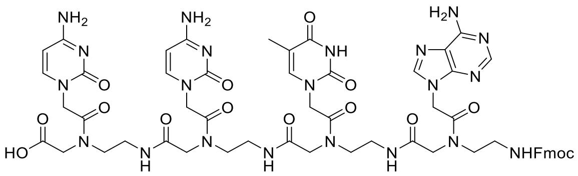

LCMS-ESI (m/z): $[M+H]^+$, calcd. for $C_{57}H_{65}N_{21}O_{15}$, 1284.50; found, 1284.50

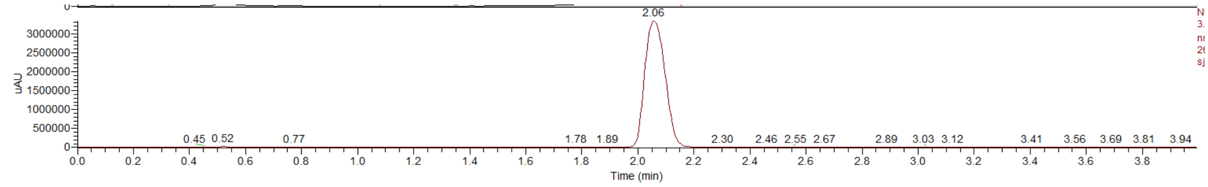

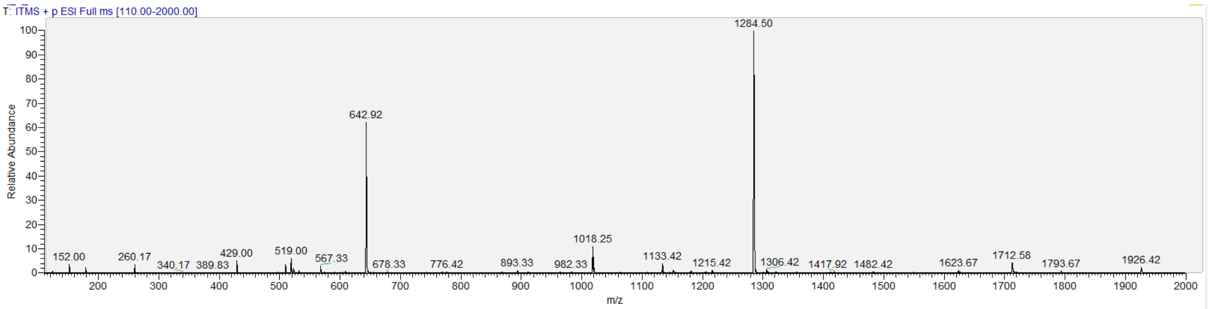

A19:

CCAT$_{Fmoc}$

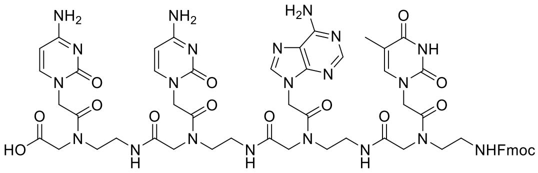

LCMS-ESI (m/z): $[M+H]^+$, calcd. for $C_{57}H_{65}N_{21}O_{15}$, 1284.50; found, 1284.50

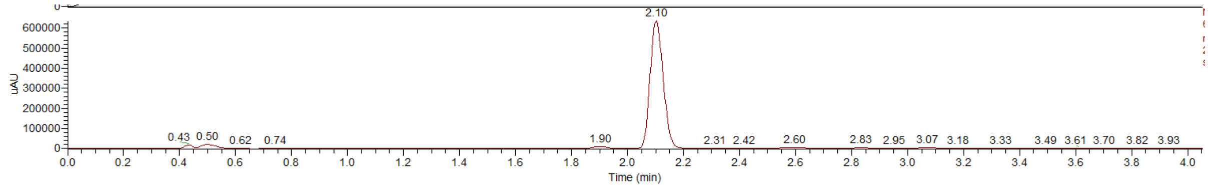

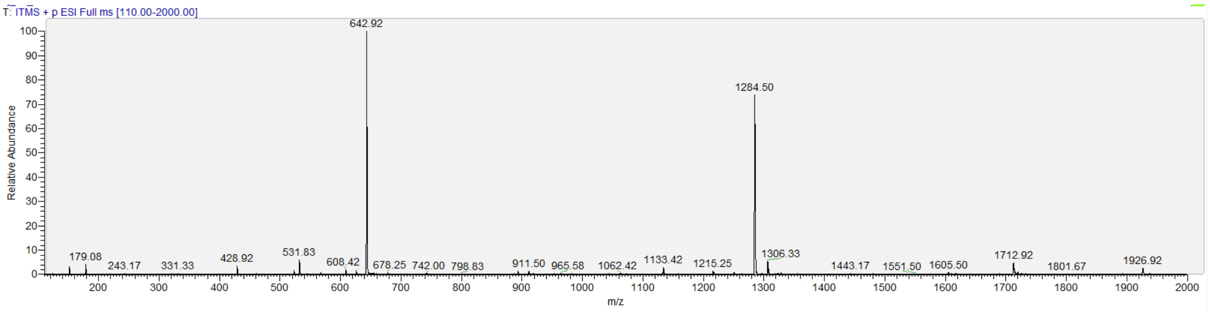

A20:

$CTGT_{Fmoc}$

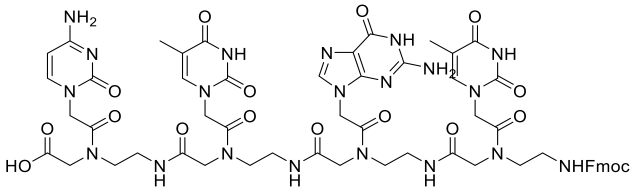

LCMS-ESI (m/z): [M+H]$^+$, calcd. for $C_{58}H_{66}N_{20}O_{17}$, 1315.50; found, 1315.67

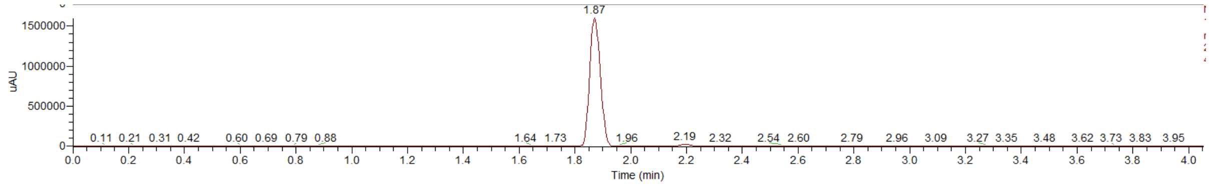

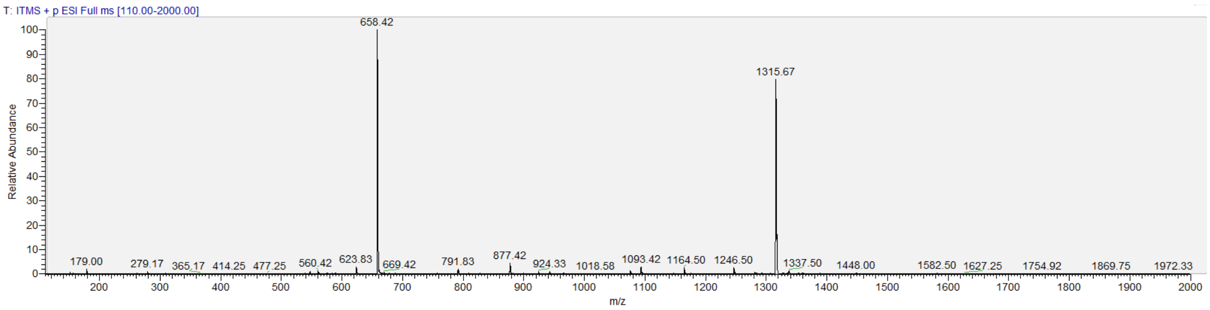

A21

$CCGA_{Fmoc}$

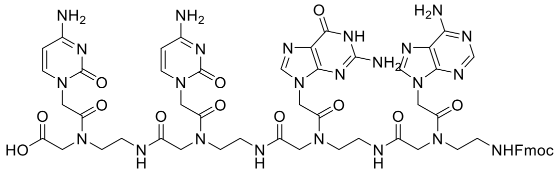

LCMS-ESI (m/z): [M+H]$^+$, calcd. for $C_{57}H_{64}N_{24}O_{14}$, 1309.51; found, 1310.42

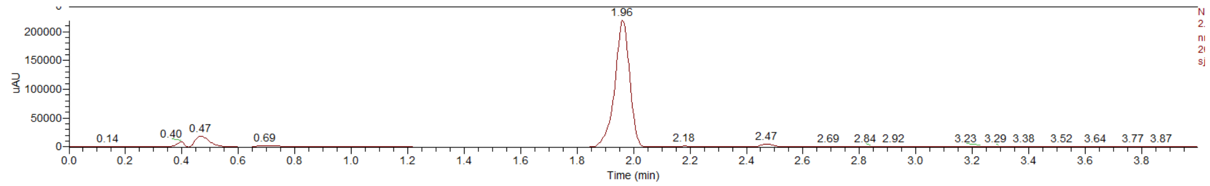

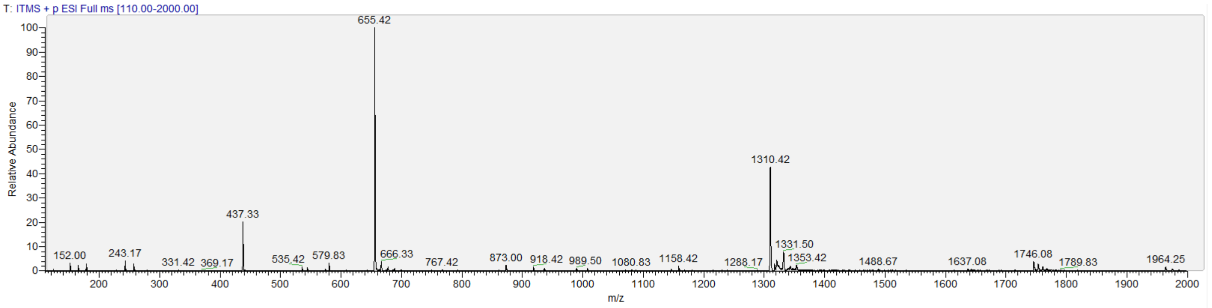

A22

ACGG$_{Fmoc}$

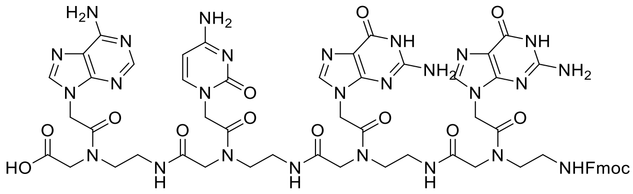

LCMS-ESI (m/z): $[M+H]^+$, calcd. for $C_{58}H_{64}N_{26}O_{14}$, 1349.52; found, 1349.75

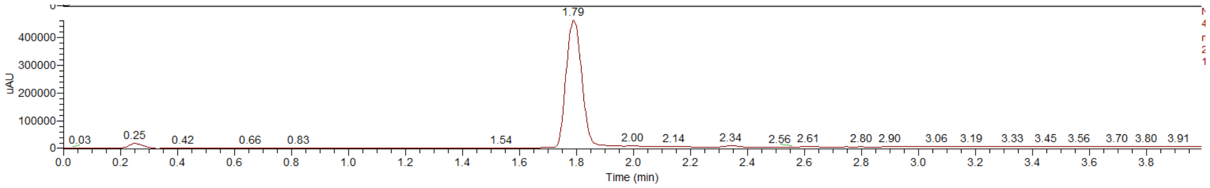

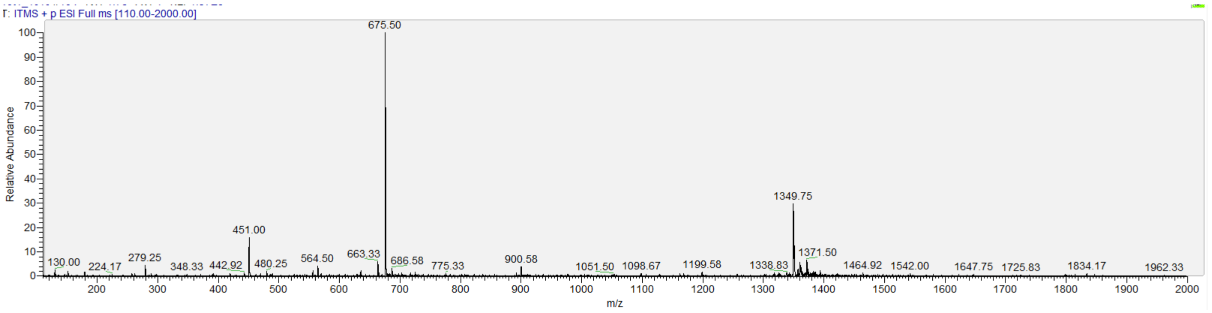

A23:

TCGG$_{Fmoc}$

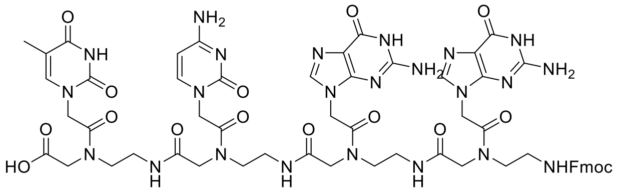

LCMS-ESI (m/z): $[M+H]^+$, calcd. for $C_{58}H_{65}N_{23}O_{16}$, 1340.51; found, 1340.50

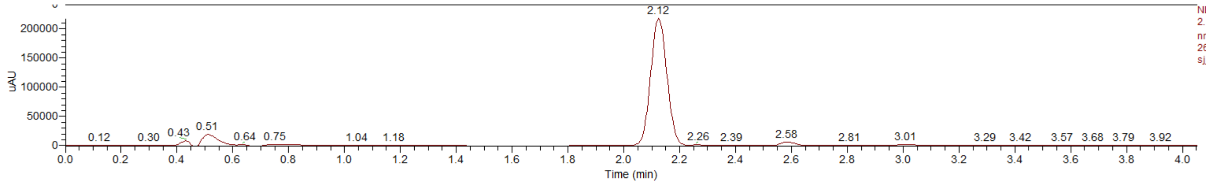

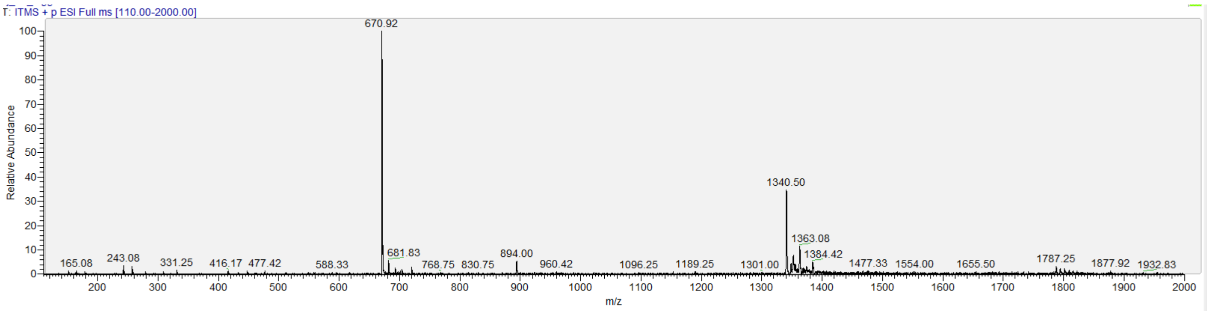

A24

ACGT$_{Fmoc}$

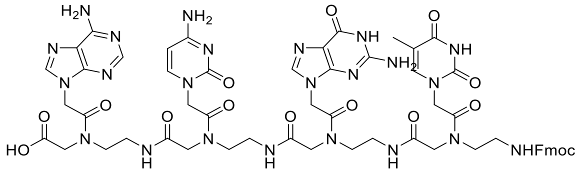

LCMS-ESI (m/z): $[M+H]^+$, calcd. for $C_{58}H_{65}N_{23}O_{15}$, 1324.51; found, 1325.42

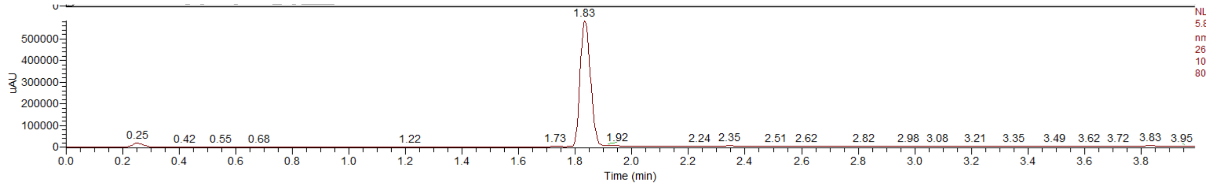

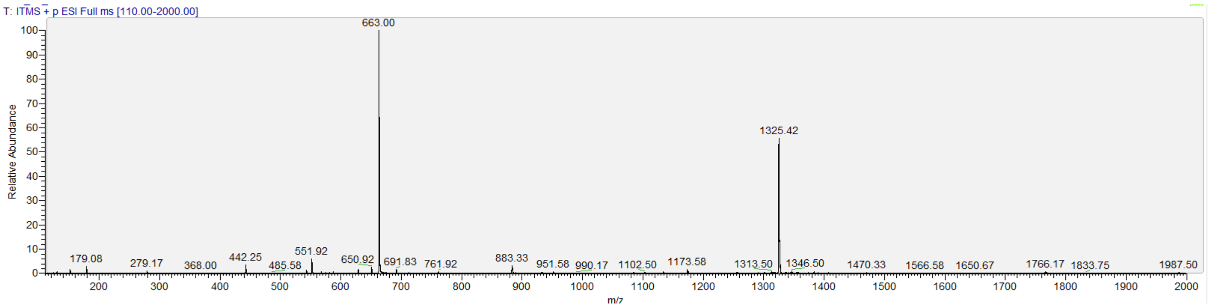

A25

GCGT$_{Fmoc}$

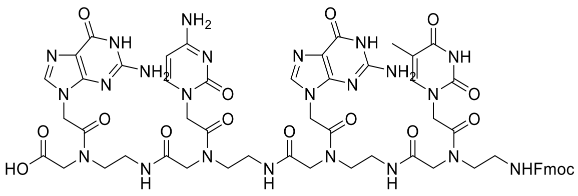

LCMS-ESI (m/z): $[M+H]^+$, calcd. for $C_{58}H_{65}N_{23}O_{16}$, 1340.51; found, 1341.33

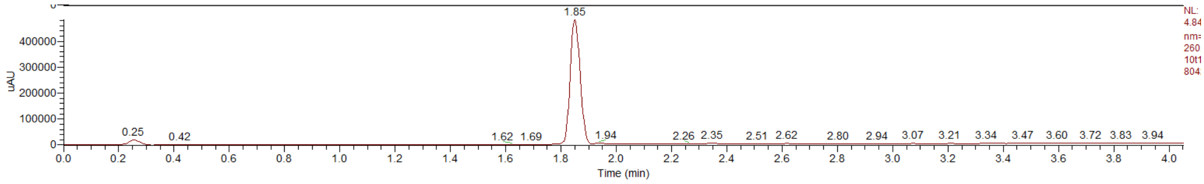

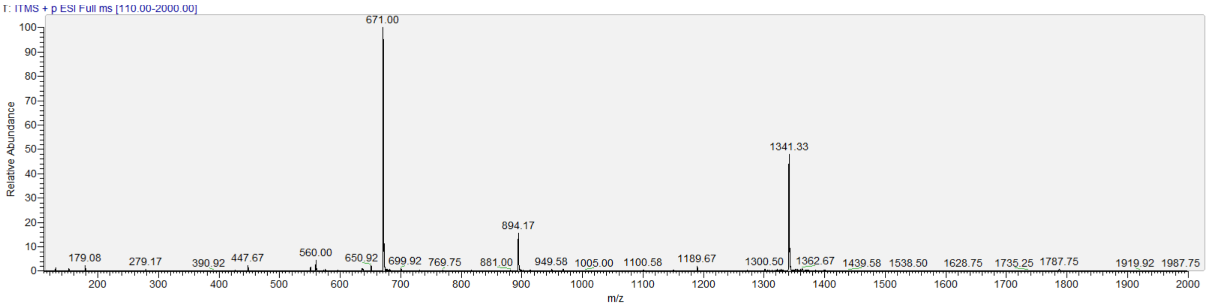

A26

ACGA$_{Fmoc}$

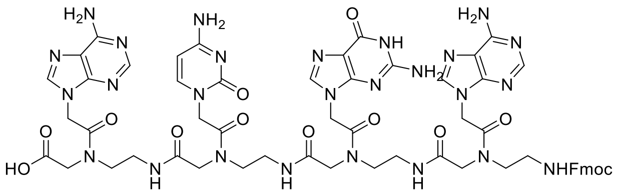

LCMS-ESI (m/z): $[M+H]^+$, calcd. for $C_{58}H_{64}N_{26}O_{13}$, 1333.52; found, 1334.50

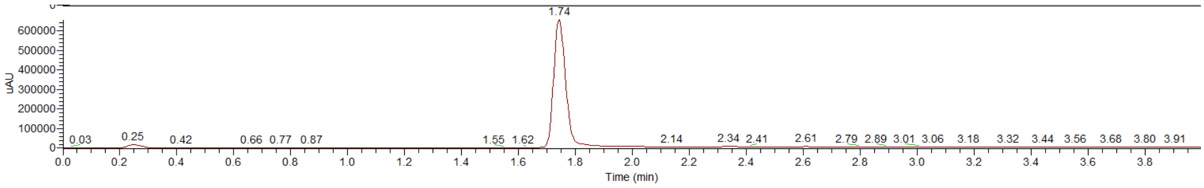

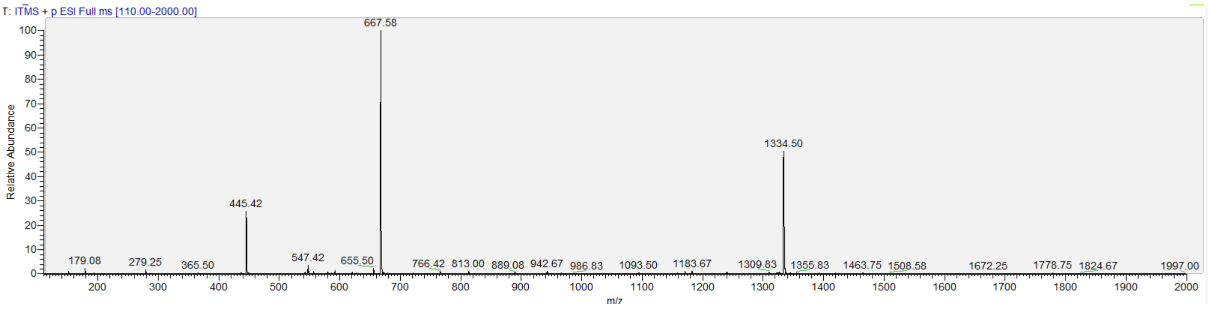

A27

ACGC$_{Fmoc}$

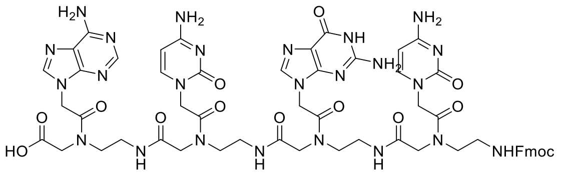

LCMS-ESI (m/z): $[M+H]^+$, calcd. for $C_{57}H_{64}N_{24}O_{14}$, 1309.51; found, 1309.83

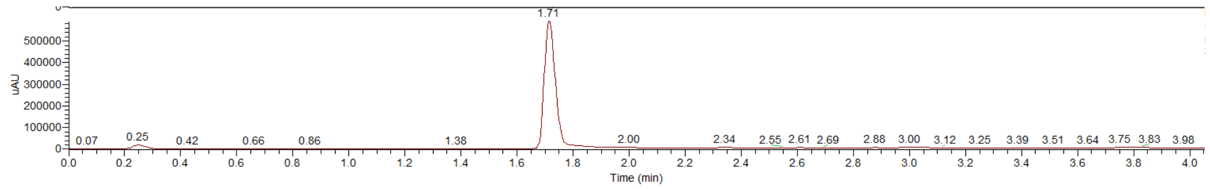

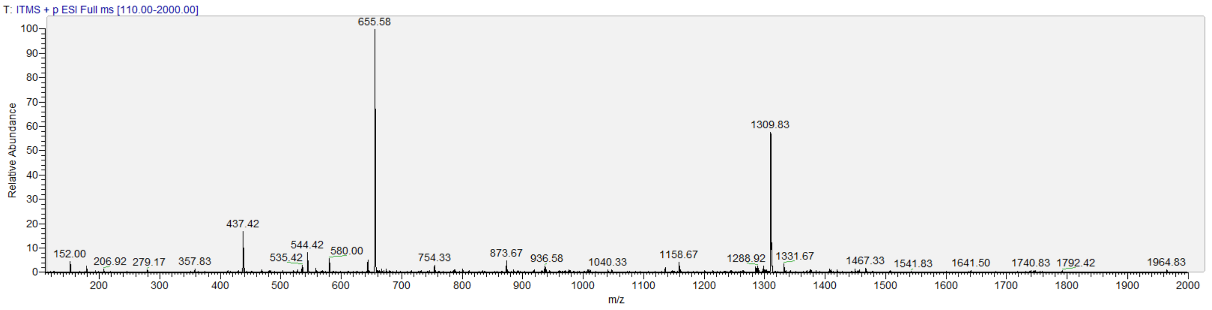

A28

CCGC$_{Fmoc}$

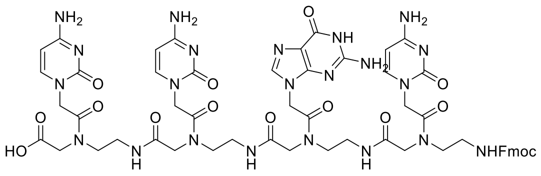

LCMS-ESI (m/z): $[M+H]^+$, calcd. for $C_{56}H_{64}N_{22}O_{15}$, 1285.50; found, 1286.00

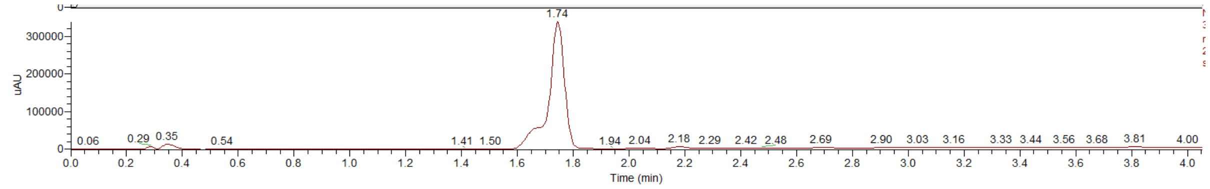

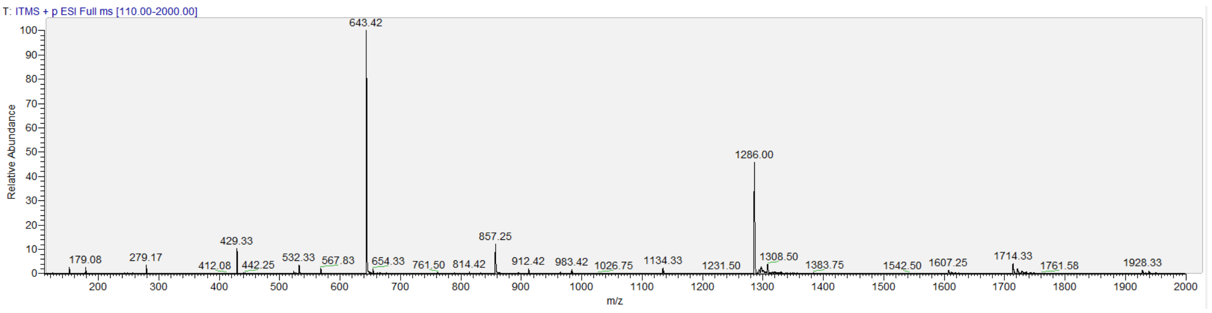

A29

CCGG$_{Fmoc}$

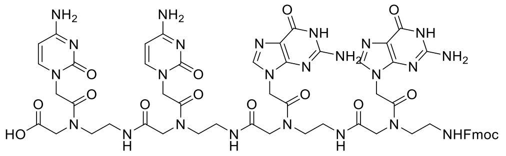

LCMS-ESI (m/z): $[M+H]^+$, calcd. for $C_{57}H_{64}N_{24}O_{15}$, 1325.51; found, 1325.83

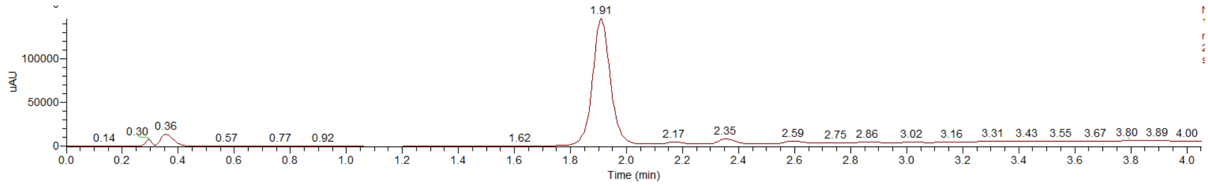

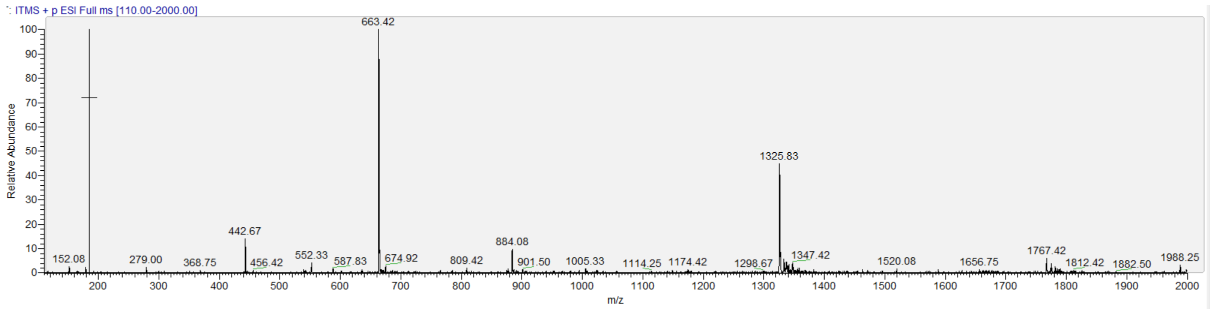

A30

GCGG$_{Fmoc}$

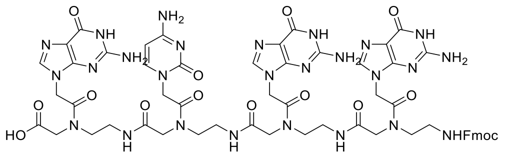

LCMS-ESI (m/z): $[M+H]^+$, calcd. for $C_{58}H_{64}N_{26}O_{15}$, 1365.51; found, 1366.50

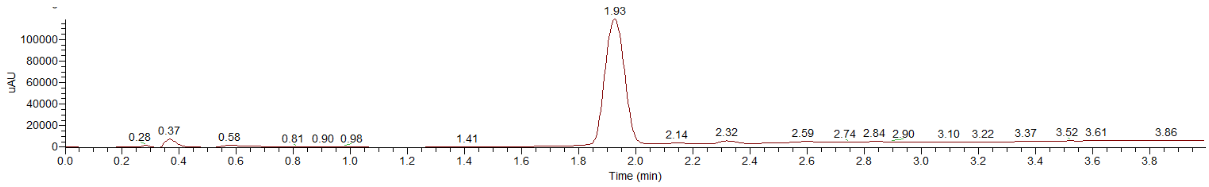

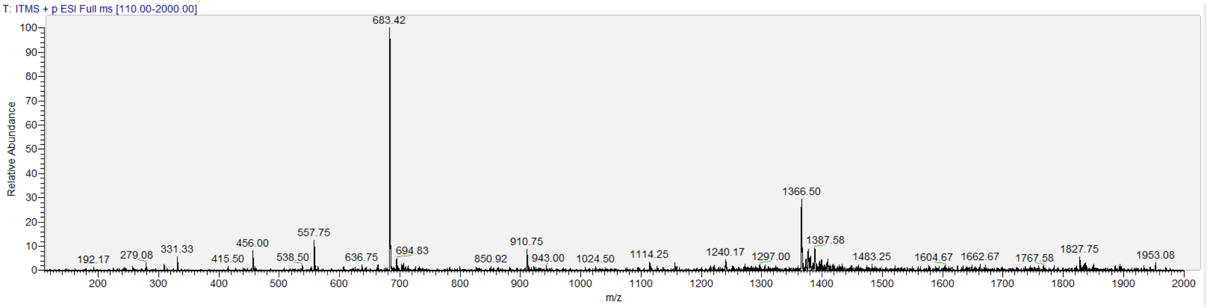

A31

$CCGA_{N3}$

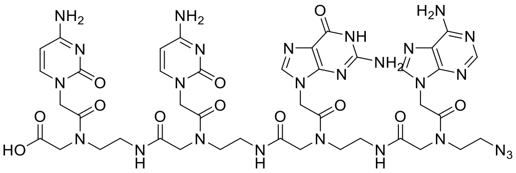

LCMS-ESI (m/z): $[M+H]^+$, calcd. for $C_{42}H_{52}N_{26}O_{12}$, 1113.43; found, 1113.50

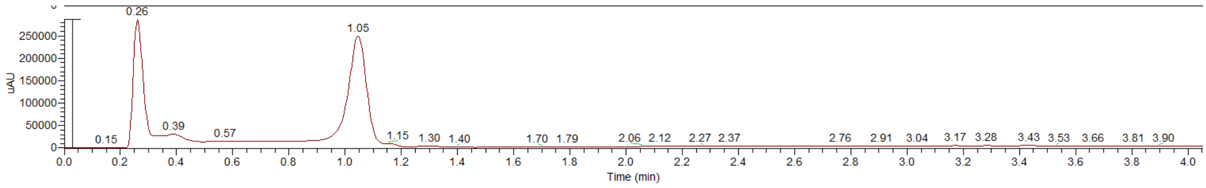

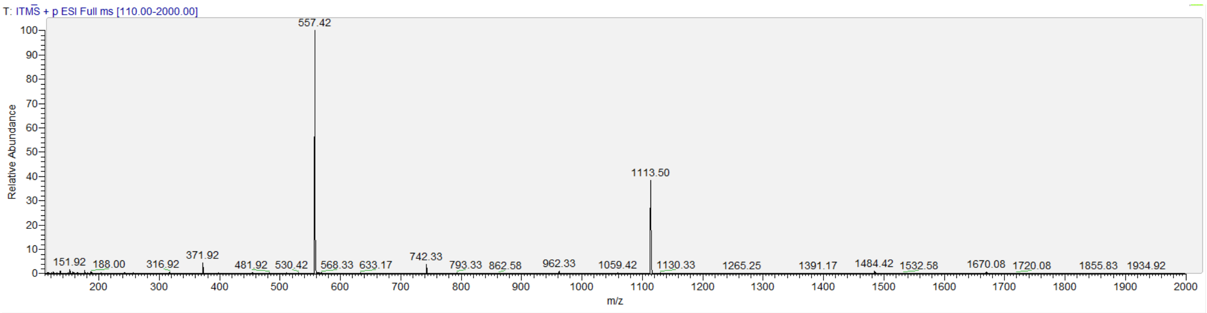

A32

$TCAG_{Fmoc}$

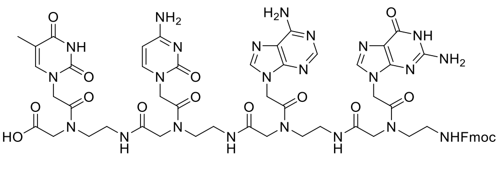

LCMS-ESI (m/z): $[M+H]^+$, calcd. for $C_{58}H_{65}N_{23}O_{15}$, 1324.51; found, 1325.33

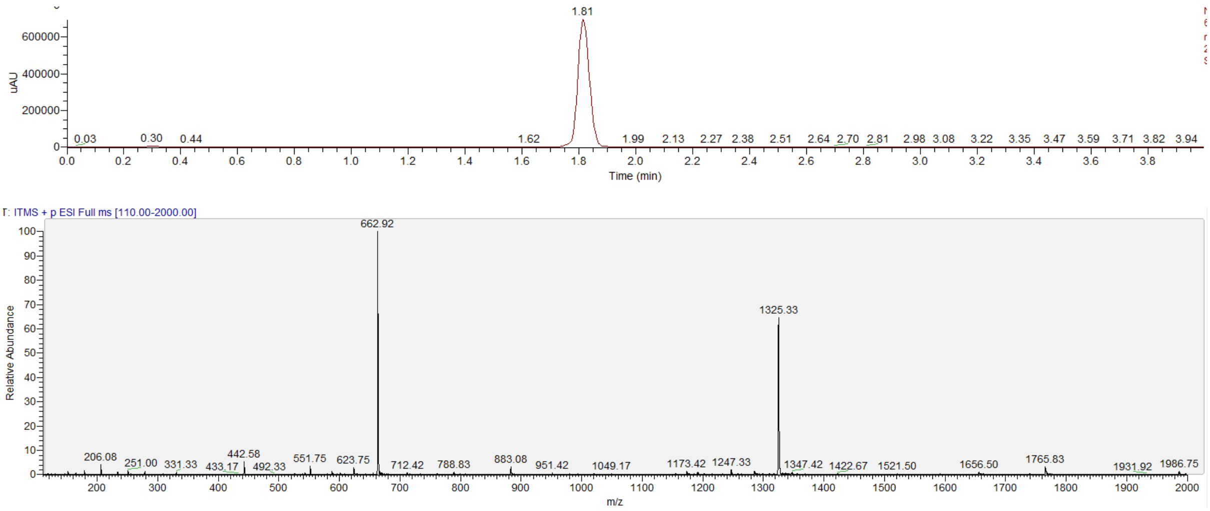

A33

CAGA$_{Fmoc}$

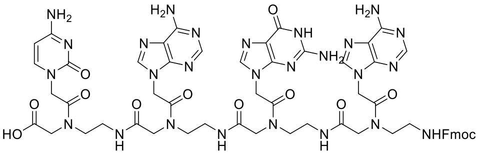

LCMS-ESI (m/z): [M+H]$^+$, calcd. for $C_{58}H_{64}N_{26}O_{13}$, 1333.52; found, 1334.33

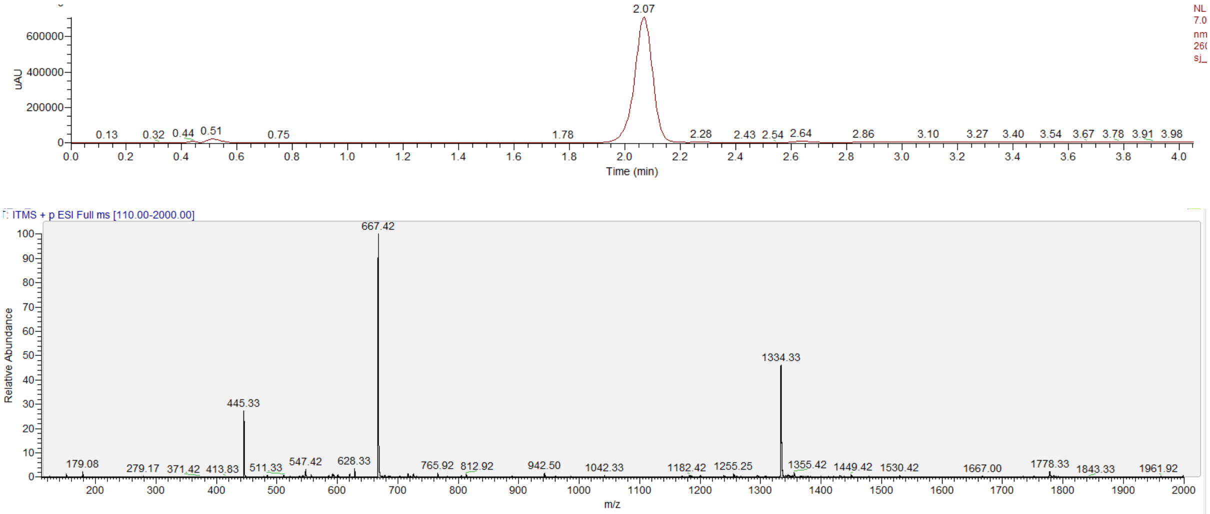

A34

AGAC$_{Fmoc}$

LCMS-ESI (m/z): [M+H]$^+$, calcd. for $C_{58}H_{64}N_{26}O_{13}$, 1333.52; found, 1334.25

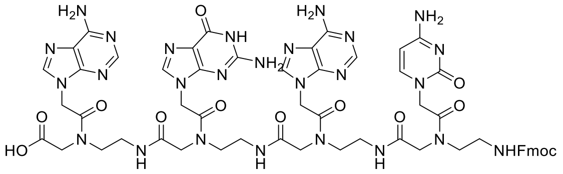

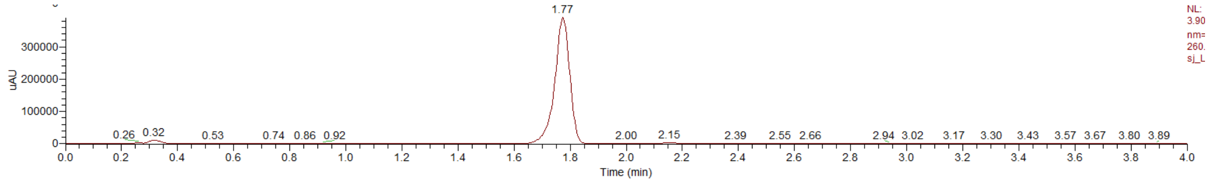

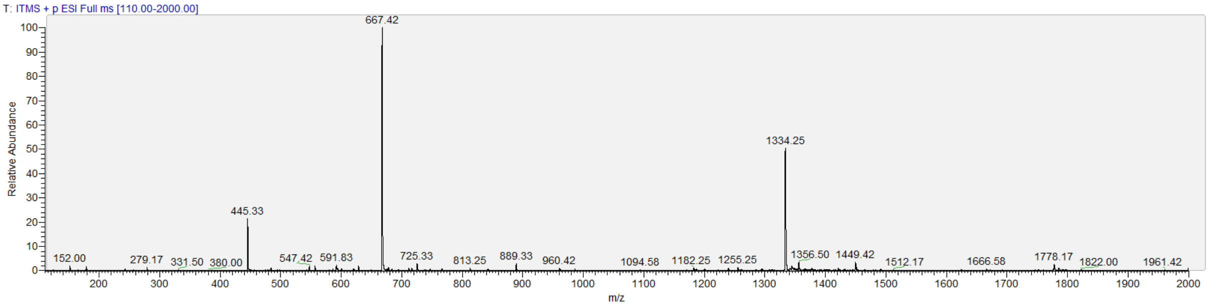

A35

GACT$_{Fmoc}$

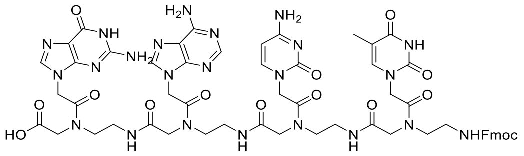

LCMS-ESI (m/z): $[M+H]^{+}$, calcd. for $C_{58}H_{65}N_{23}O_{15}$, 1324.51; found, 1325.33

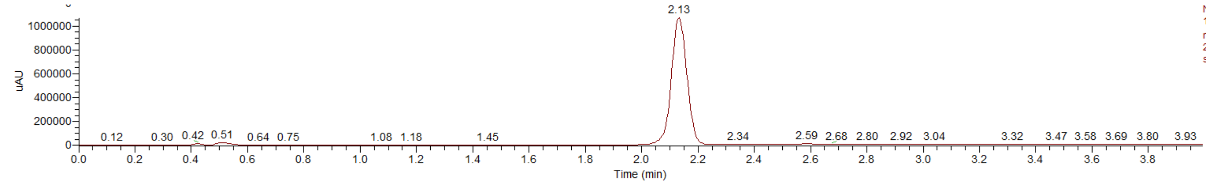

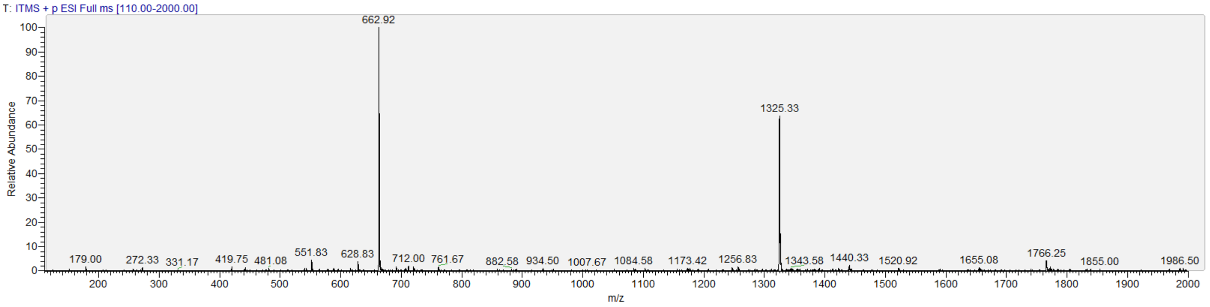

A36

CTGA$_{Fmoc}$

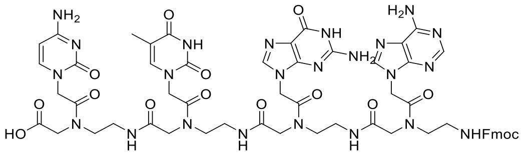

LCMS-ESI (m/z): [M+H]$^+$, calcd. for $C_{58}H_{65}N_{23}O_{15}$, 1324.51; found, 1325.00

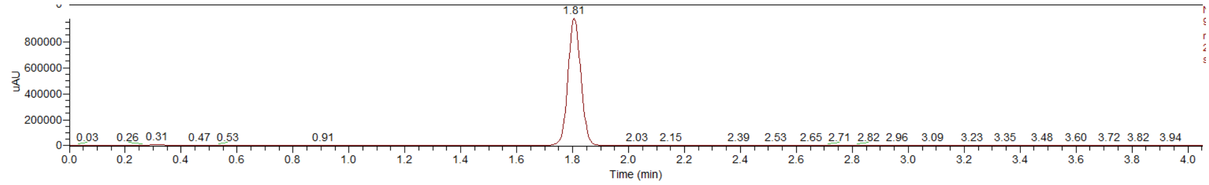

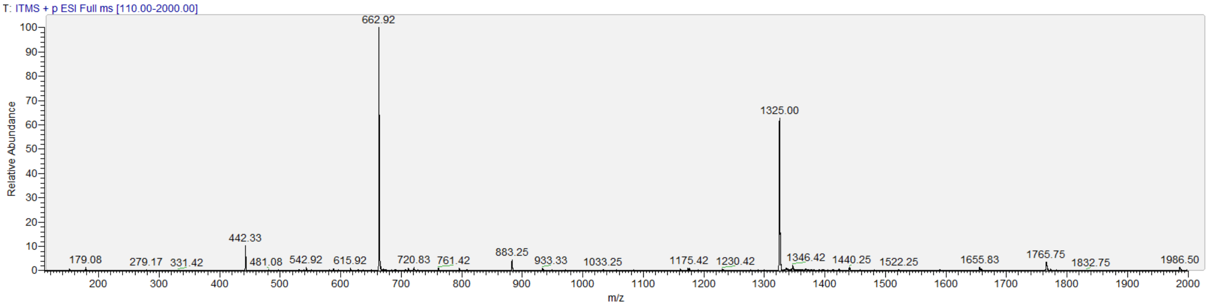

A37

TCTG$_{Fmoc}$

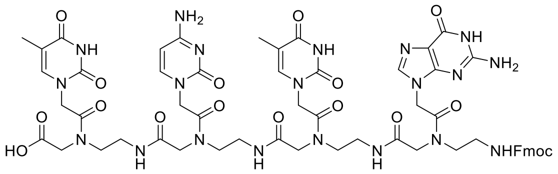

LCMS-ESI (m/z): [M+H]$^+$, calcd. for $C_{58}H_{66}N_{20}O_{17}$, 1315.50; found, 1315.58

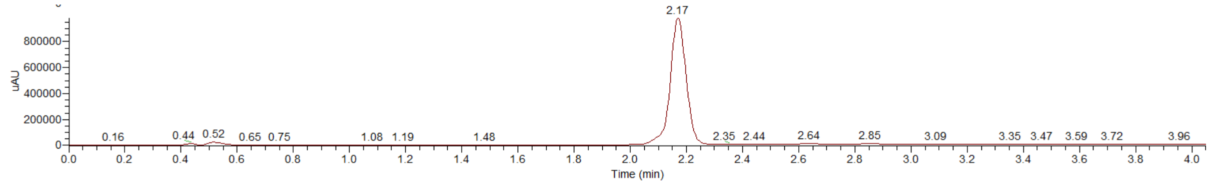

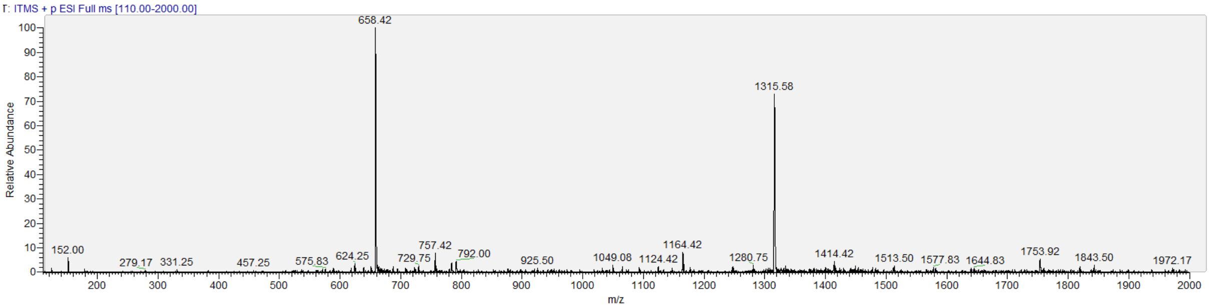

A38

GTCT$_{Fmoc}$

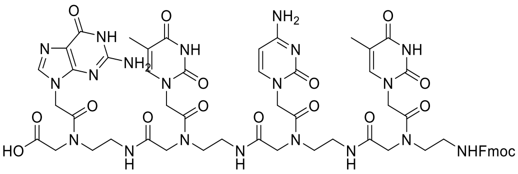

LCMS-ESI (m/z): $[M+H]^+$, calcd. for $C_{58}H_{66}N_{20}O_{17}$, 1315.50; found, 1315.58

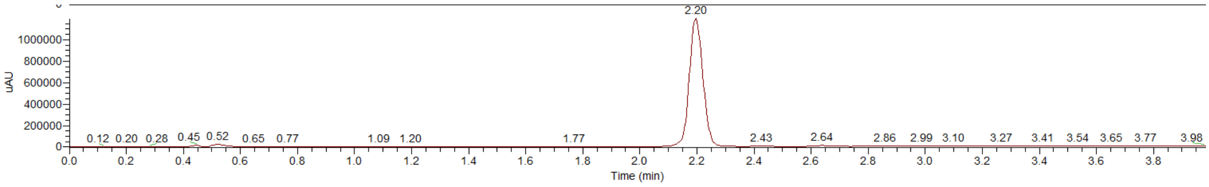

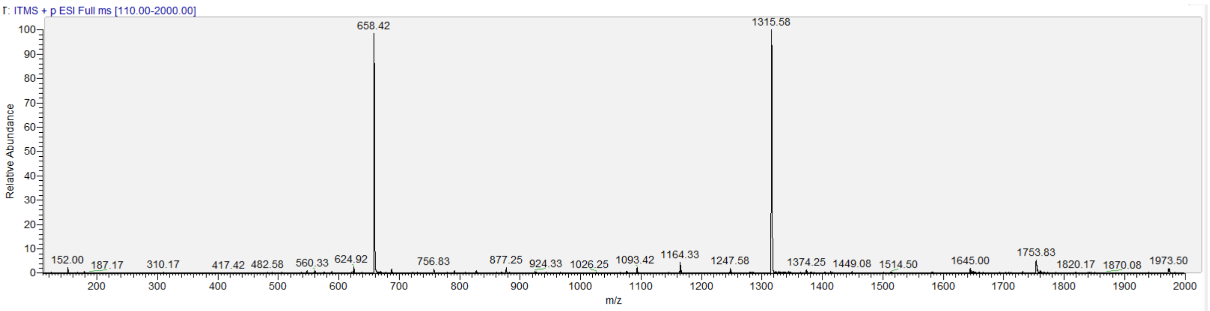

A39

AGTC$_{Fmoc}$

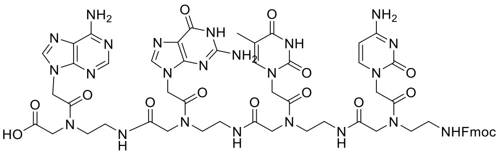

LCMS-ESI (m/z): $[M+H]^+$, calcd. for $C_{58}H_{65}N_{23}O_{15}$, 1324.51; found, 1324.58

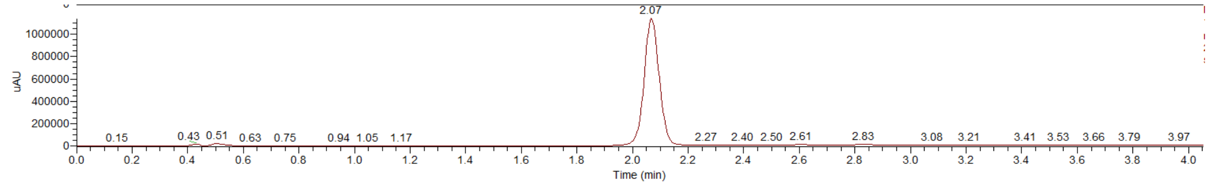

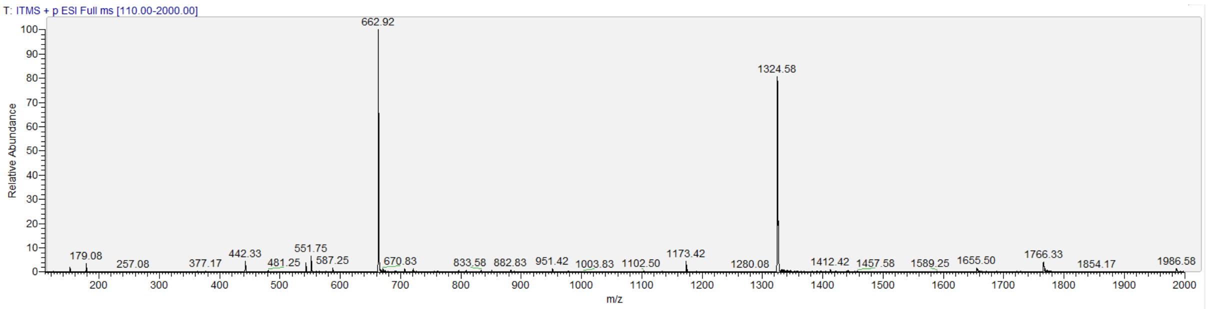

4-mer library:

AxxC$_{N3}$ – 16 compounds

LC-MS:

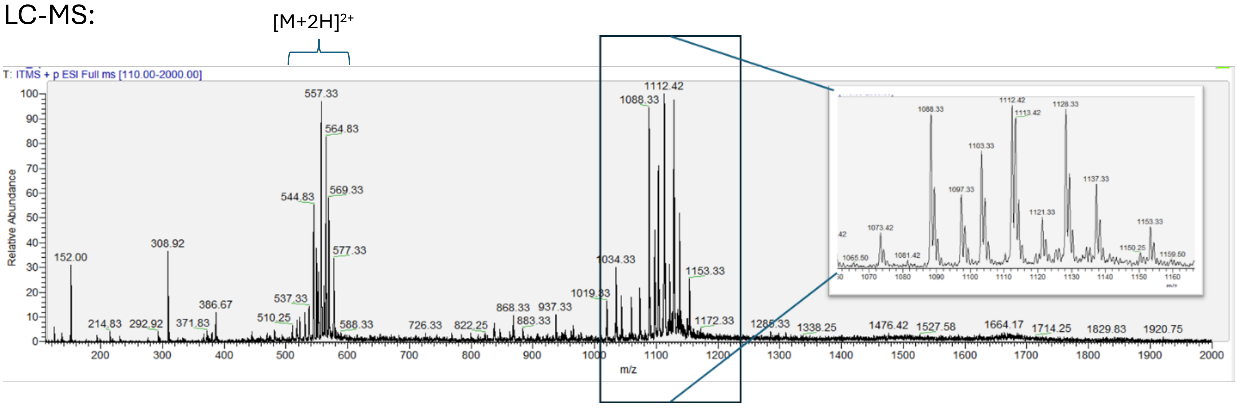

MALDI-TOF:

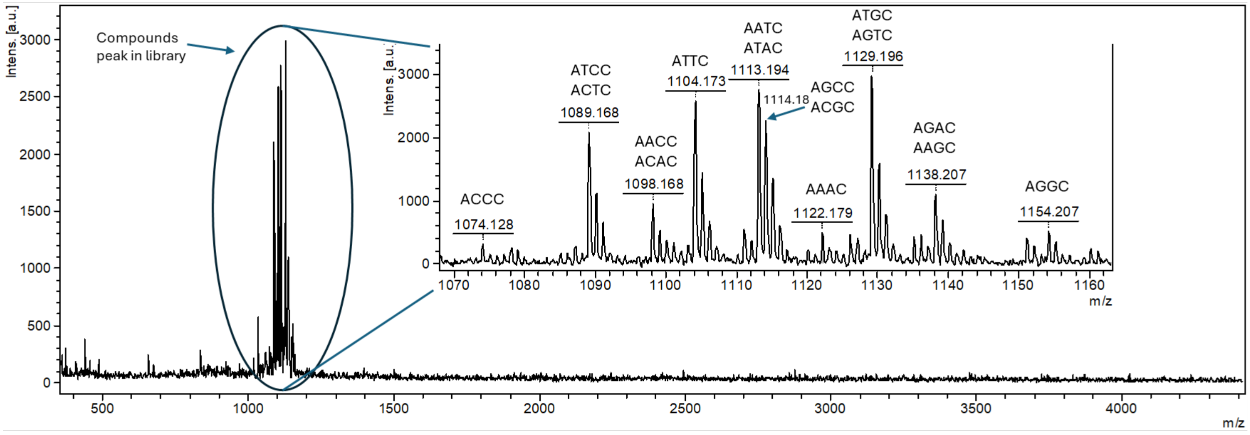

TxxG$_{N3}$ - 16 compounds

LC-MS:

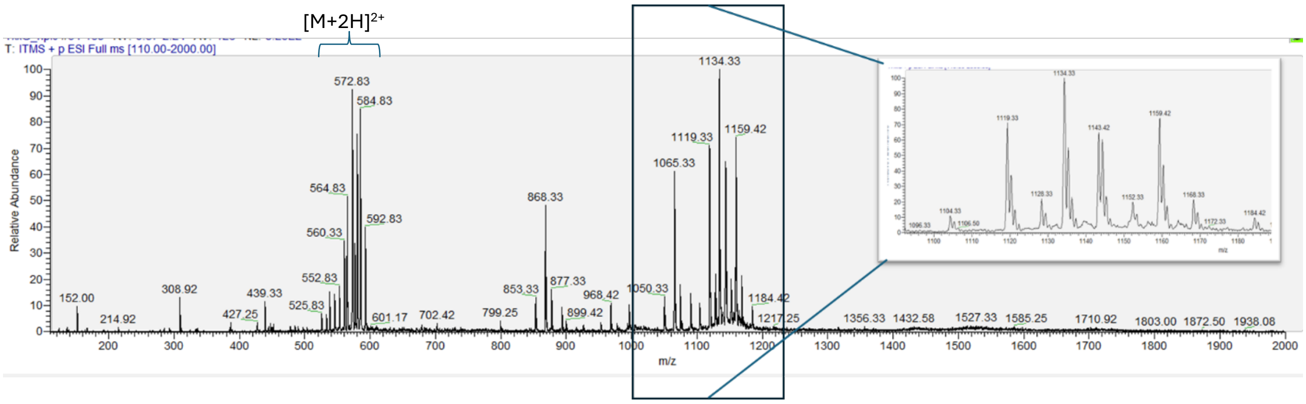

MALDI-TOF:

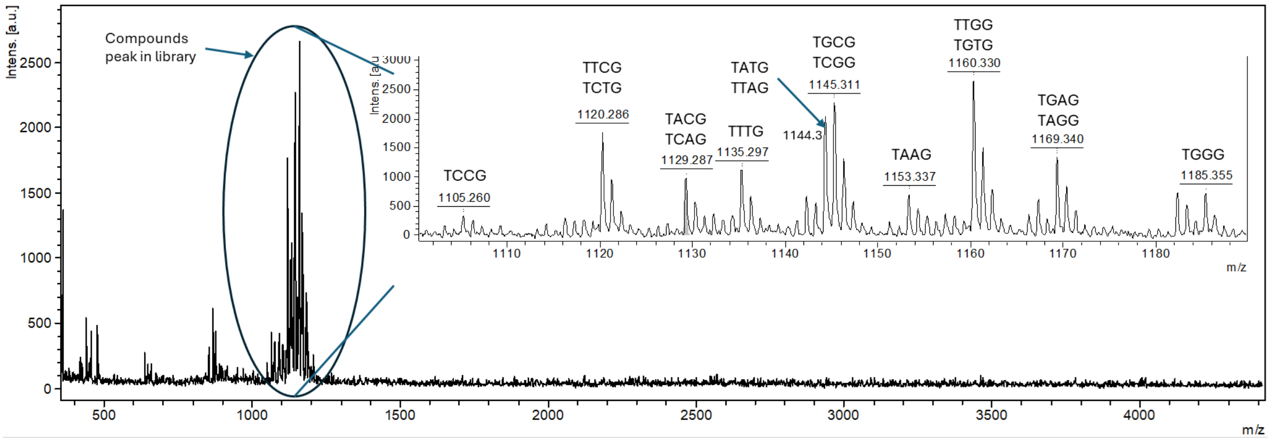

CxxA$_{N3}$ - 16 compounds

LC-MS:

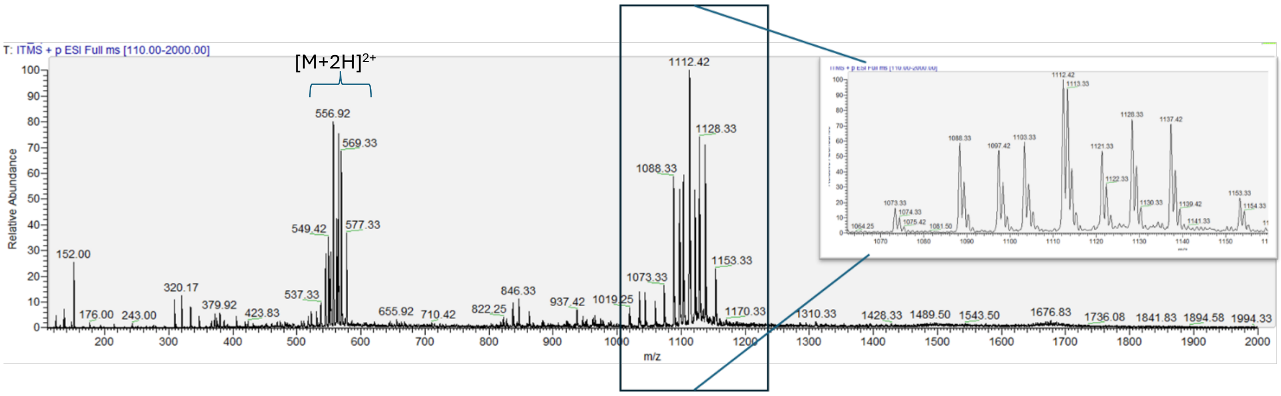

MALDI-TOF:

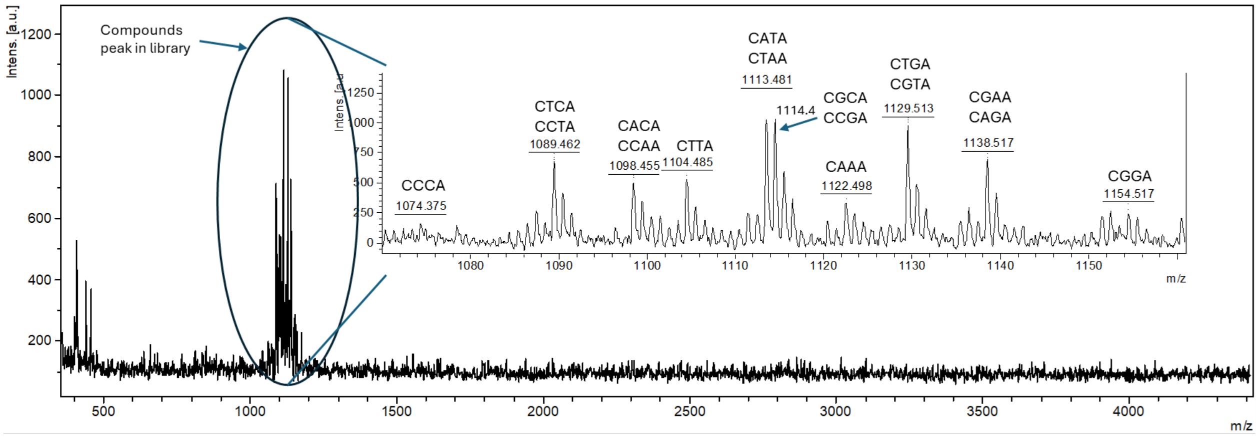

GxxT$_{N3}$ - 16 compounds

LC-MS:

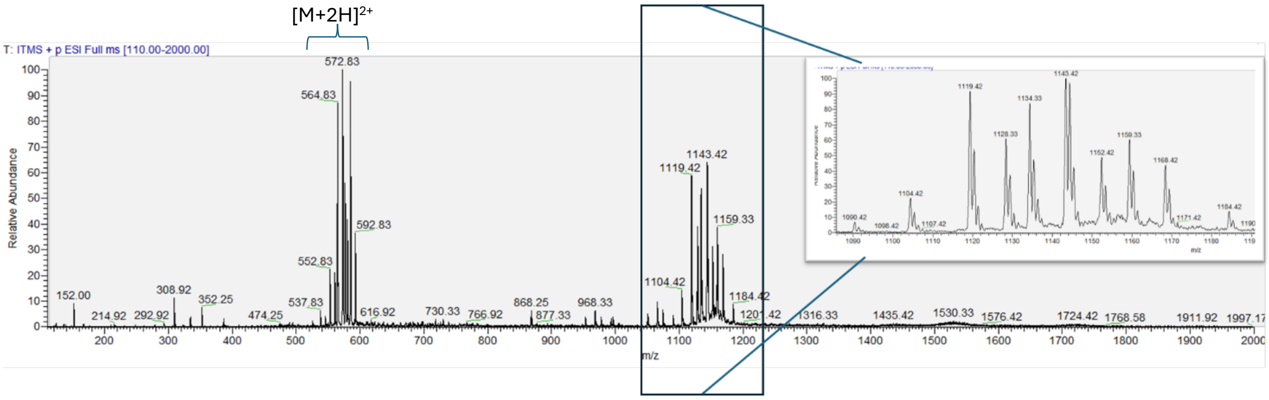

MALDI-TOF:

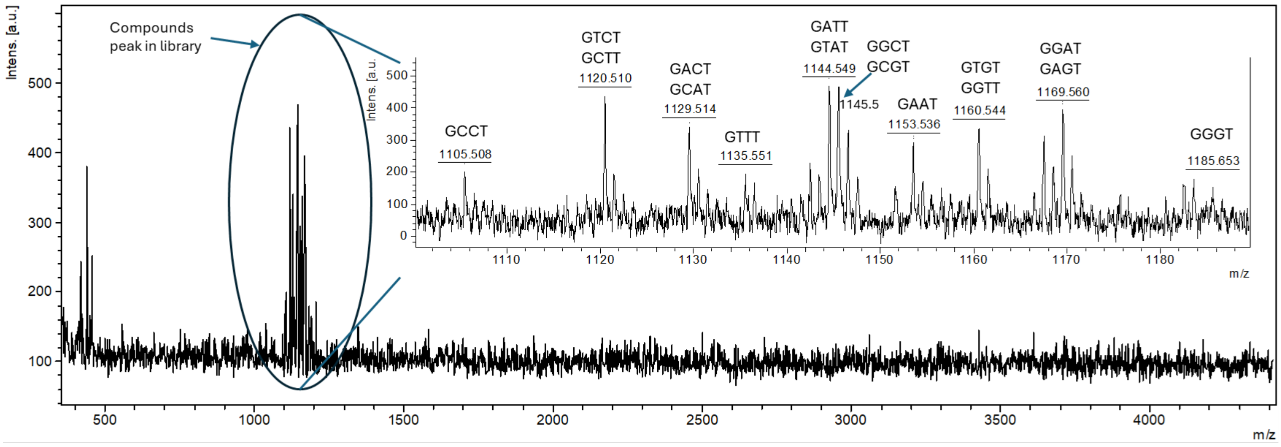

Monomers: Monomers were synthesized by previously established protocol[1]

AN$_3$:

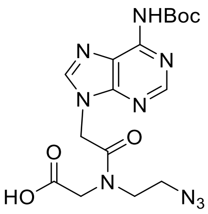

LCMS-ESI (m/z): [M+H]$^+$, calcd. for $C_{16}H_{21}N_9O_5$, 420.17; found, 419.81

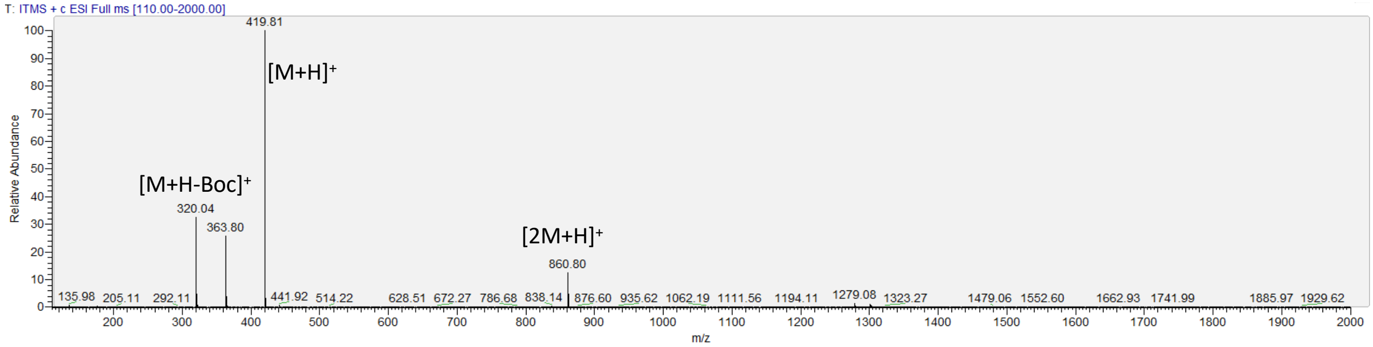

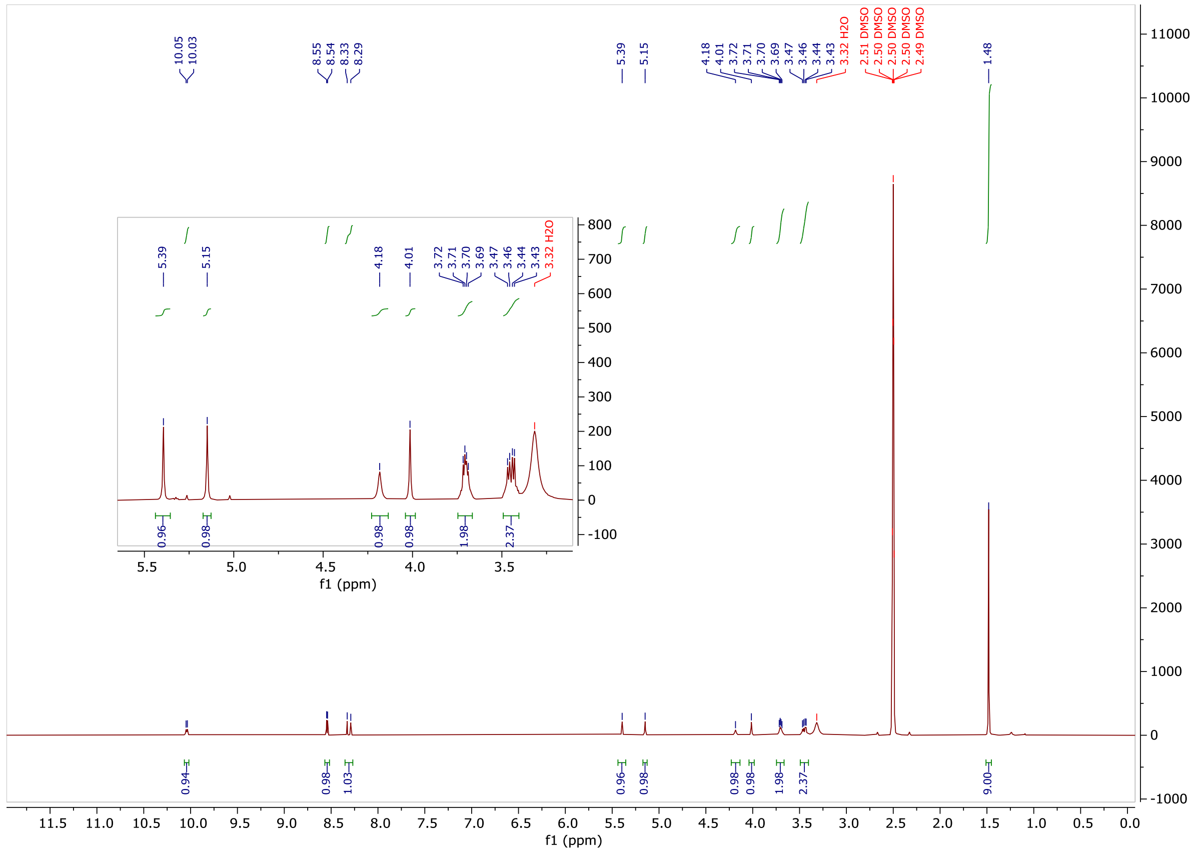

^{1}H NMR of AN_3

CN_3:

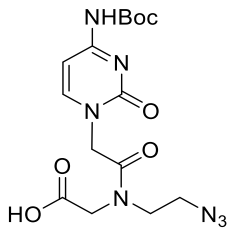

LCMS-ESI (m/z): $[M+H]^{+}$, calcd. for $C_{15}H_{21}N_7O_6$, 396.16; found, 395.85

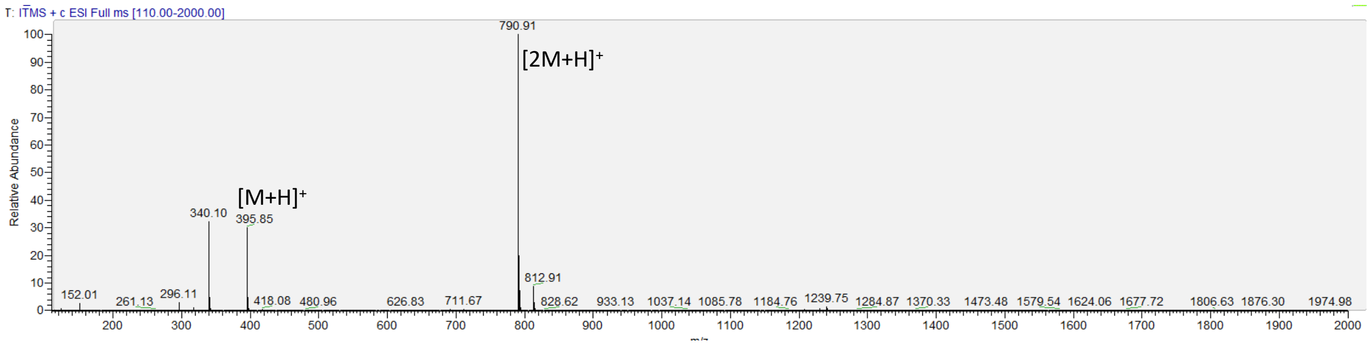

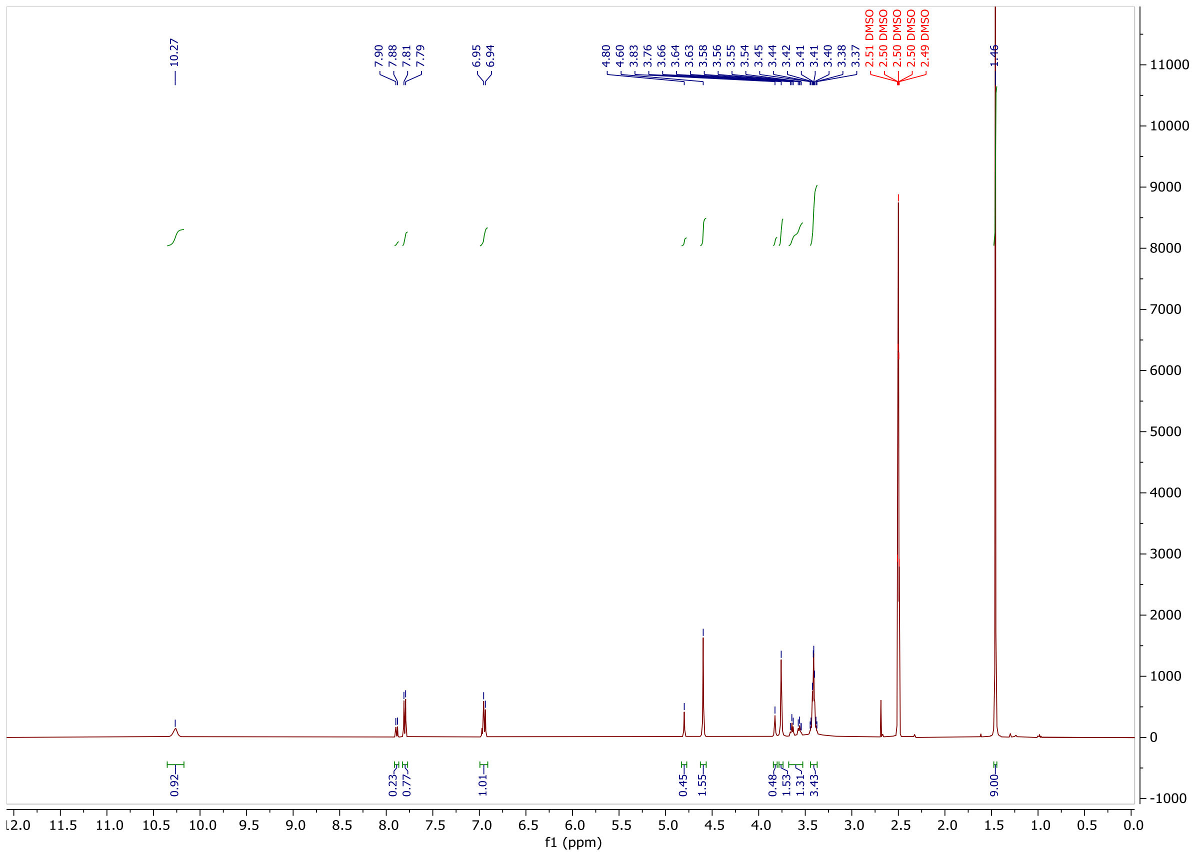

^{1}H NMR of CN_3

TN_3:

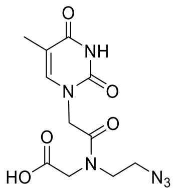

LCMS-ESI (m/z): $[M+H]^+$, calcd. for $C_{11}H_{14}N_6O_5$, 311.11; found, 310.92

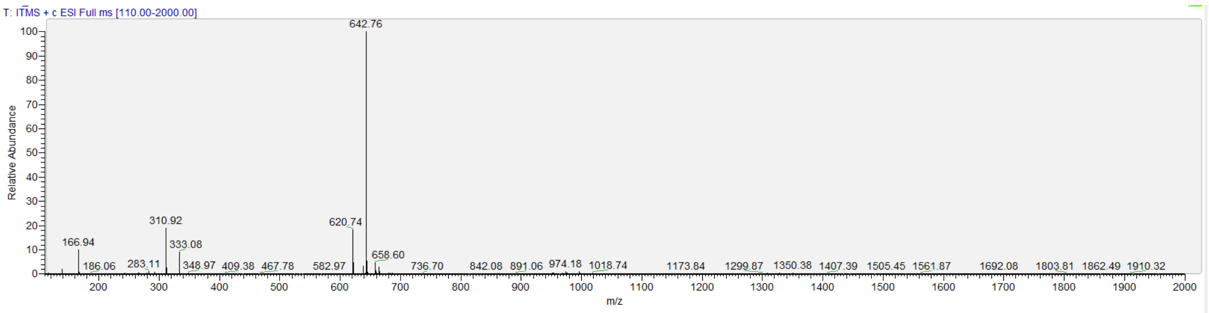

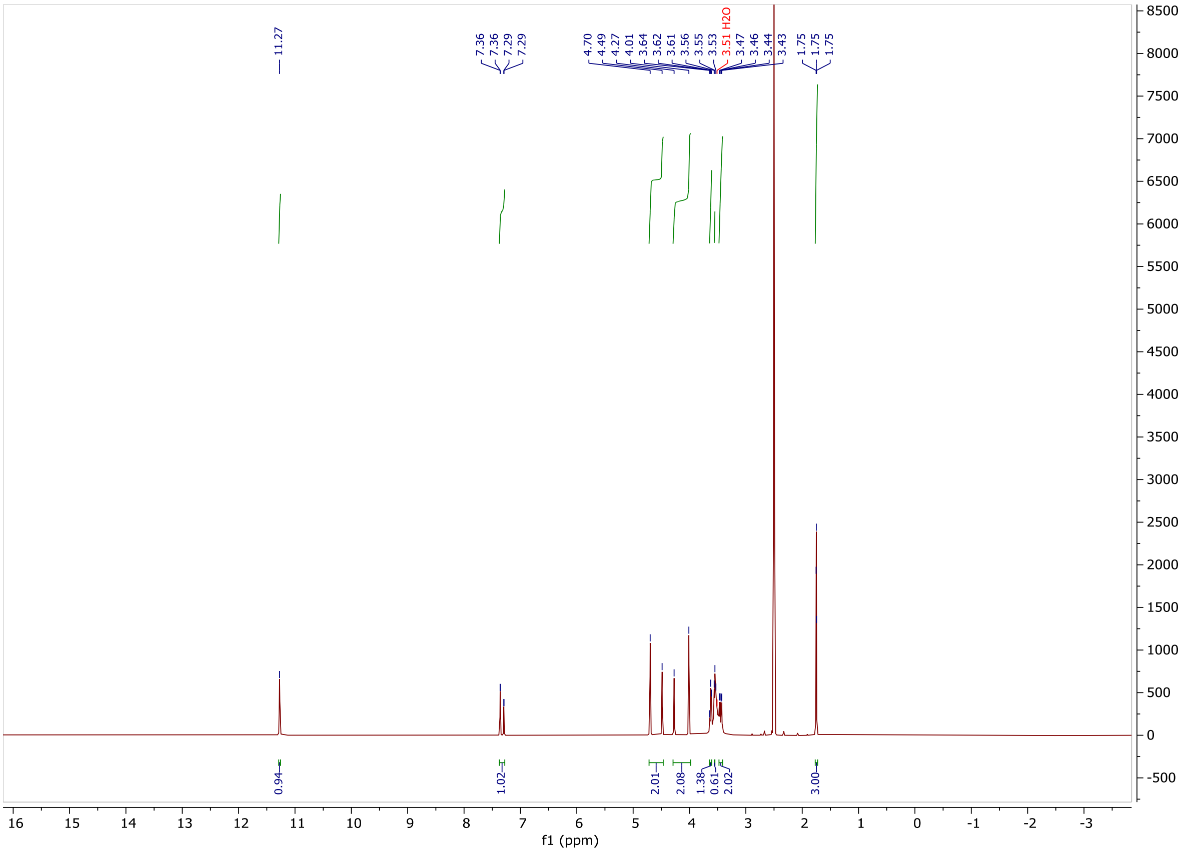

^{1}H NMR of TN_3

GN_3:

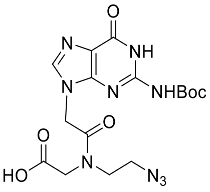

LCMS-ESI (m/z): $[M+H]^+$, calcd. for $C_{16}H_{21}N_9O_6$, 436.17; found, 435.92

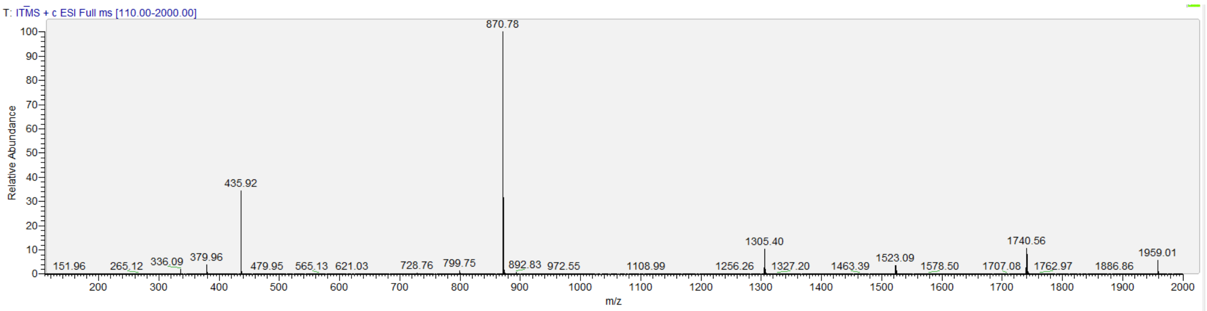

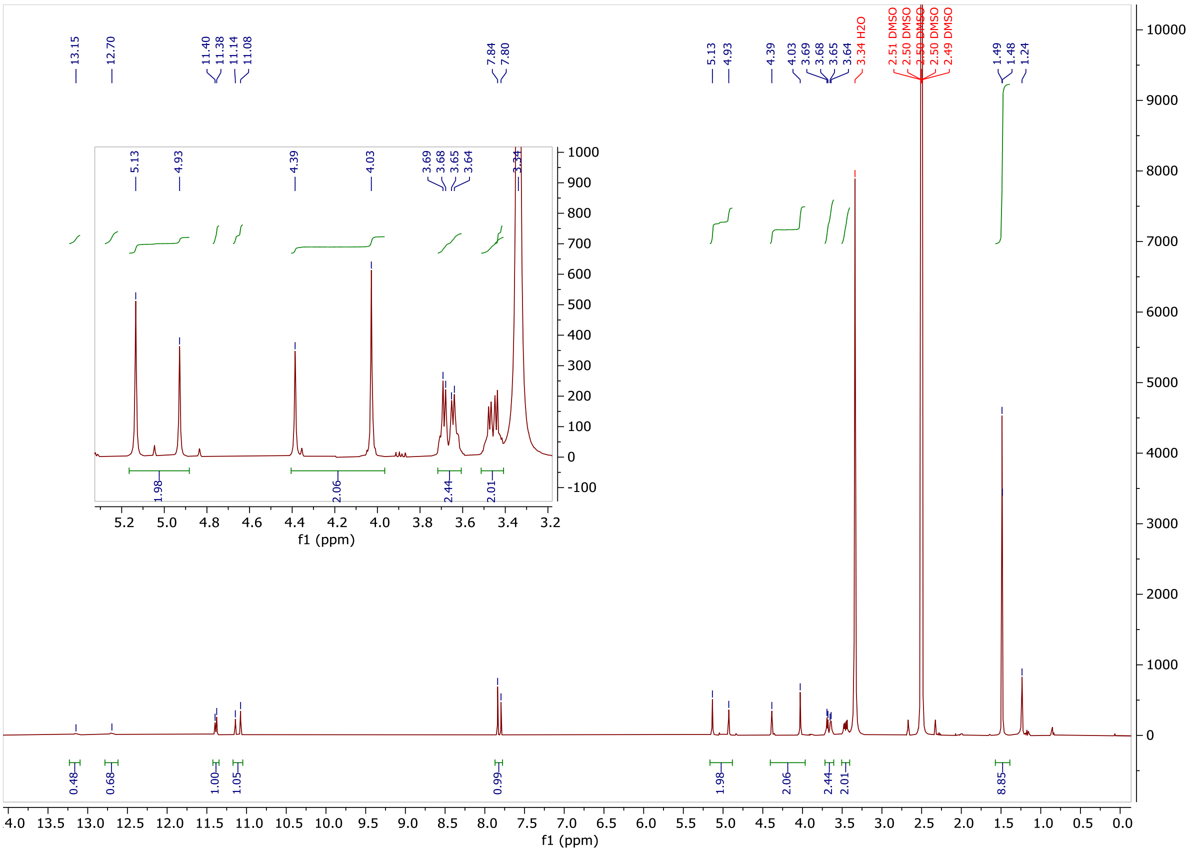

^{1}H NMR of GN_3

(1) Chouikhi, D.; Barluenga, S.; Winssinger, N. Clickable peptide nucleic acids (cPNA) with tunable affinity. *Chemical Communications* **2010**, *46* (30), 5476-5478, 10.1039/C0CC01081B. DOI: 10.1039/C0CC01081B.
